# Transcriptome Analysis of the Model Protozoan, *Tetrahymena thermophila*, Using Deep RNA Sequencing

**DOI:** 10.1371/journal.pone.0030630

**Published:** 2012-02-07

**Authors:** Jie Xiong, Xingyi Lu, Zhemin Zhou, Yue Chang, Dongxia Yuan, Miao Tian, Zhigang Zhou, Lei Wang, Chengjie Fu, Eduardo Orias, Wei Miao

**Affiliations:** 1 Key Laboratory of Aquatic Biodiversity and Conservation, Institute of Hydrobiology, Chinese Academy of Sciences, Wuhan, People's Republic of China; 2 Tianjin Economic-Technological Development Area School of Biological Sciences and Biotechnology, Nankai University, Tianjin, People's Republic of China; 3 Department of Molecular, Cellular and Developmental Biology, University of California Santa Barbara, Santa Barbara, California, United States of America; 4 Feed Research Institute of Chinese Academy of Agricultural Sciences, Beijing, People's Republic of China; 5 Graduate School of Chinese Academy of Sciences, Beijing, People's Republic of China; J. Craig Venter Institute, United States of America

## Abstract

**Background:**

The ciliated protozoan *Tetrahymena thermophila* is a well-studied single-celled eukaryote model organism for cellular and molecular biology. However, the lack of extensive *T. thermophila* cDNA libraries or a large expressed sequence tag (EST) database limited the quality of the original genome annotation.

**Methodology/Principal Findings:**

This RNA-seq study describes the first deep sequencing analysis of the *T. thermophila* transcriptome during the three major stages of the life cycle: growth, starvation and conjugation. Uniquely mapped reads covered more than 96% of the 24,725 predicted gene models in the somatic genome. More than 1,000 new transcribed regions were identified. The great dynamic range of RNA-seq allowed detection of a nearly six order-of-magnitude range of measurable gene expression orchestrated by this cell. RNA-seq also allowed the first prediction of transcript untranslated regions (UTRs) and an updated (larger) size estimate of the *T. thermophila* transcriptome: 57 Mb, or about 55% of the somatic genome. Our study identified nearly 1,500 alternative splicing (AS) events distributed over 5.2% of *T. thermophila* genes. This percentage represents a two order-of-magnitude increase over previous EST-based estimates in *Tetrahymena*. Evidence of stage-specific regulation of alternative splicing was also obtained. Finally, our study allowed us to completely confirm about 26.8% of the genes originally predicted by the gene finder, to correct coding sequence boundaries and intron-exon junctions for about a third, and to reassign microarray probes and correct earlier microarray data.

**Conclusions/Significance:**

RNA-seq data significantly improve the genome annotation and provide a fully comprehensive view of the global transcriptome of *T. thermophila*. To our knowledge, 5.2% of *T. thermophila* genes with AS is the highest percentage of genes showing AS reported in a unicellular eukaryote. *Tetrahymena* thus becomes an excellent unicellular model eukaryote in which to investigate mechanisms of alternative splicing.

## Introduction

Although unicellular, *Tetrahymena thermophila* possesses most of the conserved cell structures and molecular processes found in multicellular eukaryotes. In particular *Tetrahymena* has many orthologs of human proteins not found in other unicellular models such as yeast [1]. A number of fundamental discoveries of molecular biology were made in this ciliate protozoan, including the nature of telomeres [2], telomerase [3] and self-splicing RNA [4], the first demonstration that a transcription factor was a histone modifying enzyme [5] and one of the first demonstrations of the role small RNAs in heterochromatin formation [6], [7]. As a eukaryotic model system, *T. thermophila* grows rapidly to a high cell density in a variety of media and conditions and allows the convenient use of advanced molecular genetic tools such as RNA interference (RNAi), gene overexpression and knock-out [8].

Like most other ciliated protozoans, *Tetrahymena* is a binucleated cell with a germline micronucleus (MIC) and a somatic macronucleus (MAC) [9]. The MIC is diploid, contains 5 pairs of chromosomes and is transcriptionally inert during most of the life cycle. The MAC is transcriptionally active and contains ∼45 copies each of ∼200 chromosomes derived by site-specific fragmentation and amplification from the 5 MIC chromosomes when the MAC develops from the MIC during the sexual process of conjugation.

The first analysis of the ∼104 Mb *Tetrahymena* MAC genome sequence [1] (hereafter also called the 2006 release) predicted 27,424 protein-coding gene models. Subsequent analysis refined the assembly and annotation through comparative genomic hybridization, targeted gap closure and sequence data from about 60,000 ESTs, resulting in the current genome annotation version (hereafter also called the 2008 release) with 24,725 gene models [10]. Miao *et al.* (2009) used a single channel microarray platform (Roche NimbleGen) to measure the transcription level of all of the predicted genes at 20 time points during the three major physiological/developmental stages of *Tetrahymena* (growth, starvation and conjugation) [11]. However, a serious lack of extensive cDNA sequences limited the accuracy of predicted gene models, thus partially reducing the usefulness of the cDNA microarray data, whose probes were designed based largely on the 2006 predicted open reading frames (ORFs).

Deep RNA sequencing (RNA-seq) using second generation sequencing techniques (e.g., Illumina's Genome Analyzer II) provides an unbiased, comprehensive method to understand the transcriptome of an organism [12], [13], and is more sensitive than microarray methods [14], [15]. The transcriptomes of several other eukaryotes, including humans [16], [17], yeast [18], *Arabidopsis* [19], rice [20] and *Plasmodium* [21], have been determined by RNA-seq. By assembling transcripts from sequenced reads that map to a reference genome, the RNA-seq approach can greatly improve the accuracy of genome annotation and identify untranslated regions (UTR), novel transcripts and alternative splicing; it can also reveal additional gene expression information [22], [23], such as in mammalian [17], rice [20] and *Candida albicans* [24].

The splicing of precursor mRNA not only reconstitutes gene coding sequence, but is also an important regulatory step in the process of gene expression in eukaryotes [25]. Alternative splicing (AS) is a major means by which eukaryotes greatly increase their transcriptome and proteome diversity for a given number of genes [26], [27], [28]. High-throughput RNA-seq technology provides an efficient opportunity to globally investigate AS and it has revealed a high amount of AS in plants [19]–[20] and mammals [29].

In this study, we present a comprehensive analysis of the transcriptome of *T. thermophila* using the Illumina RNA-seq platform. The data were generated from six mRNA samples, one from growing, three from starving and two from conjugating cells. A total of about 125 million reads were mapped to the *T. thermophila* genome. These allowed us to significantly improve the previous genome annotation and re-investigate gene expression profiles. Our results also showed that alternative splicing in *T. thermophila* occurs far more frequently than previously reported.

## Results

### Deep RNA sequencing of *T. thermophila*

To better understand the *T. thermophila* transcriptome and gene expression, we performed high-throughput RNA-seq for six Poly-A-purified RNA samples from three major physiological or developmental stages of *T. thermophila*: growth, starvation and conjugation (see Methods). We obtained about 94 million paired-end reads, with a total length of more than 14 gigabases (Gb) (Table 1). About 123 million (65.6%) of the reads could be uniquely mapped to the *T. thermophila* reference genome (Table 1). They cover 57 megabases (Mb) of sequence, which represents about 55% of the macronuclear genome. The previous estimate, based on the initial set of predicted genes without 5′ and 3′ untranslated regions, was 48.9 Mb [10]. In the remainder of this article, by “mapped reads” we mean “uniquely mapped reads”.

**Table 1:** RNA-seq mapping statistics.

Sample	Reads length (bp)	Reads generated	Base generated	Unique mapped reads
G-m	53	61,625,410	3,266,146,730	35,553,047
S-3 (mating type VI)	75	31,956,872	2,396,765,400	20,044,802
S-3 (mating type V)	100	17,878,254	1,787,825,400	11,166,805
S-15	100	16,509,042	1,650,904,200	11,402,254
C-2	75	32,658,040	2,449,353,000	23,060,144
C-8	100	27,384,596	2,738,459,600	22,022,362
Total	/	188,012,214	14,289,454,330	123,249,414

The samples G-m, S-3, S-15, C-2 and C-8 refer to growth to mid-log cell density (∼3.5×10^5^ cells/ml), 3 hours of starvation, 15 hours of starvation, 2 hours into conjugation and 8 hours into conjugation, respectively.

**Figure 1:**
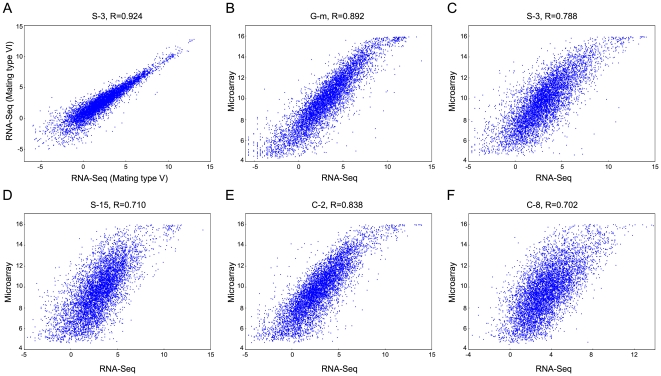
Correlation between RNA-seq and microarray data sets. For RNA-seq, the LOG2 RPKM values were used; for microarrays, LOG2 fluorescence intensity values were used. The states G-m, S-3, S-15, C-2 and C-8 refer to growth to mid-log cell density (∼3.5×10^5^ cells/ml), 3 hours of starvation, 15 hours of starvation, 2 hours into conjugation and 8 hours into conjugation, respectively. Note the sigmoidal shape of the correlation curves, attributed to higher dynamic range of RNA-seq relative to microarrays at both ends of the gene expression range.

RNA-seq is a highly accurate technique resulting in high correlation among replicate samples [14], [18]. Indeed we found a very high correlation (R = 0.924) between RNA-seq data obtained from two isogenic strains of *T. thermophila* (Figure 1A), both starved for 3 hours and differing only in mating type (see Methods). These samples are essentially biological replicates and further demonstrate the reproducibility of RNA-seq.

Reads from the six combined RNA-seq data sets mapped to 96.1% (23,770 of 24,725) of the previously annotated open reading frames (ORFs) in the genome. Most of the 955 predicted genes not detected by RNA-seq share the following properties in various combinations: they are generally hypothetical genes without conserved domains, less than 100-codons long and showed no detectable gene expression in the microarray study (see more detailed analysis of these unconfirmed genes in Section 1 of Text S1, Table S1 and Table S2). Most of these previously undetected gene models are likely to be previously mispredicted genes, pseudogenes, or genes transcribed only in response to untested physiological conditions.

**Figure 2:**
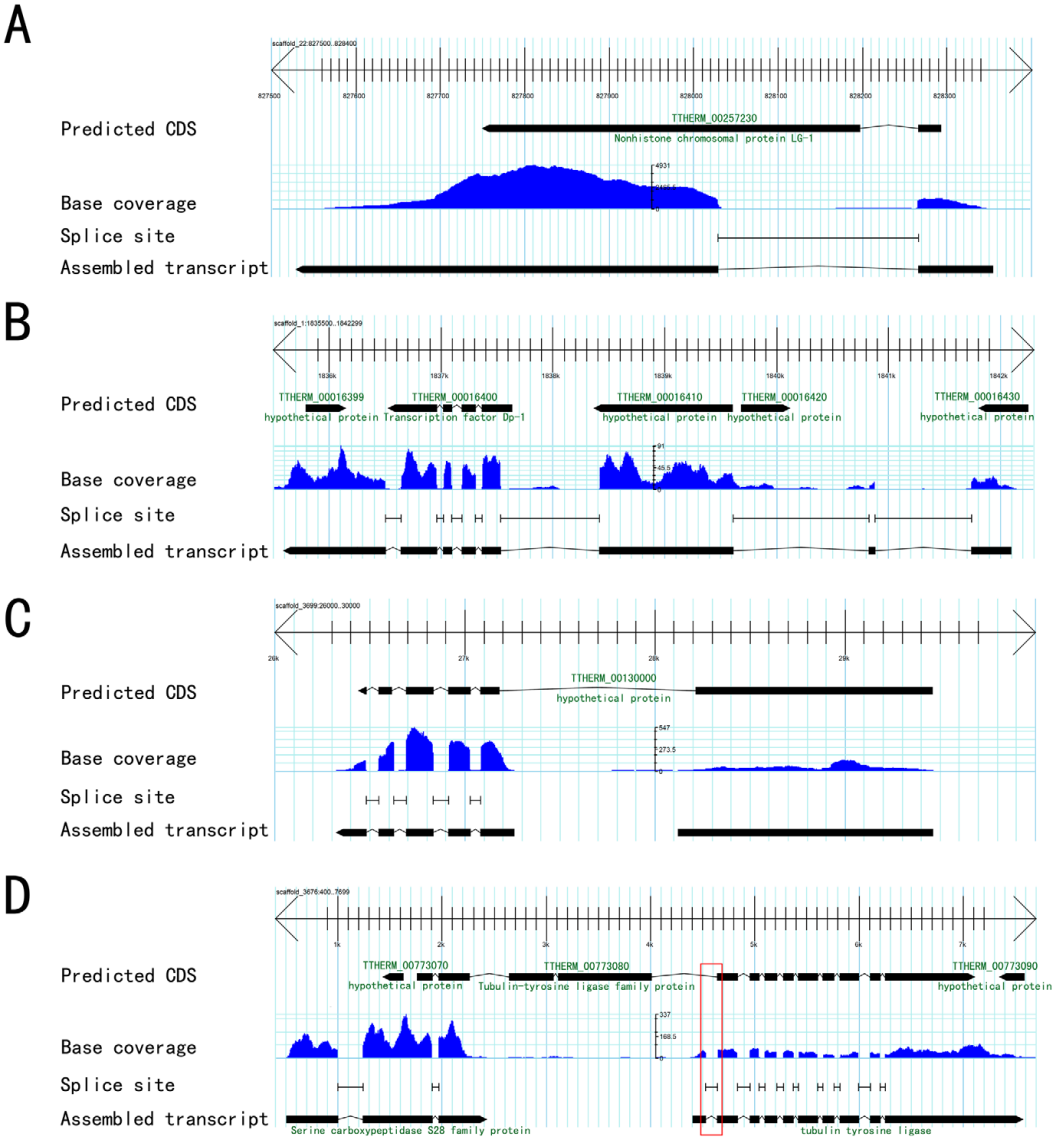
Gbrowse snapshot showing examples of mis-predictions in the current *T. thermophila* genome annotation that were corrected by RNA-seq. A, mis-predicted gene TTHERM_00257230 with incorrect intron boundary; note that original gene annotations did not include UTRs. B, a region previously annotated as containing five predicted genes has only one gene; C, a region previously annotated as one predicted gene (TTHERM_00130000) contains two genes, as further indicated by the coverage discontinuity between the two assembled transcripts strongly argues for two genes. D, a gene prediction with multiple errors, including a mis-predicted splice site (red box), spurious exons and an incorrect gene fusion. There is good functional annotation support for these two transcripts: their top BLASTP matches are to “tubulin tyrosine ligase (E-value: 1e-113)” and “serine carboxypeptidase S28 family protein (E-value: 3e-110)” respectively.

Mapped reads were used to assemble a total of 31,116 transcripts, which were related to 20,206 previously annotated gene regions. 26.8% (6,633) of previously predicted gene models were completely confirmed by the RNA-seq data. Approximately 7,300 gene models were found to have mispredictions. These included incorrect intron/exon boundaries, missing or extra introns, and incorrectly split or fused genes. Examples are illustrated in Figure 2. Altogether, over 7,290 predicted gene models were corrected.

Splice sites are reliably identified with the help of reads that span exon-exon junctions. The latest *T. thermophila* genome annotation contains 24,725 protein coding genes with 89,302 predicted splice sites. Among our mapped reads, 2,936,024 non-redundant reads spanned exon-exon junctions, representing 106,937 canonical GT-AG splice sites supported by at least one read.

Detailed comparison shows that only 48.3% (51,652) of RNA-seq predicted splice sites exactly matched those in the 2008 release. Among RNA-seq predicted introns with at least one previously unpredicted splice site, only 49.9% overlapped introns in the latest prediction. Among these, 56.6% shared one (5′ or 3′) splice site. Comparison of introns predicted by RNA-seq and by the 2008 release showed systematic biases. The average length of RNA-seq predicted introns was smaller (135 bp vs. 162 bp) and the introns showed a small (about 2 percentage points) but statistically significant increase in G and C nucleotide abundance (Table S3A). The cause of these differences is not clear, but could be related to the fact that the *ab initio T. thermophila* gene model prediction [1] partially used parameters derived from the Apicomplexan *Plasmodium* to compensate for the short supply of *T. thermophila* ESTs. At the time *Plasmodium* was the closest Alveolate with a genome sequence, but it has a genomic A+T content higher than that of *T. thermophila*.

### Novel transcribed regions

**Figure 3:**
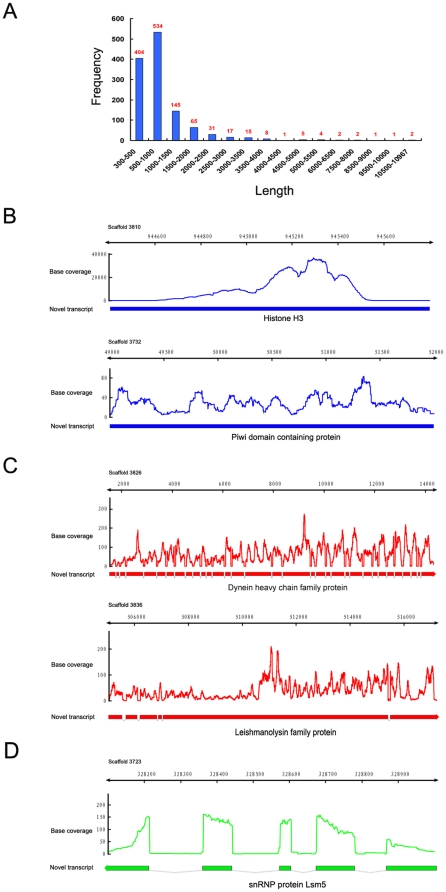
RNA-seq indentified novel transcribed regions in *T. thermophila*. A, Length (bp) distribution of novel transcribed regions; B, two important novel genes, a histone H3 and piwi domain-containing gene; C, the two longest novel transcribed regions; D, a novel transcribed region encode the U6 snRNA associate LSm5 protein. Exons are represented as bars; the arrowhead indicates the direction of transcription. Introns are shown as hatched lines. The direction of transcription is not shown for intron-less transcripts.

We used our RNA-seq data to search for novel transcribed regions, not predicted by the existing genome annotation. We wished to exclude apparent novel regions which could simply be 5′ or 3′ UTRs fractured from coding region assemblies because of insufficient coverage. Therefore, a novel transcribed region was defined as an assembled transcript which completely matched an intergenic region with a length at least 300 bp, as most of the 5′ and 3′ UTRs are shorter than that (see below). Using this criterion, we identified 1,237 novel transcribed regions, with an average length of 918 bp (Figure 3A). 217 of these turned out to encode proteins predicted in the 2006 release but missing in the 2008 release. The rest, 1056 transcripts, represent totally new transcribed regions. Most of these (∼70%) code for proteins, ∼90% of which are members of multigene families. The remaining 30% may be transcribed fragments which lacked a long recognizable ORF. A more detailed analysis of newly discovered transcribed regions is presented in Section 2 of Text S1 (Table S4 and Table S5).

### Untranslated region (UTR) predictions

No transcriptome-wide UTR predictions had previously been made in *T. thermophila*. RNA-seq data are very useful for identifying the structural boundaries of genes, as coverage plots tend to taper gradually toward the 5′ and 3′ ends of the 5′ and 3′ UTRs, respectively, whereas they drop abruptly towards zero in intron regions. We investigated possible UTRs using our RNA-seq data. To use only the most credible ORFs, only the 6,633 gene models predicted in the 2008 release and confirmed by our RNA-seq data were inspected. Predicted 5′ and 3′ UTRs of these 6,633 genes covered a total of 0.75 and 1.34 Mb, respectively. These numbers are likely underestimates, as coverage decreases and reads may be missing by chance alone at the outer ends. Nevertheless, if these values are extrapolated to the total number of predicted genes (27,425 genes), UTRs would cover about 13.7% of the transcriptome. Examination of the UTR length distribution revealed that 94% of 5′ UTRs and 83% 3′ UTRs were shorter than 300 bp (Figure S1).

### Gene expression profiling based on RNA-seq

RNA-seq data are reported to highly correlate with microarray data [14], [21], [24]. To compare our RNA-seq data to the single channel Roche NimbleGen microarray data from *T. thermophila* cells at identical physiological/developmental states, we used reads per kilobase of exons per million mapped reads (RPKM) values for each confirmed gene model as a quantitative estimate of expression. Our results showed that the two methods have high Pearson correlation coefficients, ranging from 0.702 to 0.892 (Figure 1B–F), in agreement with previous studies [14], [21]. However, the RNA-seq technique allows more sensitive detection of low expressed genes than the microarray technique, as indicated by the flattened initial portion of the curves in Figure 1B–F. With RNA-seq, expression of only 3.8% of the predicted genes was undetected in all six conditions/time points. This percentage is significantly lower than the 22% of annotated genes whose expression was below the background value in all of the much larger number (20) of conditions/time points analyzed in the single channel microarray experiments [11]. The flattened upper end of the curve similarly indicates that the RNA-seq method has a larger discriminating power also for very highly expressed genes, whose microarray signal saturates the fluorescence scanner. Thus the RNA-seq measurements show a larger dynamic range than microarrays at both ends of the broad expression level spectrum displayed by *T. thermophila*.

**Figure 4:**
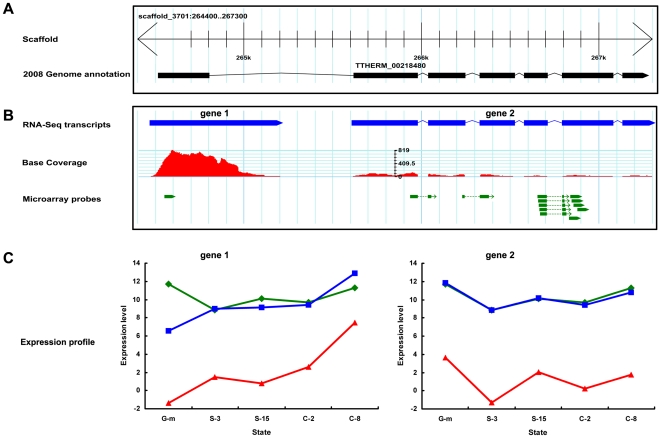
Reassignment of microarray probes and renormalization of microarray expression values using RNA-seq corrected gene models. A, a gene model of *T. thermophila* (TTHERM_00218480) as originally annotated; B, corrected gene models based on RNA-seq data where the green bars indicate microarray probes; C, gene expression profiles, green, microarray expression profile using signals of probes based on the incorrect gene model; blue, renormalized microarray expression profile using signal from probes reflecting the corrected gene model; red, expression profile of RNA-seq using RPKM values. The states G-m, S-3, S-15, C-2 and C-8 refer to growth to mid-log cell density (∼3.5×10^5^ cells/ml), 3 hours of starvation, 15 hours of starvation, 2 hours into conjugation and 8 hours into conjugation, respectively. The expression level was LOG2 transformed.

The microarray analysis used the 2006 genome annotation to design the hybridization probes, and was thus highly dependent on the accuracy of the gene models. On the other hand, the RNA-seq data, in addition to measuring gene expression levels, allowed the assembly of transcripts that were used to greatly improve the gene models. We used the RNA-seq corrected gene models to renormalize the microarray expression levels for mispredicted genes, by using only those probes located in the corrected gene models. For example, the 6-introns gene model TTHERM_00218480 spanned about 2.9 Kb in assembled MAC scaffold 3701 (Figure 4A). The coverage differences between two assembled transcripts showed that this gene was mispredicted and should be separated into two genes with very different expression profiles (Figure 4B). After sorting the microarray probes to the two genes, the re-normalized microarray expression profiles supported the RNA-seq results (Figure 4C).

**Figure 5:**
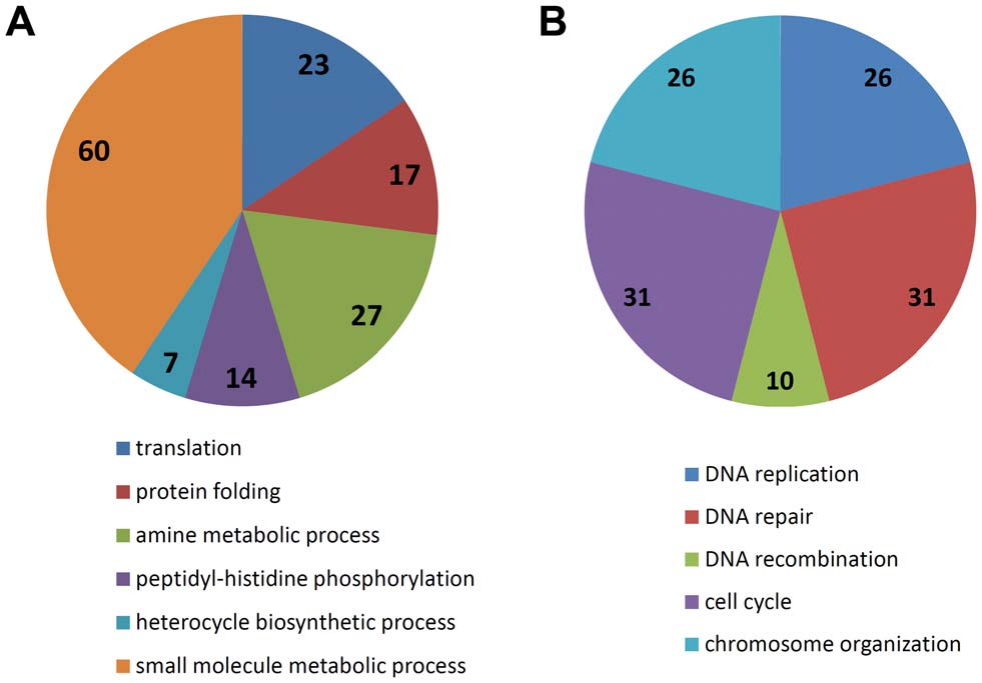
Representative overrepresented functions among genes which are up-regulated during growth and conjugation. A, growth; B, conjugation. Representative gene ontology terms were chosen using the parent-child relationships in Figure S2. The number of genes involved in each function is indicated in each sector. Sector colors in the two charts are unrelated to one another.

Growth, starvation, and conjugation are three major physiological and developmental states in *T. thermophila*. We used the RNA-seq data to look for genes with stage specific up-regulation (see methods). By measuring RPKM fold changes, we identified 1,002, 231, and 1,894 genes specifically up-regulated during growth, starvation, and conjugation, respectively. The functional annotation of each gene was carried out using gene ontology (GO) biological process information; enrichment analysis [30] was performed for up-regulated genes at each stage. For growth, 25 biological process GO terms are significantly overrepresented; they are primarily involved in small molecule metabolism, amine catabolism, heterocycle biosynthetic process, translation, protein folding and peptidyl-histidine phosphorylation (Figure 5 and Figure S2A). No overrepresented GO terms were found for starvation, as few of the 62 starvation-specific genes could be annotated by GO information. For conjugation, 28 biological process GO terms were overrepresented; most are typically related to this stage, such as DNA replication, DNA repair and DNA recombination, cell cycle and chromosome organization (Figure 5 and Figure S2B).

### Identification of alternative splicing events

It has been proposed that AS is more prevalent in multicellular than in unicellular eukaryotes [31]. Indeed, less than 5% of genes undergo AS events among unicellular eukaryotes so far investigated (Table 2). Using RNA-seq to detect splice sites, we sought to identify AS events in the unicellular organism *T. thermophila*. Using stringent criteria to detect AS (see methods), 1,286 (5.2%) *T. thermophila* genes generated AS isoforms, among which 105 genes showed at least two AS events. In total, 1,474 AS events were identified. We found all four basic types of AS events [32], illustrated in Figure S3: cassette-exon inclusion or skipping (139 events), alternative 5′ splice-site selection (258 events), alternative 3′ splice-site selection (245 events), and intron retention (IR) (832 events, the majority type). 46 of 50 putative AS events detected by RNA-seq were validated by RT-PCR (data not shown); the four failures are attributed to low expression. The higher frequency of IR (nearly 6 times) relative to cassette-exon inclusion or skipping AS events observed in *Tetrahymena* suggests that *intron* definition is most often used as the mechanism of splice site recognition, rather than *exon* definition, as found in humans [33]. This difference correlates well with *Tetrahymena* introns being generally smaller than exons, while the reverse is true in humans. In *Tetrahymena,* only ten cases of AS had been previously reported in a study using EST data [10]. These consisted of six intron retention, two 5′ splice-site selection and two alternative 3′ splice-site selection events. Each of these AS events was confirmed by our RNA-seq data. Thus, RNA-seq has increased by two orders of magnitude the number of previously known AS events in *T. thermophila*.

**Table 2:** Incidence of AS genes in diverse eukaryotes.

Organism	Evolutionary Group	Total gene number^b^	Total Intron number^c^	Average Introns per genes	AS/Total genes %	AS Method & Reference
*Homo sapiens*	metazoa	20,834	258,085	12.39	95%	RNA-seq [32]
*Mus musculus*	metazoa	23,200	209,066	9.01	45%	EST [56]
*Bos taurus*	metazoa	22,787	190,317	8.35	21%	Bioinformatics [57]
*Danio rerio*	metazoa	26,564	240,336	9.05	17%	EST [58]
*Caenorhabditis elegans*	metazoa	20,183	109,160	5.41	25%	RNA- seq & Microarray [59]
*Drosophila melanogaster*	metazoa	13,776	45,796	3.32	54.20%	RNA-seq [60]
*Oryza sativa*	plant	28,453	129,751	4.56	33%	RNA-seq [20]
*Arabidopsis thaliana*	plant	27,361	120,155	4.39	36.40%	RNA-seq [19]
*Chlamydomonas reinhardtii*	plant (unicellular)	14,416	104,665	7.26	3%	EST [61]
*Aspergillus oryzae*	fungi^a^	12,691	29,473	2.32	8.10%	RNA-seq [62]
*Tuber melanosporum*	fungi	7,496	21,525	2.87	15.4	RNA-seq [63]
*Cryptococcus neoformans*	fungi (unicellular)	6,273	35,122	5.60	4.20%	EST [64]
*Saccharomyces cerevisiae*	fungi (unicellular)	5,882	358	0.06	few cases	Not described [40]
*Phytophthora sojae*	Oomycetes (unicellular)^a^	16,988	34,207	2.01	0.70%	EST [65]
*Dictyostelium discoideum*	Amebozoa (unicellular)	13,289	16,869	1.27	0.10%	Not described [66]
*Plasmodium falciparum*	Apicomplexa (unicellular)	5,331	8,826	1.66	4.50%	RNA-seq [67]
*Paramecium tetraurelia*	Ciliate (unicellular)	40,043	90,574	2.26	0.90%	EST [42]
*Tetrahymena thermophila*	Ciliate (unicellular)	24,725	89,302	3.61	5.20%	RNA-seq, this work

a With hyphal stage in the life cycle.

b Gene number retrieved from the KEGG genome statistics.

c The total intron numbers were determined using the GTF or GFF annotation file of each organism.

The average length (see methods) and other features of AS spliced introns were determined. Introns involved in cassette-exon inclusion or skipping showed the largest average length (279 bp), followed by those involved in alternative 3′ splice-site selection (208 bp), alternative 5′ splice-site selection (188 bp), and intron retention (80 bp). It is not yet clear what factors are major determinants of this length distribution. For example, weak vs. robust splice sites have not yet been identified in *Tetrahymena*. Indeed this work provides the first extensive evidence-based database of splice sites and AS events required for an experimental investigation of these questions in a model organism excellently suited for this purpose. Every type of AS event generated transcripts containing in-frame premature termination codons (PTCs) with frequencies that varied between 25% and 33% among the four types of AS events. In-frame PTCs may serve a posttranscriptional regulatory function through the nonsense-mediated mRNA decay (NMD) pathway (see Discussion section). In addition, a very slight but statistically significant difference in base composition was detected between AS and all introns (Table S3B), whose basis is not clear.

Unlike the first three listed classes of AS events, the intron retention (IR) class could be relatively non-specific, due, for example, to weak cis-acting motifs required for splicing or to a limited availability of splicing machinery. To probe this issue, we investigated the frequency and distribution of IR splicing events among genes encoding at least two introns. To make sure the data was reliable, we focused on the genes predicted in the 2008 release that were confirmed by this RNA-seq study without misprediction of splice sites. Cases in which all introns were retained in these transcripts are extremely rare. 2,698 genes encoding at least 2 introns and splice sites confirmed by RNA-seq data, every intron was retained in just one case (Table 3, columns D). Regardless of the number of encoded introns, at most one intron was retained in the vast majority of cases (Table 3, Columns D). Almost always, it was the same limited fraction of the introns that was retained (Table 3, columns E), suggesting that also intron retention in most cases is an AS class programmed by the transcript sequence.

**Table 3:** Distribution and specificity of intron retention type of AS events among genes encoding at least two introns.

A. Number of introns in the gene	B. Number of genes	C. Number of IR genes	D. Number of IR introns excised			E. Frequency of the most commonly retained intron^b^	
			None	1	2 to (n-1)^a^	Growth & Starvation	Conjugation
2	781	22	1	21	NA	0.93	0.89
3	499	15	0	15	0	0.91	0.93
4	388	22	0	19	3	0.92	0.9
5	322	19	0	18	1	0.92	0.89
6	233	9	0	9	0	0.91	0.86
7	175	14	0	10	4	0.87	0.93
8	116	9	0	7	2	0.92	0.9
>8	184	16	0	15	1	0.85	0.79
Total	2698	126	1	114	11	0.90^c^	0.89^c^

a One less than the maximum number of encoded introns.

b Calculated as the number of reads indicating retention of the most commonly retained intron divided by the number of reads indicating retention of *any* intron.

c Weighted averages. Paired t-test shows no significant difference (paired t-test is not statistically significant, P value = 0.251>0.05).

NA: not applicable.

When we compared AS events during the different physiological and developmental conditions of growth, starvation and conjugation, we found 172 genes showing stage-specific AS events, meaning that the AS event occurred in only one of the three stages. Most of the stage-specific AS genes were conjugation-specific and IR was the majority class of AS events (Table S6). Only the frequency, not the basic features, of these conjugation-specific IR events appears to be programmed (Table 3, columns E). Thus, they likely reflect physiologically significant stage-specific regulation and not a random consequence splicing machinery weakness or a contribution of massive non-coding transcription that occurs during conjugation in *Tetrahymena* (see Discussion section).

Among the conjugation specific AS genes, protein kinases comprised a major class, with ten protein kinase domain-containing genes undergoing conjugation-specific AS (Table S6). Some of these protein kinases are associated with cell proliferation and cell cycle events, such as the mitogen-activated protein kinase (MAPK) genes and cyclin-dependent kinase 2 (CDK2). In *T. thermophila*, homologs of MAPK gene (TTHERM_00660130) and CDK2 gene (TTHERM_01080590) were both found to undergo AS in conjugation (Table S6). In addition, a potential homolog of human cyclin A (TTHERM_00693080, BlastP E-value: 1e-22) also showed conjugation specific AS (Table S6). These results suggested that AS of these genes may have important functions in conjugation. MAPK genes play important roles in mammalian cell proliferation and differentiation [34]; AS isoforms of MAPK genes show different tissue distribution and distinct physiological responses in humans and plants [35], [36]. CDK2 gene has been reported to be essential in the meiotic cell cycle [37], and was able to form a complex with cyclin A. A CDK2 AS transcript variant has also been found in humans [38], although its function is not clear. Other than the protein kinases, we did not find conjugation-specific AS genes focused on a special pathway, function category or gene family.

## Discussion

This study describes the first deep sequencing analysis of the transcriptome of the ciliated protozoan *Tetrahymena thermophila*, carried out at three major stages of the life cycle: exponential growth, starvation and conjugation. These results provide a fully comprehensive view of the global transcriptome of *T. thermophila*, reveal a previously unsuspected level of alternative splicing, and significantly improve the genome annotation.

### Dynamic ranges of RNA-seq and *Tetrahymena* gene expression

Gene expression data for the two biological duplicate samples showed a very high correlation with one another (R coefficient = 0.924; Fig. 1A), validating the reproducibility of RNA-seq measurements in *Tetrahymena*. Figure 1 also displays the great dynamic range, covering over nearly a million-fold, of RNA-seq measurements. This range brings out the remarkably broad range of gene expression, nearly six orders of magnitude (Figure 1), that this single cell can orchestrate.

A high correlation between RNA-seq and microarray data has been reported in earlier studies [14], [21]. In the case of *Tetrahymena*, RNA-seq shows a greater dynamic range relative to the microarray study of Miao et al. 2009. The difference, graphically displayed by the sigmoid shape of the correlation curves in Figure 1 reflects both greater sensitivity of detection of low gene expression levels as well as greater ability to resolve very high levels of gene expression. These deviations from linearity of the measurement comparison plots contributed to the lower, though still respectable (0.702–0.892), correlation coefficients determined from the data shown in Figure 1.

The high sensitivity of RNA-seq has decreased the number of *T. thermophila* predicted genes not yet confirmed as being expressible – and therefore real – from 22% in the microarray study to 3.8%, even though a much smaller sample of time points was sampled for the same conditions in this RNA-seq study. The percentage may be reduced even further when other conditions are tested, including various stress conditions repeatedly encountered in the natural environment (e.g., heat, hypoxia, heavy metal contamination). Ciliates are free-living unicellular eukaryotes that have surprisingly high numbers of genes (Table 2). Our results mean that the number of real genes in *T. thermophila* is comparable to that in humans and other mammals.

Using RPKM fold changes, genes specifically up-regulated during *T. thermophila* growth, starvation, and conjugation were identified; they showed distinct abundance and functions. Conjugation had the highest number of up-regulated genes, enriched in those involved in DNA− or nucleus-related biological processes. In contrast, growth has about half number of specifically up-regulated genes compared to conjugation, with an overrepresentation of genes involved in metabolic and catabolic processes. While clearly MIC and MAC DNA replication are very active during growth, the overrepresentation of DNA− and nucleus-related processes during conjugation may be related to the much greater complexity and nuclear selectivity of events that occur during this life cycle stage. Conjugation includes three whole-genome sequence comparisons (meiosis and scanning the parental and new MAC genomes for IES sequence), several waves of DNA replication and nuclear division (meiosis and two waves of mitotic division to generate gamete pronuclei and the MAC and MIC anlagen), a wave of meiotic recombination and several types of nuclear differentiation (migratory vs. stationary gamete pronuclei and new MICs vs. MACs), a wave of MAC genome rearrangement in which ∼1/3 of the MIC-derived DNA is excised and destroyed, and the apoptotic destruction of the parental MAC [39].

### Improving *Tetrahymena* gene structural annotation

*De novo* gene annotation is almost exclusively dependent on gene prediction computer programs that detect protein coding regions. However, the lack of extensive *T. thermophila* cDNA libraries or a large EST database limited the quality of the original genome annotation. Our RNA-seq data were used to discover and assemble RNA transcripts from more than 96% of previously predicted genes. In addition, more than 1,000 novel transcribed regions were identified that had been missed by the original de novo gene prediction. Furthermore, because assembled transcripts also yielded comprehensive data on 5′ and 3′ UTRs, our results provide the first estimate of the *T. thermophila* transcriptome size, which at a minimum comprises about 55% of the ∼104 Mb macronuclear genome.

Another major contribution of this RNA-seq study has been to correct the structural annotation for thousands of gene models in *T. thermophila*. While the vast majority of approximately 25,000 *Tetrahymena* genes were originally identified, an important weakness was the incorrect prediction of splice sites. Only 26.8% of the de novo identified gene models were completely confirmed by our RNA-seq data, and more than 50% of the predicted RNA-seq splice sites were inconsistent with those of previous genome annotations. Altogether, over 7,290 predicted gene models were corrected. These corrections allowed us to reassign microarray probes to corrected genes, thus permitting the renormalization and revitalization of the earlier microarray data, which had analyzed a more extensive number of time points for the same physiological/developmental states.

### Alternative splicing is much more frequent in *Tetrahymena* than previously thought

Alternative splicing is an important mechanism for enhancement of transcriptome and proteome diversity. In metazoa and plants, generally more than 30% of genes show AS (Table 2). A major value of RNA-seq is that it provides a cost-effective, genome-wide means to discover alternatively-splicing. Our study identified 1,474 AS events in *T. thermophila*, distributed over 5.2% of *Tetrahymena* genes. This AS percentage represents a two order of magnitude increase over previous EST-based estimates of AS in *Tetrahymena* [10]. This percentage is likely still an underestimate, as stringent detection criteria were used, and rare AS isoforms or isoforms of poorly expressed genes may have been missed due to sequence coverage limitation.

To our knowledge, *Tetrahymena* has the highest number and percentage of genes showing AS reported in a unicellular eukaryote (Table 2). Although higher, the percentages are comparable to those shown in some unicellular eukaryotes, e.g., *Plasmodium*, *Chlamydomonas*, *Cryptococcus*; In other unicellular eukaryotes (e.g. *S. cerevisiae* and *Paramecium tetraurelia*), the occurrence of AS has been severely reduced by an order of magnitude relative to *Tetrahymena*. In *S. cerevisiae*, more than 95% of genes are intronless, and few cases of AS were identified [40]; only one case of AS was found in another yeast, *Candida albicans* [41]. In the ciliate *Paramecium tetraurelia* 0.9% of the genes show AS [42]. Other than the higher incidence of AS in metazoa, Table 2 fails to show any meaningful correlations between AS gene fraction and either phylogenetic relationship or the average number of introns per gene, with the caveat that different methods and stringencies of AS detection were used in different studies. Many surprises undoubtedly lie in store as the enormous diversity of unicellular eukaryote transcriptomes are increasingly being explored by RNA-seq.

A specific source of concern about the IR class of AS events was that it could be spuriously enriched for unprocessed non-coding transcripts. Among the three stages of the life cycle, conjugating cells show the absolute majority of IR events and the absolute majority of stage-specific IR events (Table S6). Coincidentally, during conjugation (but not growth or starvation) in *Tetrahymena* cells there is massive, genome-wide promiscuous transcription of non-coding (NC) RNAs at three different stages [43]. These NC transcripts are likely to contain internal adenosine tracts (polyA), which are very common in the highly A+T-rich (85–90%) intergenic or intron regions of *Tetrahymena*. A fraction of those NC transcripts thus could theoretically have been captured by polyT beads during mRNA purification for cDNA sequencing library construction. If such non-coding transcripts fail to undergo splicing they would result in spurious cases of AS by intron retention.

Although we have found no reports about whether or not such non-coding transcripts undergo splicing events, there are reasons to suggest that they likely do not. They function to convey and interpret information related to the accurate identification and somatic excision of non-coding DNA sequence; thus their genomic-sequence-dependent function could be compromised if the transcripts were spliced. Furthermore, if they were subject to splicing, the waves of non-coding transcription are so massive that the splicing machinery might be overwhelmed; if so, a random selection of introns could miss being excised or certain transcripts could escape splicing altogether. Our analyses of frequency and distribution of IR events (Table 3, columns D) show an extremely low fraction of IR events that could be attributed to transcripts that are immune to splicing or that undergo random IR events. Furthermore, no significant decrease in the specificity with which IR occurs was observed in conjugating cells (Table 3, columns E) in comparison to growth and starved cells, when no massive non-coding transcription occurs. Thus it seems most likely that NC transcripts make at most a negligible contribution of spurious events to the IR class of AS events and that the vast majority of apparent IR events are indeed real, programmed events occurring in legitimate gene transcripts. These considerations apply equally well to the conjugation-specific IR cases of AS events that we report in the Results section.

### What functional significance could AS have in the Tetrahymena unicell?

Cell differentiation in multicellular eukaryotes creates different and sometimes conflicting demands in level of gene expression in different cell types. This has demanded the evolution of families of proteins that are closely related but may have slightly different function and may be differently regulated. The combinatorial potential of AS may have been able to provide a more rapidly attainable and less costly way to generate what are in effect closely related paralogs capable of satisfying those demands.

The above considerations are applicable to the subcellular level of a complex unicellular organism, as diversification of gene function is also observed within unicells and undoubtedly preceded the evolution of multicellularity. For example, this study has shown that the gene TTHERM_00558310, a heavy chain subunit of non-axonemal dynein complex 2 (DYH2), undergoes alternative 5′ splice-site selection and intron retention types of alternative splicing. This gene is not only required for the maintenance of the cortical microtubule cytoskeleton [44], but also important in the regulation of ciliary length [45]. Our discovery of 172 stage-specific AS genes (especially the 130 conjugation-specific cases) in *Tetrahymena* raises the possibility that AS isoforms have, in some cases, evolved to carry out distinct but related functions. The widespread occurrence of AS in *Tetrahymena* uncovered by this study thus provides an alternative way to explain the functional differentiation of many gene products. In principle, this alternative is readily testable in *T. thermophila* where DNA-mediated transformation occurs by exact homologous recombination [46] allowing replacement of a wild type gene by an engineered, AS isoform-specific version of the gene.

Additionally, in a variety of eukaryotes, AS, in combination with the degradation of AS isoforms containing in-frame premature stop codons (nonsense-mediated mRNA decay) [47], plays an essential role in a posttranscriptional mechanism of gene regulation. We have documented the occurrence of this AS-enabled mechanism, known as “regulated unproductive splicing and translation” (RUST), in *T. thermophila* (Xiong et al, under review).

There are many aspects of the AS mechanism that remain unanswered, including its possible relationship to RNAi and epigenetics [48]. *Tetrahymena* has been an excellent model organism for the study of RNA biology. Examples are the co-discovery of catalytic RNA [4], of telomerase RNA as the template for telomere elongation [3], the role of RNAi in programmed genome rearrangement [6], and the vast abundance and diversity of Argonaute protein-RNAi species used in a single cell [49]. *Tetrahymena* may well be an excellent model organism in which to investigate the mechanisms involved in alternative splicing of eukaryotes.

### Conclusions

This study described the first deep RNA sequencing analysis of the *T. thermophila* transcriptome at the three major stages of its life cycle. It significantly improved the *T. thermophila* gene annotation and provided comprehensive understanding of its global transcriptome. Significantly, our study identified 1,474 AS events distributed over 5.2% of *T. thermophila* genes. This percentage represents a two order of magnitude increase over previous EST-based estimates and, to our knowledge, the highest percentage of genes showing AS reported in a unicellular eukaryote. *Tetrahymena* thus becomes an excellent model organism in which to investigate the mechanisms involved in alternative splicing of eukaryotes.

## Methods

### Cells, Cell culture and RNA samples preparation

Two cell lines of *Tetrahymena thermophila*, SB4217 and SB4220 (mating types V and VI respectively) have inbred strain B genetic background, as does the cell line SB210 (also mating type VI) which was used for the MAC genome sequencing and for earlier analyses of gene expression profiling by microarrays. All these strains are available at *Tetrahymena* Stock Center (http://tetrahymena.vet.cornell.edu/index.php)

Cell culture, starvation and mating conditions as well as total RNA extraction conditions were as in the previous microarray analysis (http://tged.ihb.ac.cn/sample.aspx in TGED, [50]). For growth condition, mating type VI cells were cultured in 1XSPP at 30°C and 150 rpm shaking, mid-log exponential growth cells (∼350K cells/ml) were harvested; For starvation, mating type V and VI cells were separately starved at 200 K cells/ml in 10 mM Tris without shaking, Samples were collected at 3 (mating type V and VI) and 15 (only mating type VI) hours after starvation. For conjugation, mating type V and VI cells that had been starved for 18 hours were resuspended in 10 mM Tris (pH 7.5) at 200 K cells/ml, mixed in equal volumes, and samples were collected at 2 and 8 hours after mixing. Total RNA was extracted using the RNeasy Protect Cell Mini Kit (Qiagen, Valencia, CA) according to protocol in TGED. Total RNA concentrations were determined using the e-Spect ES-2 spectrophotometer (MALCOM, Japan) and RNA integrity was verified using gel electrophoresis in denaturing 1.2% Agarose.

### Library preparation and sequencing

Poly-A mRNAs were isolated using Dynal magnetic beads (Invitrogen) and fragmented by heating at 94°C. First strand cDNAs were synthesized with reverse transcriptase and random hexamer primers, and then the second strands were synthesized with DNA polymerase and random hexamer primers. Double strand cDNAs were end-repaired and a single adenosine moiety was added. Illumina adapters were ligated and gel-electrophoresis was used to select DNA fragments between 200–250 bp in size. All the enzymes used above are included in the kit from Illumina (Cat. No. RS-100-0801). Libraries were PCR-amplified using Phusion polymerase. Sequencing libraries were denatured with sodium hydroxide and diluted in hybridization buffer for loading onto a single lane of an Illumina GA flow cell. Cluster formation, primer hybridization and pair-end sequencing were performed using proprietary reagents according to manufacturer-recommended protocols (https://icom.illumina.com/).

A total of six RNA samples at five time points of *T. thermophila* life cycle were sequenced, including mid-log exponential growth (∼3.5×10^5^ cells/ml, referred to as G-m) mating type VI cells, starvation for 3 hours (referred to as S-3) of separate mating type V and VI cells, starvation for 15 hours (referred to as S-15) of mating type VI cells, conjugating cells at 2 hours (referred to as C-2) and conjugating cells at 8 hours (referred to as C-8) after mixing cells of mating type V and VI.

### Data processing

Sequence image analysis was performed using the Firecrest, Bustard and GERALD programs (Illumina). Low quality bases (Q<5) at the ends of the reads were trimmed. The current release of the *T. thermophila* reference macronuclear genome consists of 1148 scaffolds [10] (ftp://ftp.tigr.org/pub/data/Eukaryotic_Projects/t_thermophila/annotation_dbs/final_release_oct2008/). The TopHat program [51] (version 1.1.4, parameters: -i 10, -I 10000, –coverage-search, –microexon-search, -m 2) was used to map the sequenced reads to the reference genome and to find those that spanned exon-exon junctions. The NUCmer program (parameters: -c 25, -l 15, -g 10000) in the MUMmer 3.0 package [52] and a custom script were used to find reads that failed to map in the previous step and that possibly spanned two or more exon-exon junctions. At least 8 bp were required to match on either side of the junction. Mapped reads were allowed up to two mismatches and only canonical 5′ GT and 3′ AG terminal intron dinucleotides were accepted for reads that spanned exon-exon junctions. Transcripts were assembled using the Cufflinks software (version 0.9.2, parameters: -I 10000, –min-intron-length 10) [23], and were compared to the predicted gene models using cuffcompare software [23] and custom scripts. Putative ORFs of assembled transcripts were found using the GetOrf program in the EMBOSS package [53]. The RNA-seq data used in this study have been deposited in the NCBI Gene Expression Omnibus (http://www.ncbi.nlm.nih.gov/geo) under accession no. GSE27971 including GSM692081, GSM692082, GSM692083, GSM692084, GSM692085 and GSM692086. Corrected gene models will be made public through a *Tetrahymena* Functional Genomics Database (TetraFGD) (http://tfgd.ihb.ac.cn/).

### Gene expression analysis

For RNA-seq data, the gene expression level was measured as the numbers of reads per kilobase of exon region in a gene per million mapped reads (RPKM) [17]. The number of reads mapped to each transcript was determined using the HTSeq software (http://www-huber.embl.de/users/anders/HTSeq/doc/overview.html). For comparison to the microarray data, the RPKM values for each confirmed gene model were used. The microarray data and probe information were retrieved from the TGED website and the Gene Expression Omnibus (GEO) database. The Pearson correlation coefficients were calculated using SPSS software (version 13). Microarray expression levels were re-normalized using the median value of the normalized expression value for probes located in the corrected gene models. To identify genes that were up-regulated at one stage relative to another, two criteria had to be met. 1) the maximum RPKM expression level had to be greater than 5, and 2) there had to be at least a five-fold change between the expression levels of the each two stages comparison.

### Function enrichment analysis

Function categories of genes were annotated using the gene ontology (GO) information. All genes in *T. thermophila* were first aligned by BlastP to NCBI non redundant database, and then Blast2GO [54] was used to annotate the sequences with GO terms. For each gene set, GO enrichment analysis was also carried out using BinGO [55]. Bonferroni correction was used to control the false positive rate. If a GO term in a test gene set showed a corrected p value less than 0.01 compared with the reference set (all the GO annotated genes in *T. thermophila*), the GO term (function) was determined to be significantly overrepresented in test gene set.

### Alternative splicing detection

Canonical GT-AG splice sites were used to identify sites with alternative splicing. We classified alternative splicing events into four basic types: cassette-exon inclusion or skipping, alternative 5′ splice-site selection, alternative 3′ splice-site selection and intron retention. Stringent criteria were used for identifying alternative splicing events. For intron retention, we required that the retained region have at least ten-fold base coverage for each nucleotide position. Every accepted exon-exon junction had to be supported by at least two non-redundant reads spanning the junction.

### Measurement of average AS intron length

The average length of AS intron isoforms in a given gene was calculated as follows for each AS type: average of the two AS introns lengths for 5′ and 3′ selection AS, average of the three introns lengths for exon-skipping AS and simply the intron length for intron retention AS.

## Supporting Information

Figure S1 **The length distribution of the UTR of confirmed gene models.** Number of genes with UTRs of the indicated length vs UTR length (bp). Red, 5′ UTR; Green, 3′ UTR.
(TIF)

Figure S2 **Overrepresented functions among genes which are up-regulated during growth and conjugation.** A, growth; B, conjugation. Each circle represents a GO term, arrows indicate pairs of GO terms with a parent-child relationship. Colored circles are statistically significantly overrepresented GO terms (functions); the deeper of the color, the smaller the corrected p-value (more significant).
(TIF)

Figure S3 **Four basic types of alternative splicing in** ***T. thermophila*** **.** A, cassette-exon inclusion or skipping; B, alternative 5′ splice-site selection; C, alternative 3′ splice-site selection; D, intron retention. Only reads supporting alternative splices are schematically shown.
(TIF)

Table S1 **Classification of 955 previously annotated genes that failed to be detected by RNA-seq.**
(DOC)

Table S2 **955 previously annotated genes that failed to be confirmed by RNA-seq.**
(DOC)

Table S3 **Comparisons of intron base compositions.**
(DOC)

Table S4 **217 novel transcribed regions annotated in previous genome annotation but lost in current genome annotation.**
(DOC)

Table S5 **The 745 novel transcribed regions giving blast hits with expected values less than 1e-3.**
(DOC)

Table S6 **Genes with state-specific alternative splicing.**
(DOC)

Text S1 **Additional materials for novel and missing genes in the RNA-seq data.** Section 1: 955 genes in the 2008 prediction that RNA-seq failed to detect; Section 2: 1237 novel transcribed regions identified by RNA-seq.
(DOC)
