# Supplementary figures and images for: Transcriptome Analysis of the Model Protozoan, Tetrahymena thermophila, Using Deep RNA Sequencing
Source: PLoS One. 2012 Feb 7;7(2):e30630. doi: 10.1371/journal.pone.0030630 (PMC3274533; doi:10.1371/journal.pone.0030630)

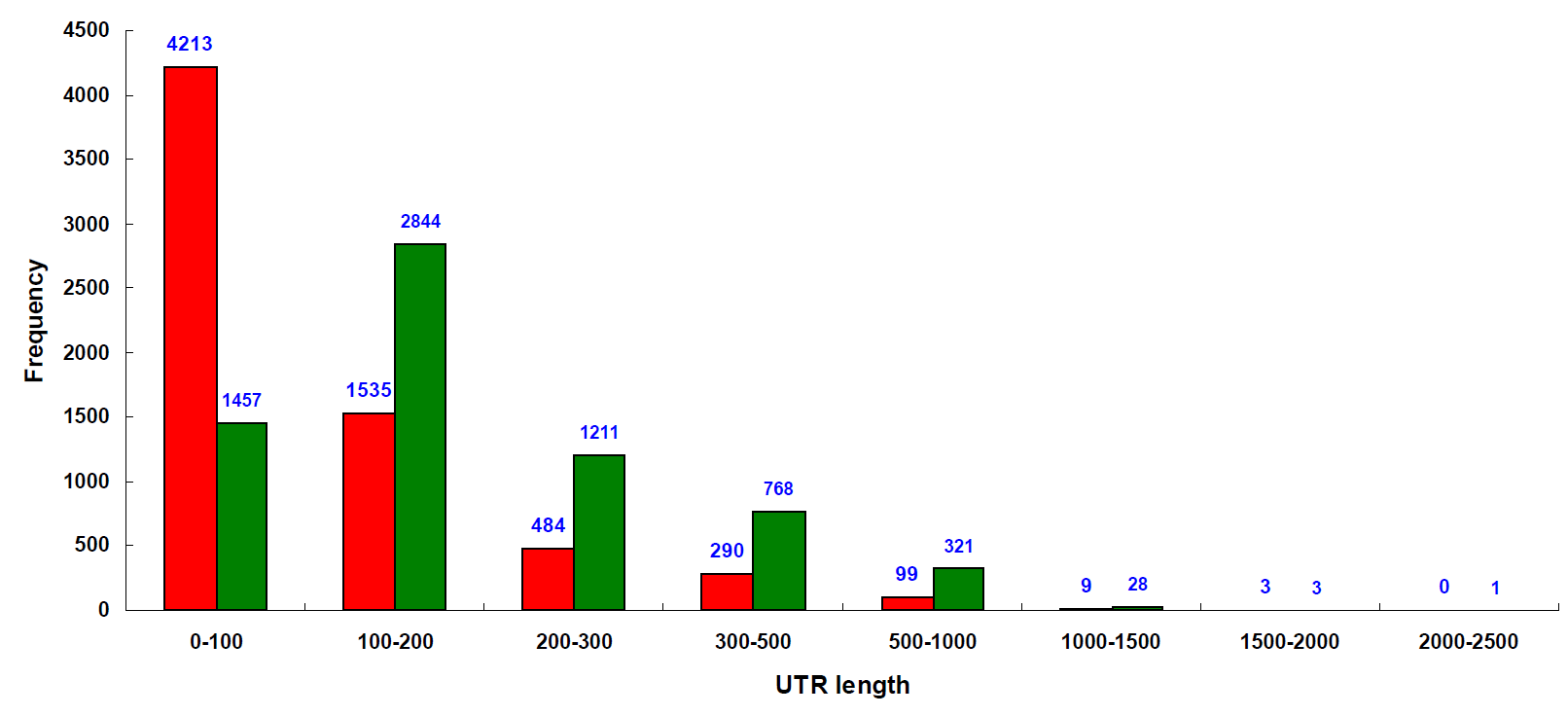

Supplement: Figure S1 — The length distribution of the UTR of confirmed gene models. Number of genes with UTRs of the indicated length vs UTR length (bp). Red, 5′ UTR; Green, 3′ UTR. (TIF) [file pone.0030630.s001.tif]

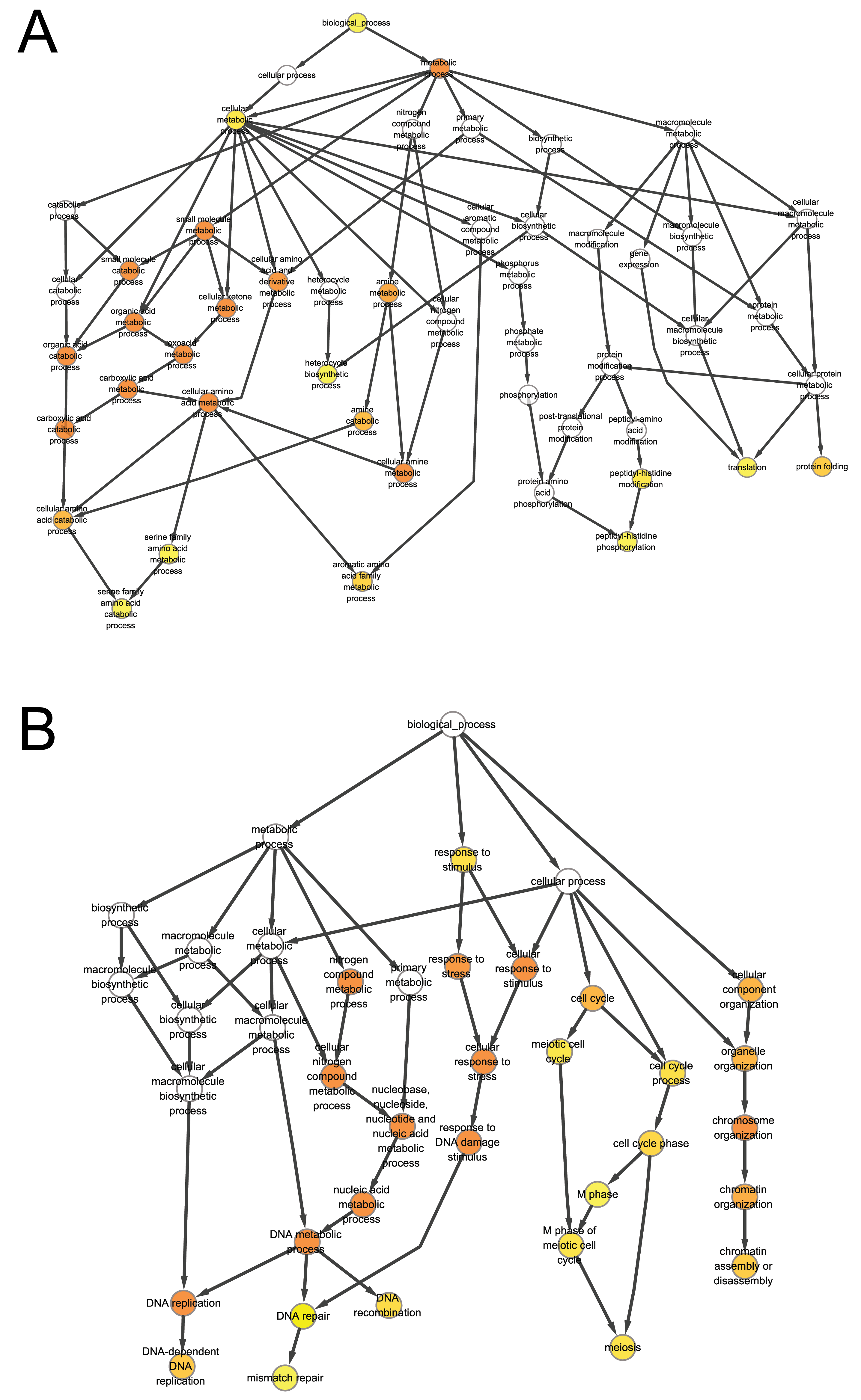

Supplement: Figure S2 — Overrepresented functions among genes which are up-regulated during growth and conjugation. A, growth; B, conjugation. Each circle represents a GO term, arrows indicate pairs of GO terms with a parent-child relationship. Colored circles are statistically significantly overrepresented GO terms (functions); the deeper of the color, the smaller the corrected p-value (more significant). (TIF) [file pone.0030630.s002.tif]

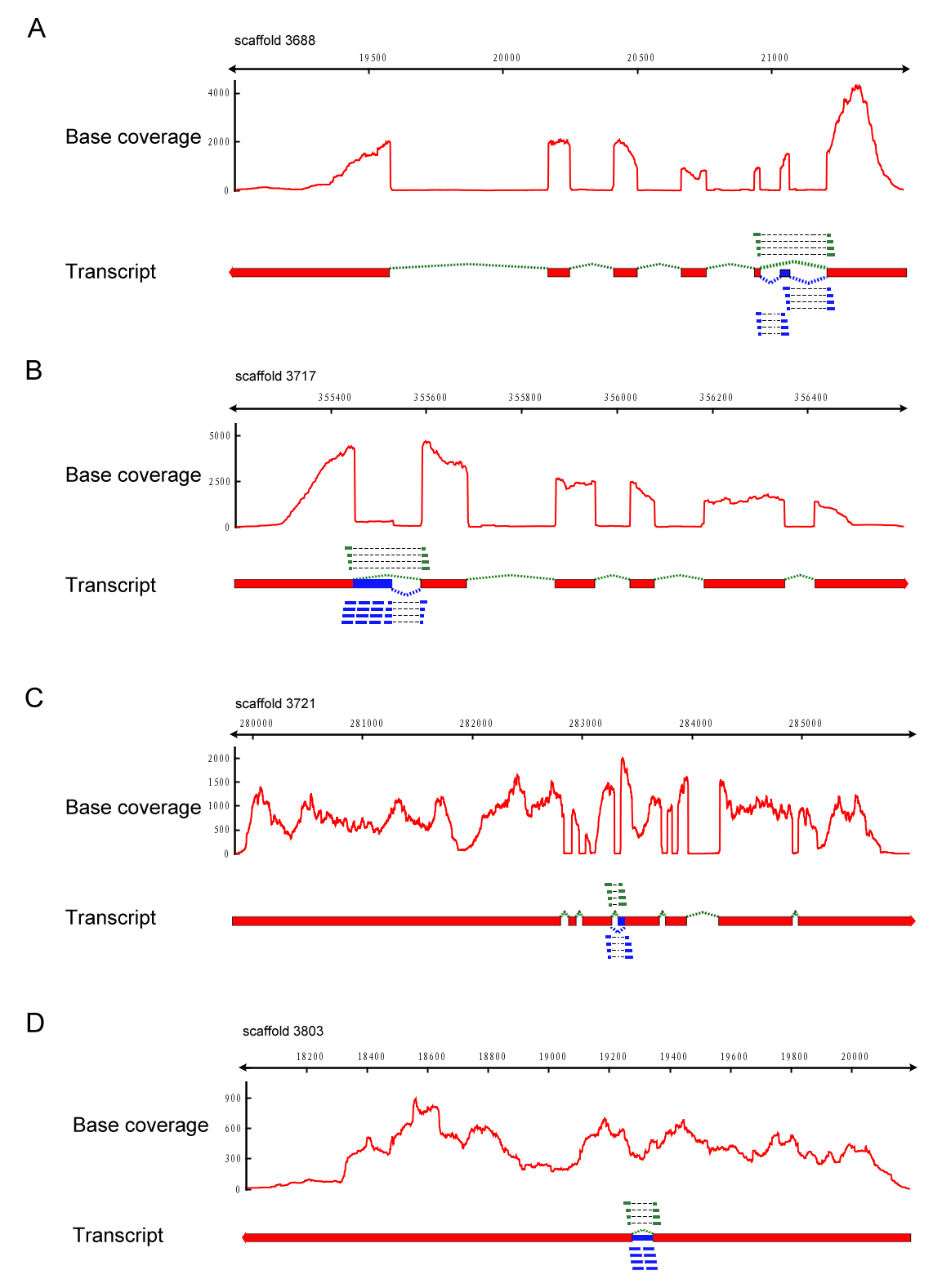

Supplement: Figure S3 — Four basic types of alternative splicing in T. thermophila . A, cassette-exon inclusion or skipping; B, alternative 5′ splice-site selection; C, alternative 3′ splice-site selection; D, intron retention. Only reads supporting alternative splices are schematically shown. (TIF) [file pone.0030630.s003.tif]
