# Supplementary material for: Transcriptome Analysis of the Model Protozoan, Tetrahymena thermophila, Using Deep RNA Sequencing
Source: PLoS One. 2012 Feb 7;7(2):e30630. doi: 10.1371/journal.pone.0030630 (PMC3274533; doi:10.1371/journal.pone.0030630)
Supplement: Table S1 — Classification of 955 previously annotated genes that failed to be detected by RNA-seq. (DOC) [file pone.0030630.s004.doc]

**Table S1. Classification of 955 previously annotated genes that failed to be detected by RNA-seq.**

| **Class** | **Description** | **No of gene models** |
| --- | --- | --- |
| 1 | Microarray gene expression exceeded background level | 140* |
| 2 | Microarray gene expression below background level but gene model gave Blast hits to genes in other organisms | 60** |
| 3 | Microarray gene expression below background level and gene model gave no blast hits to other organisms | 684*** |
| 4 | Gene models without designed microarray probes | 71**** |

* 80 gene models had expression levels > 4X background level. Five gene models had expression levels > 400X background level.

** 38 gene models gave non-*Tetrahymena* blast hits with expected value ranging from 1E-05 to 4E-88.

*** 4 of these gene models had Pfam matches.

**** Mainly too small to give unique, acceptable probes.
