# Supplementary material for: Transcriptome Analysis of the Model Protozoan, Tetrahymena thermophila, Using Deep RNA Sequencing
Source: PLoS One. 2012 Feb 7;7(2):e30630. doi: 10.1371/journal.pone.0030630 (PMC3274533; doi:10.1371/journal.pone.0030630)
Supplement: Table S2 — 955 previously annotated genes that failed to be confirmed by RNA-seq. (DOC) [file pone.0030630.s005.doc]

**Table S2. 955 previously annotated genes that failed to be confirmed by RNA-seq.**

| **Gene Model ID** | **Annotation** | **Aa length** | **non Tetrahymena BLASTP hit (E-value<1E-2)** | **Pfam domain** | **Max expression level in 20 microarrays (Background = 99)** |
| --- | --- | --- | --- | --- | --- |
| **Class 1. Microarray gene expression exceeded background** | | | |  |  |
| TTHERM_01853030 | hypothetical protein | 56 | No hits in other organism | NA | 43034.76 |
| TTHERM_02348010 | hypothetical protein | 75 | No hits in other organism | NA | 42242.44 |
| TTHERM_01748600 | hypothetical protein | 41 | No hits in other organism | NA | 42164.38 |
| TTHERM_01701340 | hypothetical protein | 76 | No hits in other organism | NA | 41446.85 |
| TTHERM_02315930 | hypothetical protein | 80 | No hits in other organism | NA | 39897.8 |
| TTHERM_02315920 | hypothetical protein | 80 | No hits in other organism | NA | 38918.08 |
| TTHERM_00637860 | hypothetical protein | 81 | No hits in other organism | NA | 37716.96 |
| TTHERM_02636280 | hypothetical protein | 82 | No hits in other organism | NA | 34601.53 |
| TTHERM_02450140 | hypothetical protein | 80 | No hits in other organism | NA | 34193.28 |
| TTHERM_00881360 | hypothetical protein | 42 | No hits in other organism | NA | 32951.74 |
| TTHERM_00869590 | hypothetical protein | 103 | No hits in other organism | NA | 32777.58 |
| TTHERM_01184190 | hypothetical protein | 45 | No hits in other organism | NA | 31639.4 |
| TTHERM_01524430 | hypothetical protein | 83 | No hits in other organism | NA | 29056.93 |
| TTHERM_01687280 | hypothetical protein | 44 | No hits in other organism | NA | 28758.16 |
| TTHERM_02318940 | hypothetical protein | 117 | No hits in other organism | NA | 28540.95 |
| TTHERM_01234330 | hypothetical protein | 65 | No hits in other organism | NA | 23613.2 |
| TTHERM_01580330 | hypothetical protein | 80 | No hits in other organism | NA | 23098.51 |
| TTHERM_02631270 | hypothetical protein | 40 | No hits in other organism | NA | 22528.2 |
| TTHERM_02379080 | hypothetical protein | 95 | No hits in other organism | NA | 21911.53 |
| TTHERM_01054410 | hypothetical protein | 82 | No hits in other organism | NA | 20013.39 |
| TTHERM_00713560 | hypothetical protein | 62 | No hits in other organism | NA | 18983.93 |
| TTHERM_01283700 | hypothetical protein | 62 | No hits in other organism | NA | 17153.03 |
| TTHERM_01054120 | hypothetical protein | 51 | No hits in other organism | NA | 17011.62 |
| TTHERM_02589230 | CARD15-like protein, putative | 132 | No hits in other organism | NA | 13631.26 |
| TTHERM_00127240 | hypothetical protein | 63 | No hits in other organism | NA | 12885.69 |
| TTHERM_00161830 | conserved hypothetical protein | 359 | putative splicing factor [Polysphondylium pallidum PN500] E-VALUE:3.00E-13 | PF06003.5 | 11793.18 |
| TTHERM_01604660 | hypothetical protein | 82 | No hits in other organism | NA | 10826.9 |
| TTHERM_01470390 | hypothetical protein | 68 | No hits in other organism | NA | 9407.42 |
| TTHERM_00676900 | hypothetical protein | 59 | No hits in other organism | NA | 9178.08 |
| TTHERM_02546190 | hypothetical protein | 81 | No hits in other organism | NA | 8932.69 |
| TTHERM_01228970 | hypothetical protein | 56 | No hits in other organism | NA | 8662.92 |
| TTHERM_01218290 | hypothetical protein | 73 | No hits in other organism | NA | 8533.37 |
| TTHERM_01595610 | hypothetical protein | 80 | No hits in other organism | NA | 8407.1 |
| TTHERM_02558200 | hypothetical protein | 49 | No hits in other organism | NA | 7927.93 |
| TTHERM_01301790 | hypothetical protein | 49 | No hits in other organism | NA | 7343.09 |
| TTHERM_02050520 | hypothetical protein | 82 | No hits in other organism | NA | 6840.91 |
| TTHERM_01218280 | hypothetical protein | 73 | No hits in other organism | NA | 6237.08 |
| TTHERM_01356300 | hypothetical protein | 319 | No hits in other organism | NA | 5995.13 |
| TTHERM_00903930 | hypothetical protein | 40 | No hits in other organism | NA | 5965.79 |
| TTHERM_00835560 | hypothetical protein | 46 | No hits in other organism | NA | 5582.93 |
| TTHERM_00214690 | hypothetical protein | 43 | No hits in other organism | NA | 4900.6 |
| TTHERM_01146050 | hypothetical protein | 57 | No hits in other organism | NA | 4358.64 |
| TTHERM_01148230 | hypothetical protein | 42 | No hits in other organism | NA | 4186.02 |
| TTHERM_01463210 | hypothetical protein | 40 | No hits in other organism | NA | 3846.33 |
| TTHERM_00053910 | hypothetical protein | 42 | No hits in other organism | NA | 3662.18 |
| TTHERM_01332050 | hypothetical protein | 140 | No hits in other organism | NA | 3643.03 |
| TTHERM_00465070 | hypothetical protein | 60 | No hits in other organism | NA | 3464.94 |
| TTHERM_00866550 | hypothetical protein | 73 | No hits in other organism | NA | 3191.97 |
| TTHERM_00725830 | hypothetical protein | 82 | No hits in other organism | NA | 3148.24 |
| TTHERM_01188430 | hypothetical protein | 29 | No hits in other organism | NA | 3021.56 |
| TTHERM_01167350 | hypothetical protein | 72 | No hits in other organism | NA | 2972.82 |
| TTHERM_01782760 | hypothetical protein | 61 | No hits in other organism | NA | 2691.01 |
| TTHERM_00429810 | hypothetical protein | 45 | No hits in other organism | NA | 2619.7 |
| TTHERM_00265120 | hypothetical protein | 106 | PREDICTED: ATP-binding cassette, sub-family C (CFTR/MRP), member 4 [Equus caballus] E-VALUE:9.00E-21 | PF00005.20 | 2384.38 |
| TTHERM_02596230 | hypothetical protein | 287 | hypothetical protein [Paramecium tetraurelia strain d4-2] E-VALUE:5.00E-25 | PF02010.8 | 2188.72 |
| TTHERM_00628660 | hypothetical protein | 55 | No hits in other organism | NA | 2104.96 |
| TTHERM_01447980 | hypothetical protein | 54 | No hits in other organism | NA | 2050.61 |
| TTHERM_00376190 | hypothetical protein | 230 | No hits in other organism | NA | 2023.47 |
| TTHERM_00835160 | hypothetical protein | 74 | No hits in other organism | NA | 2009.67 |
| TTHERM_00808000 | hypothetical protein | 226 | No hits in other organism | NA | 1943.82 |
| TTHERM_00268170 | hypothetical protein | 250 | predicted protein [Nematostella vectensis] E-VALUE:4.00E-05 | NA | 1734.46 |
| TTHERM_00960840 | hypothetical protein | 98 | No hits in other organism | NA | 1668.47 |
| TTHERM_00152200 | hypothetical protein | 61 | No hits in other organism | NA | 1594.65 |
| TTHERM_00561480 | hypothetical protein | 284 | conserved unknown protein [Ectocarpus siliculosus] E-VALUE:3.00E-04 | NA | 1182.86 |
| TTHERM_00277560 | hypothetical protein | 256 | No hits in other organism | NA | 989.67 |
| TTHERM_01362570 | hypothetical protein | 71 | No hits in other organism | NA | 943.03 |
| TTHERM_02258850 | hypothetical protein | 83 | No hits in other organism | NA | 907.08 |
| TTHERM_01372800 | hypothetical protein | 64 | No hits in other organism | NA | 864.42 |
| TTHERM_00427580 | hypothetical protein | 177 | No hits in other organism | NA | 852.78 |
| TTHERM_01411030 | hypothetical protein | 181 | No hits in other organism | NA | 826 |
| TTHERM_01865050 | hypothetical protein | 83 | No hits in other organism | NA | 802.75 |
| TTHERM_01221450 | hypothetical protein | 65 | No hits in other organism | NA | 756.92 |
| TTHERM_00289580 | hypothetical protein | 94 | No hits in other organism | NA | 744.71 |
| TTHERM_00881350 | hypothetical protein | 82 | vivapain-2 [Plasmodium vivax] E-VALUE:0.001 | PF08246.5 | 737.15 |
| TTHERM_02583230 | hypothetical protein | 168 | No hits in other organism | NA | 673.32 |
| TTHERM_00383570 | hypothetical protein | 52 | No hits in other organism | NA | 658.77 |
| TTHERM_00411670 | hypothetical protein | 43 | No hits in other organism | NA | 580.11 |
| TTHERM_00158410 | hypothetical protein | 41 | No hits in other organism | NA | 476.34 |
| TTHERM_01154650 | hypothetical protein | 55 | No hits in other organism | NA | 414.65 |
| TTHERM_00934410 | GIY-YIG catalytic domain containing protein | 206 | No hits in other organism | PF01541.17 | 402.13 |
| TTHERM_01150360 | hypothetical protein | 447 | No hits in other organism | NA | 387.27 |
| TTHERM_00446230 | hypothetical protein | 41 | No hits in other organism | NA | 386.93 |
| TTHERM_00923060 | hypothetical protein | 49 | No hits in other organism | NA | 383.63 |
| TTHERM_00935520 | hypothetical protein | 120 | No hits in other organism | NA | 383.26 |
| TTHERM_00283590 | hypothetical protein | 152 | No hits in other organism | NA | 357.46 |
| TTHERM_00104930 | hypothetical protein | 63 | No hits in other organism | NA | 350.44 |
| TTHERM_01787780 | hypothetical protein | 79 | No hits in other organism | NA | 344.02 |
| TTHERM_00595410 | hypothetical protein | 51 | No hits in other organism | NA | 324.51 |
| TTHERM_00156720 | hypothetical protein | 47 | No hits in other organism | NA | 320.56 |
| TTHERM_00115470 | hypothetical protein | 49 | No hits in other organism | NA | 315.26 |
| TTHERM_00330030 | hypothetical protein | 65 | No hits in other organism | NA | 309.22 |
| TTHERM_00491120 | hypothetical protein | 53 | No hits in other organism | NA | 297.97 |
| TTHERM_01326830 | hypothetical protein | 65 | No hits in other organism | NA | 289.74 |
| TTHERM_00894490 | hypothetical protein | 50 | No hits in other organism | NA | 286.88 |
| TTHERM_00090280 | hypothetical protein | 112 | No hits in other organism | NA | 267.94 |
| TTHERM_00488380 | hypothetical protein | 235 | No hits in other organism | PF06873.4 | 255.3 |
| TTHERM_00372640 | hypothetical protein | 202 | ATP-binding cassette transporter, subfamily C, member 4, SmABCC4 [Selaginella moellendorffii] E-VALUE:2.00E-25 | NA | 246.93 |
| TTHERM_00343430 | hypothetical protein | 55 | No hits in other organism | NA | 243.1 |
| TTHERM_01433600 | hypothetical protein | 52 | No hits in other organism | NA | 241.6 |
| TTHERM_00590350 | hypothetical protein | 50 | No hits in other organism | NA | 237.53 |
| TTHERM_00283040 | hypothetical protein | 62 | No hits in other organism | NA | 232.06 |
| TTHERM_00874820 | hypothetical protein | 65 | No hits in other organism | NA | 227.62 |
| TTHERM_01311300 | hypothetical protein | 82 | No hits in other organism | NA | 226.6 |
| TTHERM_00633300 | hypothetical protein | 224 | phosphorylase kinase gamma subunit, putative [Ixodes scapularis] E-VALUE:9.00E-10 | PF00069.18 | 225.3 |
| TTHERM_01381980 | hypothetical protein | 40 | No hits in other organism | NA | 216.12 |
| TTHERM_00237400 | hypothetical protein | 42 | No hits in other organism | NA | 205.73 |
| TTHERM_00848080 | hypothetical protein | 111 | No hits in other organism | NA | 195.41 |
| TTHERM_00301970 | hypothetical protein | 132 | No hits in other organism | NA | 191.31 |
| TTHERM_00257020 | hypothetical protein | 409 | No hits in other organism | NA | 184.85 |
| TTHERM_00773570 | hypothetical protein | 58 | No hits in other organism | NA | 183.8 |
| TTHERM_01138310 | hypothetical protein | 46 | No hits in other organism | NA | 182.11 |
| TTHERM_01087930 | hypothetical protein | 51 | No hits in other organism | NA | 174.61 |
| TTHERM_00997460 | hypothetical protein | 42 | No hits in other organism | NA | 168.54 |
| TTHERM_00129180 | hypothetical protein | 99 | No hits in other organism | NA | 168.12 |
| TTHERM_00697180 | hypothetical protein | 83 | No hits in other organism | NA | 168.11 |
| TTHERM_01639960 | Protein kinase domain containing protein | 165 | Protein kinase domain containing protein, expressed [Oryza sativa Japonica Group] E-VALUE:2.00E-20 | PF00069.18 | 161.46 |
| TTHERM_02564210 | hypothetical protein | 133 | No hits in other organism | NA | 160.2 |
| TTHERM_00129090 | hypothetical protein | 149 | No hits in other organism | NA | 159.48 |
| TTHERM_00741690 | hypothetical protein | 71 | No hits in other organism | NA | 153.62 |
| TTHERM_00058980 | hypothetical protein | 47 | No hits in other organism | NA | 151.43 |
| TTHERM_01159930 | hypothetical protein | 91 | No hits in other organism | NA | 150.52 |
| TTHERM_01978370 | hypothetical protein | 79 | No hits in other organism | NA | 142.15 |
| TTHERM_01413080 | hypothetical protein | 172 | No hits in other organism | NA | 140 |
| TTHERM_00007490 | J immobilization antigen, putative | 118 | No hits in other organism | NA | 138.47 |
| TTHERM_01018350 | hypothetical protein | 79 | No hits in other organism | NA | 136.76 |
| TTHERM_00030150 | hypothetical protein | 103 | DNA repair helicase family protein [Cryptosporidium muris RN66] E-VALUE:3.00E-08 | NA | 131.81 |
| TTHERM_00418450 | hypothetical protein | 50 | No hits in other organism | NA | 130.86 |
| TTHERM_00006490 | conserved hypothetical protein | 140 | No hits in other organism | NA | 126.29 |
| TTHERM_00429700 | hypothetical protein | 271 | conserved Plasmodium protein, unknown function [Plasmodium falciparum 3D7] E-VALUE:9.00E-04 | NA | 125.89 |
| TTHERM_01284790 | hypothetical protein | 93 | conserved Plasmodium protein, unknown function [Plasmodium falciparum 3D7] E-VALUE:0.001 | NA | 124.65 |
| TTHERM_01068250 | hypothetical protein | 51 | No hits in other organism | NA | 121.74 |
| TTHERM_00854420 | hypothetical protein | 96 | No hits in other organism | NA | 121.65 |
| TTHERM_00703730 | hypothetical protein | 45 | No hits in other organism | NA | 118.77 |
| TTHERM_00870590 | hypothetical protein | 41 | No hits in other organism | NA | 115.98 |
| TTHERM_00862760 | hypothetical protein | 51 | No hits in other organism | NA | 113.55 |
| TTHERM_00237410 | hypothetical protein | 509 | RSK-pNinety (RSK-p90 kinase) homolog family member (rskn-1) [Caenorhabditis elegans] E-VALUE:5.00E-38 | PF00069.18 | 109.53 |
| TTHERM_00560120 | hypothetical protein | 298 | No hits in other organism | NA | 107.48 |
| TTHERM_01100460 | hypothetical protein | 49 | No hits in other organism | NA | 107.27 |
| TTHERM_01716400 | hypothetical protein | 144 | flagellar autotomy protein [Micromonas sp. RCC299] E-VALUE:2.00E-05 | NA | 105.29 |
| TTHERM_00310620 | hypothetical protein | 45 | No hits in other organism | NA | 102.5 |
|  |  |  |  |  |  |
| **Class 2. Gene model gave Blast hits to genes in other organisms** | | | |  |  |
| TTHERM_00326990 | ZIP Zinc transporter family protein | 397 | zinc/iron permease [Dictyostelium discoideum AX4] E-VALUE:8.00E-19 | PF02535.15 | 42.97 |
| TTHERM_00760450 | hypothetical protein | 352 | viral A-type inclusion protein [Trichomonas vaginalis G3] E-VALUE:8.00E-04 | NA | 33.28 |
| TTHERM_00126860 | hypothetical protein | 815 | viral A-type inclusion protein [Trichomonas vaginalis G3] E-VALUE:2.00E-04 | NA | 38.84 |
| TTHERM_01579320 | hypothetical protein | 437 | viral A-type inclusion protein [Trichomonas vaginalis G3] E-VALUE:0.004 | NA | 32.45 |
| TTHERM_00630280 | hypothetical protein | 92 | unnamed protein product [Vitis vinifera] E-VALUE:6.00E-04 | NA | 38.36 |
| TTHERM_00207260 | Ras family protein | 95 | unnamed protein product [Blastocystis hominis] E-VALUE:4.00E-09 | PF00071.15 | 58.22 |
| TTHERM_00158120 | hypothetical protein | 179 | unknown protein [Arabidopsis thaliana] E-VALUE:0.001 | NA | 43.13 |
| TTHERM_00361950 | hypothetical protein | 154 | tryptophanyl-tRNA synthetase [Candidatus Carsonella ruddii] E-VALUE:0.006 | NA | 31.3 |
| TTHERM_00927350 | hypothetical protein | 706 | tetratricopeptide repeat family [Microscilla marina ATCC 23134] E-VALUE:0.01 | NA | 33.39 |
| TTHERM_01379980 | hypothetical protein | 176 | TATA element modulatory factor [Camponotus floridanus] E-VALUE:0.008 | NA | 40.93 |
| TTHERM_01781750 | hypothetical protein | 366 | roprotein convertase 6C [Xenopus laevis] E-VALUE:1.00E-08 | NA | 37.91 |
| TTHERM_01648010 | hypothetical protein | 79 | RNA polymerase II subunit [Opegrapha vermicellifera] E-VALUE:1.00E-05 | PF00562.21 | 48.48 |
| TTHERM_00125450 | Ribonuclease T2 family protein | 224 | ribonuclease T2 [Dictyostelium discoideum AX4] E-VALUE:8.00E-17 | PF00445.11 | 46.47 |
| TTHERM_00985100 | Ras family protein | 181 | Rab GTPase [Dictyostelium discoideum AX4] E-VALUE:3.00E-15 | PF00071.15 | 34.06 |
| TTHERM_01142650 | hypothetical protein | 144 | Putative surface protein with EGF domains and furin-like repeats [Paramecium tetraurelia strain d4-2] E-VALUE:4.00E-04 | NA | 35.33 |
| TTHERM_00247040 | hypothetical protein | 250 | PREDICTED: similar to transposase (putative) [Hydra magnipapillata] E-VALUE:1.00E-13 | PF01498.11 | 40.55 |
| TTHERM_00912180 | conserved hypothetical protein | 346 | PREDICTED: similar to proprotein convertase 6B [Gallus gallus] E-VALUE:1.00E-07 | NA | 45.03 |
| TTHERM_00773740 | hypothetical protein | 334 | PREDICTED: similar to CG1105 CG1105-PA [Ciona intestinalis] E-VALUE:1.00E-08 | PF02752.15,PF00339.22 | 31.3 |
| TTHERM_00224530 | hypothetical protein | 871 | PREDICTED: proprotein convertase subtilisin/kexin type 5 [Taeniopygia guttata] E-VALUE:8.00E-34 | PF09458.3 | 37.52 |
| TTHERM_00717680 | hypothetical protein | 229 | PREDICTED: proprotein convertase subtilisin/kexin type 5 [Oryctolagus cuniculus] E-VALUE:1.00E-06 | NA | 69.07 |
| TTHERM_00857940 | hypothetical protein | 112 | PREDICTED: lipopolysaccharide-induced TNF-alpha factor-like [Saccoglossus kowalevskii] E-VALUE:8.00E-12 | PF10601.2 | 49.29 |
| TTHERM_01322690 | hypothetical protein | 243 | PREDICTED: Fraser syndrome 1 [Taeniopygia guttata] E-VALUE:2.00E-05 | NA | 59.08 |
| TTHERM_00566950 | hypothetical protein | 261 | PREDICTED: Fraser syndrome 1 [Taeniopygia guttata] E-VALUE:1.00E-06 | NA | 41.62 |
| TTHERM_00857840 | hypothetical protein | 374 | predicted protein [Naegleria gruberi] E-VALUE:2.00E-07 | NA | 87.32 |
| TTHERM_00028710 | Deoxyribonuclease II family protein | 341 | plancitoxin I [Acanthaster planci] E-VALUE:4.00E-41 | PF03265.8 | 60.69 |
| TTHERM_00559780 | hypothetical protein | 475 | PC6B [Mus musculus] E-VALUE:8.00E-13 | NA | 63.9 |
| TTHERM_00661630 | Glutathione S-transferase, N-terminal domain containing protein | 245 | 26.5 kDa glutathione transferase mutant K148/R [synthetic construct] E-VALUE:8.00E-24 | PF00043.18 | 62.35 |
| TTHERM_00405510 | hypothetical protein | 41 | allergen Aca s 8 [Acarus siro] E-VALUE:0.004 | PF02798.13 | 37.58 |
| TTHERM_00035320 | ABC transporter family protein | 628 | ATP-binding Cassette (ABC) Superfamily [Phytophthora infestans T30-4] E-VALUE:4.00E-88 | PF00005.20,PF01061.17 | 65.83 |
| TTHERM_00538680 | hypothetical protein | 167 | Beige/BEACH domain containing protein [Trichomonas vaginalis G3] gb | NA | 36.57 |
| TTHERM_00399520 | hypothetical protein | 208 | cathepsin D [Oryctolagus cuniculus] E-VALUE:5.00E-04 | PF00026.16 | 37.2 |
| TTHERM_01119400 | hypothetical protein | 211 | CDPK2 [Plasmodium falciparum] E-VALUE:1.00E-09 | PF00069.18 | 32.49 |
| TTHERM_01590550 | hypothetical protein | 546 | cell division protein [Chaetosphaeridium globosum] E-VALUE:0.001 | NA | 32.56 |
| TTHERM_00488320 | TPR Domain containing protein | 171 | Chain A, Design Of Stable Alpha-Helical Arrays From An Idealized Tpr Motif pdb|1NA0|B Chain B, Design Of Stable Alpha-Helical Arrays From An Idealized Tpr Motif E-VALUE:3.00E-12 | PF00515.21 | 30.16 |
| TTHERM_00783220 | hypothetical protein | 548 | chromosome assembly protein [Aquifex aeolicus VF5] E-VALUE:3.00E-04 | NA | 34.63 |
| TTHERM_01277500 | hypothetical protein | 313 | chromosome segregation ATPase-like protein [Trichodesmium erythraeum IMS101] E-VALUE:5.00E-04 | NA | 37.25 |
| TTHERM_00259340 | hypothetical protein | 200 | conserved Plasmodium membrane protein [Plasmodium falciparum 3D7] E-VALUE:0.002 | NA | 40.67 |
| TTHERM_01130800 | hypothetical protein | 172 | conserved Plasmodium protein [Plasmodium falciparum 3D7] E-VALUE:0.005 | NA | 38.49 |
| TTHERM_00256760 | hypothetical protein | 1322 | conserved Plasmodium protein [Plasmodium falciparum 3D7] E-VALUE:4.00E-08 | NA | 39.7 |
| TTHERM_00666110 | hypothetical protein | 211 | conserved Plasmodium protein [Plasmodium falciparum 3D7] E-VALUE:5.00E-04 | NA | 38.12 |
| TTHERM_00609370 | hypothetical protein | 144 | conserved Plasmodium protein [Plasmodium falciparum 3D7] E-VALUE:8.00E-04 | NA | 31.4 |
| TTHERM_00361650 | hypothetical protein | 658 | conserved Plasmodium protein, unknown function [Plasmodium falciparum 3D7] E-VALUE:7.00E-06 | NA | 35.12 |
| TTHERM_01207680 | oxalate/formate exchange protein | 435 | conserved unknown protein [Ectocarpus siliculosus] E-VALUE:2.00E-17 | PF07690.9 | 33.76 |
| TTHERM_00399620 | hypothetical protein | 246 | COX1 intron 3 ORF [Kluyveromyces lactis] E-VALUE:3.00E-04 | NA | 35.43 |
| TTHERM_01323740 | hypothetical protein | 242 | furin 2 [Drosophila erecta]? E-VALUE:6.00E-06 | NA | 81.81 |
| TTHERM_01202180 | Extracellular matrix-like protein, putative | 237 | furin2, putative [Ixodes scapularis] E-VALUE:3.00E-06 | NA | 36.34 |
| TTHERM_01201170 | conserved hypothetical protein | 305 | GA15057 [Drosophila pseudoobscura pseudoobscura] E-VALUE:1.00E-08 | NA | 40.39 |
| TTHERM_01317360 | hypothetical protein | 406 | GF19273 [Drosophila ananassae]? E-VALUE:6.00E-09 | NA | 33.11 |
| TTHERM_00801270 | hypothetical protein | 211 | GRIP and coiled-coil domain-containing protein 1 [Culex quinquefasciatus] E-VALUE:9.00E-04 | NA | 39.91 |
| TTHERM_01473460 | hypothetical protein | 138 | histidine kinase [Polysphondylium pallidum PN500] E-VALUE:0.002 | NA | 43.07 |
| TTHERM_01433570 | hypothetical protein | 285 | hypothetical protein [Paramecium tetraurelia strain d4-2] E-VALUE:1.00E-08 | NA | 78.1 |
| TTHERM_00627290 | hypothetical protein | 230 | hypothetical protein [Paramecium tetraurelia strain d4-2] E-VALUE:3.00E-07 | NA | 33.74 |
| TTHERM_00911110 | conserved hypothetical protein | 250 | hypothetical protein [Paramecium tetraurelia strain d4-2] E-VALUE:4.00E-12 | NA | 39.24 |
| TTHERM_00102610 | hypothetical protein | 1019 | hypothetical protein [Trichomonas vaginalis G3] E-VALUE:3.00E-33 | NA | 35.11 |
| TTHERM_01194840 | hypothetical protein | 63 | Hypothetical protein CBG11703 [Caenorhabditis briggsae] E-VALUE:0.004 | NA | 65.07 |
| TTHERM_01194860 | hypothetical protein | 401 | Hypothetical protein CBG11703 [Caenorhabditis briggsae] E-VALUE:0.006 | NA | 61.61 |
| TTHERM_00161810 | cation channel family protein | 324 | K+-channel protein PAK2.4 [Paramecium tetraurelia] E-VALUE:8.00E-19 | PF07885.9 | 37.66 |
| TTHERM_02139630 | Mitochondrial carrier protein | 178 | mitochondrial ADP/ATP translocator [Chlamydomonas incerta] E-VALUE:4.00E-26 | PF00153.20 | 41.41 |
| TTHERM_00409121 | potassium channel beta | 352 | voltage-gated potassium channel beta 2 subunit [Anophryoides haemophila] E-VALUE:5.00E-83 | PF00248.14 | NA |
| TTHERM_00194149 | chromatin regulatory protein sir2 | 280 | predicted protein [Nematostella vectensis] E-VALUE:9.00E-49 | PF02146.10 | NA |
|  |  |  |  |  |  |
| **Class 3. Gene model gave no blast hits to other organisms** | | | |  |  |
| TTHERM_00621630 | hypothetical protein | 111 | No hits in other organism | PF10203.2 | 44.72 |
| TTHERM_00424490 | hypothetical protein | 56 | No hits in other organism | PF05699.7 | 62.78 |
| TTHERM_00703770 | hypothetical protein | 70 | No hits in other organism | PF04708.5 | 29.61 |
| TTHERM_00085420 | hypothetical protein | 192 | No hits in other organism | PF03824.9 | 43.62 |
| TTHERM_00290620 | hypothetical protein | 103 | No hits in other organism | NA | 97.43 |
| TTHERM_01028890 | hypothetical protein | 79 | No hits in other organism | NA | 95.78 |
| TTHERM_00245260 | hypothetical protein | 58 | No hits in other organism | NA | 93.82 |
| TTHERM_00185170 | hypothetical protein | 123 | No hits in other organism | NA | 93.22 |
| TTHERM_00599680 | hypothetical protein | 46 | No hits in other organism | NA | 91.93 |
| TTHERM_00009900 | hypothetical protein | 111 | No hits in other organism | NA | 89.88 |
| TTHERM_00561400 | hypothetical protein | 43 | No hits in other organism | NA | 86.37 |
| TTHERM_00558680 | hypothetical protein | 102 | No hits in other organism | NA | 86.01 |
| TTHERM_00129160 | hypothetical protein | 68 | No hits in other organism | NA | 85.71 |
| TTHERM_01041930 | hypothetical protein | 51 | No hits in other organism | NA | 80.57 |
| TTHERM_00526650 | hypothetical protein | 49 | No hits in other organism | NA | 80.39 |
| TTHERM_00720100 | hypothetical protein | 141 | No hits in other organism | NA | 80.11 |
| TTHERM_00732820 | hypothetical protein | 82 | No hits in other organism | NA | 79.85 |
| TTHERM_00339560 | hypothetical protein | 84 | No hits in other organism | NA | 78.89 |
| TTHERM_00785830 | hypothetical protein | 44 | No hits in other organism | NA | 78.24 |
| TTHERM_01222510 | hypothetical protein | 77 | No hits in other organism | NA | 75.04 |
| TTHERM_01141580 | hypothetical protein | 72 | No hits in other organism | NA | 75.04 |
| TTHERM_00644770 | hypothetical protein | 57 | No hits in other organism | NA | 74.47 |
| TTHERM_01351020 | hypothetical protein | 63 | No hits in other organism | NA | 73.48 |
| TTHERM_00518600 | hypothetical protein | 105 | No hits in other organism | NA | 72.59 |
| TTHERM_00037710 | hypothetical protein | 222 | No hits in other organism | NA | 70.63 |
| TTHERM_01606690 | hypothetical protein | 61 | No hits in other organism | NA | 69.51 |
| TTHERM_00284220 | hypothetical protein | 138 | No hits in other organism | NA | 69.51 |
| TTHERM_01554990 | hypothetical protein | 103 | No hits in other organism | NA | 69.02 |
| TTHERM_00185680 | hypothetical protein | 69 | No hits in other organism | NA | 67.98 |
| TTHERM_00194280 | hypothetical protein | 64 | No hits in other organism | NA | 67.22 |
| TTHERM_00198420 | hypothetical protein | 70 | No hits in other organism | NA | 66.98 |
| TTHERM_00242490 | hypothetical protein | 169 | No hits in other organism | NA | 66.11 |
| TTHERM_01533580 | hypothetical protein | 122 | No hits in other organism | NA | 65.89 |
| TTHERM_00903880 | hypothetical protein | 110 | No hits in other organism | NA | 65.5 |
| TTHERM_00338120 | hypothetical protein | 62 | No hits in other organism | NA | 65.41 |
| TTHERM_02192730 | hypothetical protein | 132 | No hits in other organism | NA | 65.11 |
| TTHERM_00247150 | hypothetical protein | 109 | No hits in other organism | NA | 64.24 |
| TTHERM_00954400 | hypothetical protein | 48 | No hits in other organism | NA | 63.88 |
| TTHERM_00661610 | hypothetical protein | 66 | No hits in other organism | NA | 62.68 |
| TTHERM_00202950 | hypothetical protein | 98 | No hits in other organism | NA | 62.43 |
| TTHERM_00081050 | hypothetical protein | 63 | No hits in other organism | NA | 62.38 |
| TTHERM_01678240 | hypothetical protein | 71 | No hits in other organism | NA | 62.1 |
| TTHERM_01404850 | hypothetical protein | 137 | No hits in other organism | NA | 61.99 |
| TTHERM_00617780 | hypothetical protein | 58 | No hits in other organism | NA | 61.42 |
| TTHERM_00559860 | hypothetical protein | 193 | No hits in other organism | NA | 60.97 |
| TTHERM_00629720 | hypothetical protein | 127 | No hits in other organism | NA | 60.87 |
| TTHERM_00933170 | hypothetical protein | 128 | No hits in other organism | NA | 60.57 |
| TTHERM_00759130 | hypothetical protein | 103 | No hits in other organism | NA | 59.62 |
| TTHERM_00259490 | hypothetical protein | 45 | No hits in other organism | NA | 59.49 |
| TTHERM_00225960 | hypothetical protein | 160 | No hits in other organism | NA | 59.06 |
| TTHERM_00095600 | hypothetical protein | 85 | No hits in other organism | NA | 58.24 |
| TTHERM_00638860 | hypothetical protein | 67 | No hits in other organism | NA | 58.05 |
| TTHERM_00249530 | hypothetical protein | 431 | No hits in other organism | NA | 57.91 |
| TTHERM_00149370 | hypothetical protein | 272 | No hits in other organism | NA | 57.63 |
| TTHERM_00090290 | hypothetical protein | 83 | No hits in other organism | NA | 57.35 |
| TTHERM_00508980 | hypothetical protein | 59 | No hits in other organism | NA | 56.51 |
| TTHERM_00903990 | hypothetical protein | 63 | No hits in other organism | NA | 56.25 |
| TTHERM_00310290 | hypothetical protein | 220 | No hits in other organism | NA | 56.15 |
| TTHERM_00612590 | hypothetical protein | 61 | No hits in other organism | NA | 56.13 |
| TTHERM_00784770 | hypothetical protein | 65 | No hits in other organism | NA | 55.76 |
| TTHERM_01042020 | hypothetical protein | 108 | No hits in other organism | NA | 55.75 |
| TTHERM_00099900 | hypothetical protein | 45 | No hits in other organism | NA | 54.77 |
| TTHERM_01360490 | hypothetical protein | 43 | No hits in other organism | NA | 54.53 |
| TTHERM_01206400 | hypothetical protein | 390 | No hits in other organism | NA | 54.4 |
| TTHERM_00233080 | hypothetical protein | 114 | No hits in other organism | NA | 54.25 |
| TTHERM_00437290 | hypothetical protein | 57 | No hits in other organism | NA | 54.18 |
| TTHERM_00467360 | hypothetical protein | 88 | No hits in other organism | NA | 53.7 |
| TTHERM_02320960 | hypothetical protein | 121 | No hits in other organism | NA | 53.52 |
| TTHERM_00509020 | hypothetical protein | 48 | No hits in other organism | NA | 53.12 |
| TTHERM_00310120 | hypothetical protein | 146 | No hits in other organism | NA | 53.1 |
| TTHERM_00633260 | hypothetical protein | 176 | No hits in other organism | NA | 52.75 |
| TTHERM_00550690 | hypothetical protein | 75 | No hits in other organism | NA | 52.66 |
| TTHERM_00449500 | hypothetical protein | 88 | No hits in other organism | NA | 52.34 |
| TTHERM_00357120 | hypothetical protein | 112 | No hits in other organism | NA | 51.62 |
| TTHERM_01307980 | hypothetical protein | 77 | No hits in other organism | NA | 51.52 |
| TTHERM_00630380 | hypothetical protein | 48 | No hits in other organism | NA | 51.06 |
| TTHERM_02231820 | hypothetical protein | 143 | No hits in other organism | NA | 50.66 |
| TTHERM_00359240 | hypothetical protein | 44 | No hits in other organism | NA | 50.51 |
| TTHERM_00261910 | hypothetical protein | 116 | No hits in other organism | NA | 50.45 |
| TTHERM_00399700 | hypothetical protein | 51 | No hits in other organism | NA | 50.45 |
| TTHERM_00266710 | hypothetical protein | 72 | No hits in other organism | NA | 50.15 |
| TTHERM_00399630 | hypothetical protein | 55 | No hits in other organism | NA | 50.11 |
| TTHERM_00259360 | hypothetical protein | 199 | No hits in other organism | NA | 50.05 |
| TTHERM_00638890 | hypothetical protein | 45 | No hits in other organism | NA | 49.78 |
| TTHERM_01087880 | hypothetical protein | 70 | No hits in other organism | NA | 49.77 |
| TTHERM_00658910 | hypothetical protein | 48 | No hits in other organism | NA | 49.34 |
| TTHERM_00607000 | hypothetical protein | 166 | No hits in other organism | NA | 49.09 |
| TTHERM_00128580 | Zinc finger, C2H2 type family protein | 154 | No hits in other organism | NA | 49.05 |
| TTHERM_01405880 | hypothetical protein | 115 | No hits in other organism | NA | 48.86 |
| TTHERM_00066780 | hypothetical protein | 114 | No hits in other organism | NA | 48.77 |
| TTHERM_00289100 | hypothetical protein | 52 | No hits in other organism | NA | 48.57 |
| TTHERM_00013850 | hypothetical protein | 46 | No hits in other organism | NA | 48.1 |
| TTHERM_00242320 | hypothetical protein | 219 | No hits in other organism | NA | 47.93 |
| TTHERM_00414470 | hypothetical protein | 247 | No hits in other organism | NA | 47.92 |
| TTHERM_00267810 | hypothetical protein | 48 | No hits in other organism | NA | 47.78 |
| TTHERM_00715870 | hypothetical protein | 79 | No hits in other organism | NA | 47.71 |
| TTHERM_00526320 | hypothetical protein | 48 | No hits in other organism | NA | 47.63 |
| TTHERM_01567130 | hypothetical protein | 201 | No hits in other organism | NA | 47.6 |
| TTHERM_00609410 | hypothetical protein | 49 | No hits in other organism | NA | 47.56 |
| TTHERM_00846960 | hypothetical protein | 101 | No hits in other organism | NA | 47.5 |
| TTHERM_00678420 | hypothetical protein | 49 | No hits in other organism | NA | 47.47 |
| TTHERM_01426460 | hypothetical protein | 92 | No hits in other organism | NA | 47.18 |
| TTHERM_01051840 | hypothetical protein | 46 | No hits in other organism | NA | 47.13 |
| TTHERM_02362040 | hypothetical protein | 48 | No hits in other organism | NA | 47.07 |
| TTHERM_01567140 | hypothetical protein | 57 | No hits in other organism | NA | 46.82 |
| TTHERM_01287950 | hypothetical protein | 80 | No hits in other organism | NA | 46.79 |
| TTHERM_00471110 | hypothetical protein | 131 | No hits in other organism | NA | 46.66 |
| TTHERM_00715910 | hypothetical protein | 73 | No hits in other organism | NA | 46.59 |
| TTHERM_00075940 | hypothetical protein | 215 | No hits in other organism | NA | 46.46 |
| TTHERM_01306910 | hypothetical protein | 50 | No hits in other organism | NA | 46.41 |
| TTHERM_01292180 | hypothetical protein | 56 | No hits in other organism | NA | 46.37 |
| TTHERM_00328450 | hypothetical protein | 136 | No hits in other organism | NA | 46.33 |
| TTHERM_00320110 | hypothetical protein | 278 | No hits in other organism | NA | 46.24 |
| TTHERM_00901790 | hypothetical protein | 135 | No hits in other organism | NA | 46.03 |
| TTHERM_00194290 | hypothetical protein | 112 | No hits in other organism | NA | 46.02 |
| TTHERM_00249540 | hypothetical protein | 79 | No hits in other organism | NA | 45.98 |
| TTHERM_01583430 | hypothetical protein | 60 | No hits in other organism | NA | 45.96 |
| TTHERM_00753630 | hypothetical protein | 126 | No hits in other organism | NA | 45.85 |
| TTHERM_02219810 | hypothetical protein | 130 | No hits in other organism | NA | 45.73 |
| TTHERM_01492730 | hypothetical protein | 51 | No hits in other organism | NA | 45.7 |
| TTHERM_01081660 | hypothetical protein | 129 | No hits in other organism | NA | 45.67 |
| TTHERM_01431550 | hypothetical protein | 55 | No hits in other organism | NA | 45.64 |
| TTHERM_00348270 | hypothetical protein | 50 | No hits in other organism | NA | 45.58 |
| TTHERM_00558610 | hypothetical protein | 53 | No hits in other organism | NA | 45.48 |
| TTHERM_00491080 | hypothetical protein | 60 | No hits in other organism | NA | 45.36 |
| TTHERM_00090410 | hypothetical protein | 244 | No hits in other organism | NA | 45 |
| TTHERM_00745810 | hypothetical protein | 194 | No hits in other organism | NA | 44.82 |
| TTHERM_01051740 | hypothetical protein | 40 | No hits in other organism | NA | 44.6 |
| TTHERM_01132920 | hypothetical protein | 50 | No hits in other organism | NA | 44.52 |
| TTHERM_01387110 | hypothetical protein | 99 | No hits in other organism | NA | 44.5 |
| TTHERM_01470380 | hypothetical protein | 42 | No hits in other organism | NA | 44.48 |
| TTHERM_00569240 | hypothetical protein | 54 | No hits in other organism | NA | 44.32 |
| TTHERM_01002810 | hypothetical protein | 92 | No hits in other organism | NA | 44.28 |
| TTHERM_00802370 | hypothetical protein | 46 | No hits in other organism | NA | 44.18 |
| TTHERM_00656230 | hypothetical protein | 91 | No hits in other organism | NA | 44.16 |
| TTHERM_00422210 | hypothetical protein | 82 | No hits in other organism | NA | 44.15 |
| TTHERM_00463780 | hypothetical protein | 48 | No hits in other organism | NA | 44.05 |
| TTHERM_01032280 | hypothetical protein | 178 | No hits in other organism | NA | 44.04 |
| TTHERM_00391370 | hypothetical protein | 212 | No hits in other organism | NA | 43.98 |
| TTHERM_01718400 | hypothetical protein | 206 | No hits in other organism | NA | 43.98 |
| TTHERM_00024040 | hypothetical protein | 64 | No hits in other organism | NA | 43.97 |
| TTHERM_00090390 | hypothetical protein | 62 | No hits in other organism | NA | 43.96 |
| TTHERM_01353220 | hypothetical protein | 534 | No hits in other organism | NA | 43.9 |
| TTHERM_01066990 | hypothetical protein | 62 | No hits in other organism | NA | 43.83 |
| TTHERM_00201590 | hypothetical protein | 69 | No hits in other organism | NA | 43.79 |
| TTHERM_00105050 | hypothetical protein | 48 | No hits in other organism | NA | 43.73 |
| TTHERM_00128550 | hypothetical protein | 56 | No hits in other organism | NA | 43.63 |
| TTHERM_00424650 | hypothetical protein | 40 | No hits in other organism | NA | 43.62 |
| TTHERM_02598230 | hypothetical protein | 194 | No hits in other organism | NA | 43.58 |
| TTHERM_01122640 | hypothetical protein | 53 | No hits in other organism | NA | 43.53 |
| TTHERM_00161020 | hypothetical protein | 47 | No hits in other organism | NA | 43.5 |
| TTHERM_00581880 | hypothetical protein | 131 | No hits in other organism | NA | 43.41 |
| TTHERM_00011050 | hypothetical protein | 165 | No hits in other organism | NA | 43.35 |
| TTHERM_00937720 | hypothetical protein | 132 | No hits in other organism | NA | 43.28 |
| TTHERM_00686020 | hypothetical protein | 75 | No hits in other organism | NA | 43.26 |
| TTHERM_00535940 | hypothetical protein | 57 | No hits in other organism | NA | 43.03 |
| TTHERM_00053760 | hypothetical protein | 358 | No hits in other organism | NA | 42.89 |
| TTHERM_01147180 | hypothetical protein | 90 | No hits in other organism | NA | 42.75 |
| TTHERM_00434050 | hypothetical protein | 60 | No hits in other organism | NA | 42.68 |
| TTHERM_00860540 | hypothetical protein | 56 | No hits in other organism | NA | 42.68 |
| TTHERM_01704350 | hypothetical protein | 103 | No hits in other organism | NA | 42.58 |
| TTHERM_01485700 | hypothetical protein | 47 | No hits in other organism | NA | 42.46 |
| TTHERM_00295850 | hypothetical protein | 77 | No hits in other organism | NA | 42.42 |
| TTHERM_00113070 | hypothetical protein | 96 | No hits in other organism | NA | 42.26 |
| TTHERM_00561160 | hypothetical protein | 183 | No hits in other organism | NA | 42.19 |
| TTHERM_00125730 | hypothetical protein | 251 | No hits in other organism | NA | 42.18 |
| TTHERM_00693100 | hypothetical protein | 164 | No hits in other organism | NA | 42.09 |
| TTHERM_00735350 | hypothetical protein | 185 | No hits in other organism | NA | 42.07 |
| TTHERM_00886920 | hypothetical protein | 138 | No hits in other organism | NA | 42.06 |
| TTHERM_00582350 | hypothetical protein | 571 | No hits in other organism | NA | 41.97 |
| TTHERM_01366660 | hypothetical protein | 39 | No hits in other organism | NA | 41.93 |
| TTHERM_00873610 | hypothetical protein | 58 | No hits in other organism | NA | 41.91 |
| TTHERM_00543700 | hypothetical protein | 82 | No hits in other organism | NA | 41.88 |
| TTHERM_00678520 | hypothetical protein | 206 | No hits in other organism | NA | 41.88 |
| TTHERM_00888070 | hypothetical protein | 91 | No hits in other organism | NA | 41.81 |
| TTHERM_00077180 | hypothetical protein | 103 | No hits in other organism | NA | 41.78 |
| TTHERM_00812650 | hypothetical protein | 63 | No hits in other organism | NA | 41.74 |
| TTHERM_00667030 | hypothetical protein | 44 | No hits in other organism | NA | 41.73 |
| TTHERM_02452140 | hypothetical protein | 199 | No hits in other organism | NA | 41.7 |
| TTHERM_00603940 | hypothetical protein | 90 | No hits in other organism | NA | 41.63 |
| TTHERM_00397000 | hypothetical protein | 43 | No hits in other organism | NA | 41.61 |
| TTHERM_00100100 | hypothetical protein | 52 | No hits in other organism | NA | 41.61 |
| TTHERM_01548910 | hypothetical protein | 55 | No hits in other organism | NA | 41.55 |
| TTHERM_00020730 | conserved hypothetical protein | 98 | No hits in other organism | NA | 41.54 |
| TTHERM_00612580 | hypothetical protein | 208 | No hits in other organism | NA | 41.53 |
| TTHERM_00847050 | hypothetical protein | 95 | No hits in other organism | NA | 41.39 |
| TTHERM_00527030 | hypothetical protein | 163 | No hits in other organism | NA | 41.38 |
| TTHERM_00434130 | hypothetical protein | 314 | No hits in other organism | NA | 41.31 |
| TTHERM_00130020 | hypothetical protein | 41 | No hits in other organism | NA | 41.28 |
| TTHERM_01462170 | hypothetical protein | 50 | No hits in other organism | NA | 41.26 |
| TTHERM_00036820 | hypothetical protein | 75 | No hits in other organism | NA | 41.16 |
| TTHERM_00526900 | hypothetical protein | 68 | No hits in other organism | NA | 41.03 |
| TTHERM_00001290 | hypothetical protein | 66 | No hits in other organism | NA | 41.01 |
| TTHERM_00411370 | hypothetical protein | 43 | No hits in other organism | NA | 40.94 |
| TTHERM_00313790 | hypothetical protein | 81 | No hits in other organism | NA | 40.94 |
| TTHERM_00893360 | hypothetical protein | 33 | No hits in other organism | NA | 40.88 |
| TTHERM_00489430 | hypothetical protein | 52 | No hits in other organism | NA | 40.86 |
| TTHERM_00903980 | hypothetical protein | 52 | No hits in other organism | NA | 40.84 |
| TTHERM_01138320 | hypothetical protein | 479 | No hits in other organism | NA | 40.83 |
| TTHERM_00208260 | hypothetical protein | 41 | No hits in other organism | NA | 40.74 |
| TTHERM_00490770 | hypothetical protein | 117 | No hits in other organism | NA | 40.57 |
| TTHERM_00933330 | hypothetical protein | 197 | No hits in other organism | NA | 40.45 |
| TTHERM_00678230 | hypothetical protein | 45 | No hits in other organism | NA | 40.44 |
| TTHERM_02602230 | hypothetical protein | 145 | No hits in other organism | NA | 40.43 |
| TTHERM_00194750 | hypothetical protein | 55 | No hits in other organism | NA | 40.32 |
| TTHERM_01566130 | hypothetical protein | 29 | No hits in other organism | NA | 40.29 |
| TTHERM_00527250 | hypothetical protein | 63 | No hits in other organism | NA | 40.28 |
| TTHERM_00099850 | hypothetical protein | 62 | No hits in other organism | NA | 40.24 |
| TTHERM_00628620 | hypothetical protein | 63 | No hits in other organism | NA | 40.17 |
| TTHERM_00277240 | hypothetical protein | 242 | No hits in other organism | NA | 40.15 |
| TTHERM_01614780 | hypothetical protein | 54 | No hits in other organism | NA | 40.11 |
| TTHERM_01377890 | hypothetical protein | 44 | No hits in other organism | NA | 40.09 |
| TTHERM_01144920 | hypothetical protein | 73 | No hits in other organism | NA | 39.97 |
| TTHERM_01386090 | hypothetical protein | 50 | No hits in other organism | NA | 39.96 |
| TTHERM_01054300 | hypothetical protein | 48 | No hits in other organism | NA | 39.9 |
| TTHERM_00471700 | hypothetical protein | 86 | No hits in other organism | NA | 39.89 |
| TTHERM_01493740 | hypothetical protein | 59 | No hits in other organism | NA | 39.8 |
| TTHERM_01738570 | hypothetical protein | 43 | No hits in other organism | NA | 39.77 |
| TTHERM_00652510 | hypothetical protein | 195 | No hits in other organism | NA | 39.75 |
| TTHERM_00006480 | cell surface immobilization antigen SerH6, putative | 118 | No hits in other organism | NA | 39.66 |
| TTHERM_00170120 | SMA-9 class B, putative | 154 | No hits in other organism | NA | 39.55 |
| TTHERM_00581960 | hypothetical protein | 95 | No hits in other organism | NA | 39.5 |
| TTHERM_00973010 | hypothetical protein | 46 | No hits in other organism | NA | 39.49 |
| TTHERM_00488300 | hypothetical protein | 41 | No hits in other organism | NA | 39.46 |
| TTHERM_00486620 | hypothetical protein | 91 | No hits in other organism | NA | 39.44 |
| TTHERM_00643450 | hypothetical protein | 404 | No hits in other organism | NA | 39.38 |
| TTHERM_01200170 | hypothetical protein | 40 | No hits in other organism | NA | 39.36 |
| TTHERM_00641310 | hypothetical protein | 162 | No hits in other organism | NA | 39.28 |
| TTHERM_01431560 | hypothetical protein | 70 | No hits in other organism | NA | 39.22 |
| TTHERM_01281690 | hypothetical protein | 44 | No hits in other organism | NA | 39.22 |
| TTHERM_00353520 | hypothetical protein | 45 | No hits in other organism | NA | 39.15 |
| TTHERM_01117340 | hypothetical protein | 72 | No hits in other organism | NA | 39.14 |
| TTHERM_00031780 | hypothetical protein | 49 | No hits in other organism | NA | 39.11 |
| TTHERM_01276430 | hypothetical protein | 48 | No hits in other organism | NA | 39.02 |
| TTHERM_01402790 | hypothetical protein | 75 | No hits in other organism | NA | 39 |
| TTHERM_00075710 | hypothetical protein | 57 | No hits in other organism | NA | 38.99 |
| TTHERM_00925510 | hypothetical protein | 82 | No hits in other organism | NA | 38.97 |
| TTHERM_01227890 | hypothetical protein | 48 | No hits in other organism | NA | 38.88 |
| TTHERM_00328460 | hypothetical protein | 61 | No hits in other organism | NA | 38.84 |
| TTHERM_02320950 | hypothetical protein | 197 | No hits in other organism | NA | 38.81 |
| TTHERM_01339600 | hypothetical protein | 67 | No hits in other organism | NA | 38.75 |
| TTHERM_00827240 | hypothetical protein | 93 | No hits in other organism | NA | 38.75 |
| TTHERM_00204170 | hypothetical protein | 46 | No hits in other organism | NA | 38.63 |
| TTHERM_00401840 | hypothetical protein | 103 | No hits in other organism | NA | 38.58 |
| TTHERM_00420890 | hypothetical protein | 57 | No hits in other organism | NA | 38.52 |
| TTHERM_00267830 | hypothetical protein | 70 | No hits in other organism | NA | 38.51 |
| TTHERM_00274490 | hypothetical protein | 41 | No hits in other organism | NA | 38.45 |
| TTHERM_00485890 | hypothetical protein | 77 | No hits in other organism | NA | 38.43 |
| TTHERM_00433710 | hypothetical protein | 66 | No hits in other organism | NA | 38.37 |
| TTHERM_00705110 | hypothetical protein | 57 | No hits in other organism | NA | 38.37 |
| TTHERM_00578920 | hypothetical protein | 81 | No hits in other organism | NA | 38.37 |
| TTHERM_01377920 | hypothetical protein | 111 | No hits in other organism | NA | 38.35 |
| TTHERM_00170130 | hypothetical protein | 59 | No hits in other organism | NA | 38.3 |
| TTHERM_01562090 | hypothetical protein | 53 | No hits in other organism | NA | 38.18 |
| TTHERM_00757690 | hypothetical protein | 40 | No hits in other organism | NA | 38.04 |
| TTHERM_00081060 | hypothetical protein | 70 | No hits in other organism | NA | 38.03 |
| TTHERM_00408800 | hypothetical protein | 45 | No hits in other organism | NA | 37.92 |
| TTHERM_00371180 | hypothetical protein | 45 | No hits in other organism | NA | 37.89 |
| TTHERM_00256880 | hypothetical protein | 51 | No hits in other organism | NA | 37.89 |
| TTHERM_00865020 | hypothetical protein | 44 | No hits in other organism | NA | 37.84 |
| TTHERM_01346850 | hypothetical protein | 82 | No hits in other organism | NA | 37.82 |
| TTHERM_00138510 | hypothetical protein | 57 | No hits in other organism | NA | 37.81 |
| TTHERM_00492630 | hypothetical protein | 484 | No hits in other organism | NA | 37.8 |
| TTHERM_01743570 | hypothetical protein | 98 | No hits in other organism | NA | 37.69 |
| TTHERM_00935570 | hypothetical protein | 48 | No hits in other organism | NA | 37.68 |
| TTHERM_00414500 | hypothetical protein | 79 | No hits in other organism | NA | 37.66 |
| TTHERM_01066960 | hypothetical protein | 60 | No hits in other organism | NA | 37.66 |
| TTHERM_00346590 | hypothetical protein | 74 | No hits in other organism | NA | 37.55 |
| TTHERM_00873700 | hypothetical protein | 361 | No hits in other organism | NA | 37.5 |
| TTHERM_00261970 | hypothetical protein | 113 | No hits in other organism | NA | 37.48 |
| TTHERM_00343810 | hypothetical protein | 51 | No hits in other organism | NA | 37.45 |
| TTHERM_01223650 | hypothetical protein | 41 | No hits in other organism | NA | 37.42 |
| TTHERM_00833650 | hypothetical protein | 82 | No hits in other organism | NA | 37.42 |
| TTHERM_01353110 | hypothetical protein | 60 | No hits in other organism | NA | 37.4 |
| TTHERM_01280610 | hypothetical protein | 54 | No hits in other organism | NA | 37.38 |
| TTHERM_01132930 | hypothetical protein | 105 | No hits in other organism | NA | 37.37 |
| TTHERM_00035210 | hypothetical protein | 87 | No hits in other organism | NA | 37.23 |
| TTHERM_00234150 | hypothetical protein | 100 | No hits in other organism | NA | 37.16 |
| TTHERM_00994210 | hypothetical protein | 58 | No hits in other organism | NA | 37.09 |
| TTHERM_00227840 | hypothetical protein | 191 | No hits in other organism | NA | 37.08 |
| TTHERM_00933240 | hypothetical protein | 46 | No hits in other organism | NA | 37.05 |
| TTHERM_00693120 | hypothetical protein | 99 | No hits in other organism | NA | 37 |
| TTHERM_00305490 | hypothetical protein | 68 | No hits in other organism | NA | 36.97 |
| TTHERM_00724790 | hypothetical protein | 54 | No hits in other organism | NA | 36.91 |
| TTHERM_00191540 | hypothetical protein | 108 | No hits in other organism | NA | 36.89 |
| TTHERM_00644740 | hypothetical protein | 49 | No hits in other organism | NA | 36.89 |
| TTHERM_00001500 | hypothetical protein | 52 | No hits in other organism | NA | 36.86 |
| TTHERM_00939220 | hypothetical protein | 100 | No hits in other organism | NA | 36.85 |
| TTHERM_00644580 | hypothetical protein | 146 | No hits in other organism | NA | 36.82 |
| TTHERM_00007550 | hypothetical protein | 100 | No hits in other organism | NA | 36.82 |
| TTHERM_01222580 | hypothetical protein | 64 | No hits in other organism | NA | 36.81 |
| TTHERM_00957560 | hypothetical protein | 68 | No hits in other organism | NA | 36.8 |
| TTHERM_01498880 | hypothetical protein | 91 | No hits in other organism | NA | 36.76 |
| TTHERM_00264750 | hypothetical protein | 54 | No hits in other organism | NA | 36.74 |
| TTHERM_00641290 | hypothetical protein | 146 | No hits in other organism | NA | 36.69 |
| TTHERM_01135140 | hypothetical protein | 33 | No hits in other organism | NA | 36.65 |
| TTHERM_00633290 | hypothetical protein | 45 | No hits in other organism | NA | 36.54 |
| TTHERM_00644700 | hypothetical protein | 76 | No hits in other organism | NA | 36.54 |
| TTHERM_00069630 | hypothetical protein | 53 | No hits in other organism | NA | 36.54 |
| TTHERM_00892360 | hypothetical protein | 52 | No hits in other organism | NA | 36.53 |
| TTHERM_00721160 | hypothetical protein | 69 | No hits in other organism | NA | 36.5 |
| TTHERM_00340020 | hypothetical protein | 201 | No hits in other organism | NA | 36.46 |
| TTHERM_00425940 | hypothetical protein | 57 | No hits in other organism | NA | 36.42 |
| TTHERM_00581700 | hypothetical protein | 77 | No hits in other organism | NA | 36.4 |
| TTHERM_00560080 | hypothetical protein | 43 | No hits in other organism | NA | 36.39 |
| TTHERM_00892340 | hypothetical protein | 151 | No hits in other organism | NA | 36.38 |
| TTHERM_00826990 | hypothetical protein | 58 | No hits in other organism | NA | 36.31 |
| TTHERM_01154680 | hypothetical protein | 44 | No hits in other organism | NA | 36.28 |
| TTHERM_00384750 | hypothetical protein | 73 | No hits in other organism | NA | 36.27 |
| TTHERM_00198120 | hypothetical protein | 206 | No hits in other organism | NA | 36.25 |
| TTHERM_00274440 | hypothetical protein | 87 | No hits in other organism | NA | 36.21 |
| TTHERM_00274420 | hypothetical protein | 45 | No hits in other organism | NA | 36.19 |
| TTHERM_00913420 | hypothetical protein | 46 | No hits in other organism | NA | 36.18 |
| TTHERM_01080520 | hypothetical protein | 157 | No hits in other organism | NA | 36.17 |
| TTHERM_00734020 | hypothetical protein | 46 | No hits in other organism | NA | 36.15 |
| TTHERM_00035730 | hypothetical protein | 46 | No hits in other organism | NA | 36.14 |
| TTHERM_00399680 | hypothetical protein | 42 | No hits in other organism | NA | 36.11 |
| TTHERM_00030410 | hypothetical protein | 48 | No hits in other organism | NA | 36.1 |
| TTHERM_00191970 | hypothetical protein | 63 | No hits in other organism | NA | 36.02 |
| TTHERM_00564040 | hypothetical protein | 161 | No hits in other organism | NA | 36.01 |
| TTHERM_00585440 | hypothetical protein | 80 | No hits in other organism | NA | 35.95 |
| TTHERM_00721490 | hypothetical protein | 64 | No hits in other organism | NA | 35.95 |
| TTHERM_00794330 | hypothetical protein | 55 | No hits in other organism | NA | 35.95 |
| TTHERM_01253470 | hypothetical protein | 25 | No hits in other organism | NA | 35.92 |
| TTHERM_00047500 | hypothetical protein | 105 | No hits in other organism | NA | 35.91 |
| TTHERM_00499450 | hypothetical protein | 91 | No hits in other organism | NA | 35.89 |
| TTHERM_00289120 | hypothetical protein | 103 | No hits in other organism | NA | 35.87 |
| TTHERM_00313760 | hypothetical protein | 41 | No hits in other organism | NA | 35.85 |
| TTHERM_01608710 | hypothetical protein | 40 | No hits in other organism | NA | 35.85 |
| TTHERM_00080040 | hypothetical protein | 50 | No hits in other organism | NA | 35.84 |
| TTHERM_00170410 | hypothetical protein | 41 | No hits in other organism | NA | 35.83 |
| TTHERM_00633620 | hypothetical protein | 42 | No hits in other organism | NA | 35.82 |
| TTHERM_00233040 | hypothetical protein | 62 | No hits in other organism | NA | 35.82 |
| TTHERM_00753600 | hypothetical protein | 126 | No hits in other organism | NA | 35.79 |
| TTHERM_00522510 | hypothetical protein | 48 | No hits in other organism | NA | 35.78 |
| TTHERM_00540270 | hypothetical protein | 90 | No hits in other organism | NA | 35.75 |
| TTHERM_01065950 | hypothetical protein | 70 | No hits in other organism | NA | 35.75 |
| TTHERM_00329810 | hypothetical protein | 55 | No hits in other organism | NA | 35.74 |
| TTHERM_00321660 | hypothetical protein | 40 | No hits in other organism | NA | 35.73 |
| TTHERM_01469330 | hypothetical protein | 49 | No hits in other organism | NA | 35.73 |
| TTHERM_01132900 | hypothetical protein | 44 | No hits in other organism | NA | 35.72 |
| TTHERM_01351070 | hypothetical protein | 50 | No hits in other organism | NA | 35.7 |
| TTHERM_00927180 | hypothetical protein | 57 | No hits in other organism | NA | 35.7 |
| TTHERM_00624580 | hypothetical protein | 77 | No hits in other organism | NA | 35.69 |
| TTHERM_01082900 | hypothetical protein | 88 | No hits in other organism | NA | 35.69 |
| TTHERM_01270150 | hypothetical protein | 93 | No hits in other organism | NA | 35.6 |
| TTHERM_00621260 | hypothetical protein | 67 | No hits in other organism | NA | 35.58 |
| TTHERM_00697560 | hypothetical protein | 43 | No hits in other organism | NA | 35.58 |
| TTHERM_01028820 | hypothetical protein | 72 | No hits in other organism | NA | 35.56 |
| TTHERM_00136310 | hypothetical protein | 49 | No hits in other organism | NA | 35.47 |
| TTHERM_00233140 | hypothetical protein | 63 | No hits in other organism | NA | 35.45 |
| TTHERM_00171900 | hypothetical protein | 67 | No hits in other organism | NA | 35.43 |
| TTHERM_00259590 | hypothetical protein | 71 | No hits in other organism | NA | 35.42 |
| TTHERM_00760660 | hypothetical protein | 101 | No hits in other organism | NA | 35.41 |
| TTHERM_00187070 | hypothetical protein | 165 | No hits in other organism | NA | 35.41 |
| TTHERM_00628700 | hypothetical protein | 58 | No hits in other organism | NA | 35.39 |
| TTHERM_00147460 | hypothetical protein | 90 | No hits in other organism | NA | 35.37 |
| TTHERM_00992850 | hypothetical protein | 60 | No hits in other organism | NA | 35.37 |
| TTHERM_00355680 | hypothetical protein | 65 | No hits in other organism | NA | 35.35 |
| TTHERM_00579130 | hypothetical protein | 60 | No hits in other organism | NA | 35.32 |
| TTHERM_00540210 | hypothetical protein | 42 | No hits in other organism | NA | 35.31 |
| TTHERM_01352100 | hypothetical protein | 82 | No hits in other organism | NA | 35.29 |
| TTHERM_01281680 | hypothetical protein | 47 | No hits in other organism | NA | 35.27 |
| TTHERM_00049410 | hypothetical protein | 47 | No hits in other organism | NA | 35.27 |
| TTHERM_00320540 | hypothetical protein | 105 | No hits in other organism | NA | 35.27 |
| TTHERM_00218960 | hypothetical protein | 51 | No hits in other organism | NA | 35.26 |
| TTHERM_01253370 | hypothetical protein | 90 | No hits in other organism | NA | 35.22 |
| TTHERM_01100370 | hypothetical protein | 52 | No hits in other organism | NA | 35.21 |
| TTHERM_01136140 | hypothetical protein | 44 | No hits in other organism | NA | 35.19 |
| TTHERM_00628370 | hypothetical protein | 79 | No hits in other organism | NA | 35.14 |
| TTHERM_00873710 | hypothetical protein | 285 | No hits in other organism | NA | 35.07 |
| TTHERM_00145580 | hypothetical protein | 40 | No hits in other organism | NA | 35.04 |
| TTHERM_00399650 | hypothetical protein | 50 | No hits in other organism | NA | 35.04 |
| TTHERM_00171740 | hypothetical protein | 79 | No hits in other organism | NA | 34.98 |
| TTHERM_01300740 | hypothetical protein | 328 | No hits in other organism | NA | 34.97 |
| TTHERM_00616530 | hypothetical protein | 56 | No hits in other organism | NA | 34.96 |
| TTHERM_00145460 | hypothetical protein | 85 | No hits in other organism | NA | 34.96 |
| TTHERM_00489490 | hypothetical protein | 94 | No hits in other organism | NA | 34.94 |
| TTHERM_01031220 | hypothetical protein | 78 | No hits in other organism | NA | 34.93 |
| TTHERM_00313240 | hypothetical protein | 50 | No hits in other organism | NA | 34.91 |
| TTHERM_00865330 | hypothetical protein | 60 | No hits in other organism | NA | 34.91 |
| TTHERM_00670230 | hypothetical protein | 64 | No hits in other organism | NA | 34.88 |
| TTHERM_00343860 | hypothetical protein | 74 | No hits in other organism | NA | 34.87 |
| TTHERM_01194690 | hypothetical protein | 133 | No hits in other organism | NA | 34.84 |
| TTHERM_00469250 | hypothetical protein | 84 | No hits in other organism | NA | 34.79 |
| TTHERM_00289400 | hypothetical protein | 72 | No hits in other organism | NA | 34.79 |
| TTHERM_00343790 | hypothetical protein | 58 | No hits in other organism | NA | 34.76 |
| TTHERM_00392640 | hypothetical protein | 147 | No hits in other organism | NA | 34.74 |
| TTHERM_00705100 | hypothetical protein | 51 | No hits in other organism | NA | 34.73 |
| TTHERM_00775900 | hypothetical protein | 145 | No hits in other organism | NA | 34.73 |
| TTHERM_00401900 | hypothetical protein | 80 | No hits in other organism | NA | 34.69 |
| TTHERM_00371050 | hypothetical protein | 189 | No hits in other organism | NA | 34.67 |
| TTHERM_01645000 | hypothetical protein | 104 | No hits in other organism | NA | 34.65 |
| TTHERM_00925720 | hypothetical protein | 101 | No hits in other organism | NA | 34.63 |
| TTHERM_00554430 | hypothetical protein | 100 | No hits in other organism | NA | 34.62 |
| TTHERM_01824940 | hypothetical protein | 58 | No hits in other organism | NA | 34.62 |
| TTHERM_00011750 | hypothetical protein | 73 | No hits in other organism | NA | 34.61 |
| TTHERM_00899370 | hypothetical protein | 60 | No hits in other organism | NA | 34.58 |
| TTHERM_01208750 | hypothetical protein | 53 | No hits in other organism | NA | 34.53 |
| TTHERM_01023050 | hypothetical protein | 245 | No hits in other organism | NA | 34.53 |
| TTHERM_00535370 | hypothetical protein | 75 | No hits in other organism | NA | 34.51 |
| TTHERM_00113280 | hypothetical protein | 104 | No hits in other organism | NA | 34.51 |
| TTHERM_00052330 | hypothetical protein | 67 | No hits in other organism | NA | 34.46 |
| TTHERM_01516340 | hypothetical protein | 156 | No hits in other organism | NA | 34.44 |
| TTHERM_01061860 | hypothetical protein | 32 | No hits in other organism | NA | 34.44 |
| TTHERM_00105310 | hypothetical protein | 79 | No hits in other organism | NA | 34.38 |
| TTHERM_00138140 | hypothetical protein | 154 | No hits in other organism | NA | 34.37 |
| TTHERM_00699820 | hypothetical protein | 53 | No hits in other organism | NA | 34.37 |
| TTHERM_01047060 | hypothetical protein | 75 | No hits in other organism | NA | 34.35 |
| TTHERM_00473290 | hypothetical protein | 97 | No hits in other organism | NA | 34.31 |
| TTHERM_00424420 | hypothetical protein | 56 | No hits in other organism | NA | 34.3 |
| TTHERM_00717940 | hypothetical protein | 95 | No hits in other organism | NA | 34.3 |
| TTHERM_00605940 | hypothetical protein | 70 | No hits in other organism | NA | 34.29 |
| TTHERM_00699840 | hypothetical protein | 123 | No hits in other organism | NA | 34.29 |
| TTHERM_01041950 | hypothetical protein | 57 | No hits in other organism | NA | 34.27 |
| TTHERM_00321610 | hypothetical protein | 52 | No hits in other organism | NA | 34.25 |
| TTHERM_00480140 | hypothetical protein | 48 | No hits in other organism | NA | 34.25 |
| TTHERM_01135090 | hypothetical protein | 65 | No hits in other organism | NA | 34.24 |
| TTHERM_00208270 | hypothetical protein | 102 | No hits in other organism | NA | 34.21 |
| TTHERM_00105380 | hypothetical protein | 75 | No hits in other organism | NA | 34.2 |
| TTHERM_00158070 | hypothetical protein | 377 | No hits in other organism | NA | 34.19 |
| TTHERM_01085440 | hypothetical protein | 46 | No hits in other organism | NA | 34.13 |
| TTHERM_00558110 | hypothetical protein | 68 | No hits in other organism | NA | 34.02 |
| TTHERM_00013020 | hypothetical protein | 58 | No hits in other organism | NA | 33.99 |
| TTHERM_00635700 | hypothetical protein | 40 | No hits in other organism | NA | 33.98 |
| TTHERM_01359490 | hypothetical protein | 119 | No hits in other organism | NA | 33.97 |
| TTHERM_00607030 | hypothetical protein | 135 | No hits in other organism | NA | 33.95 |
| TTHERM_00106990 | hypothetical protein | 53 | No hits in other organism | NA | 33.92 |
| TTHERM_01577300 | hypothetical protein | 47 | No hits in other organism | NA | 33.86 |
| TTHERM_00304140 | hypothetical protein | 170 | No hits in other organism | NA | 33.85 |
| TTHERM_00835280 | hypothetical protein | 201 | No hits in other organism | NA | 33.82 |
| TTHERM_01102650 | hypothetical protein | 40 | No hits in other organism | NA | 33.81 |
| TTHERM_01483650 | hypothetical protein | 46 | No hits in other organism | NA | 33.78 |
| TTHERM_00242380 | hypothetical protein | 73 | No hits in other organism | NA | 33.77 |
| TTHERM_00112460 | hypothetical protein | 44 | No hits in other organism | NA | 33.69 |
| TTHERM_00423400 | hypothetical protein | 51 | No hits in other organism | NA | 33.67 |
| TTHERM_00604940 | hypothetical protein | 65 | No hits in other organism | NA | 33.65 |
| TTHERM_00068160 | hypothetical protein | 52 | No hits in other organism | NA | 33.64 |
| TTHERM_00352200 | hypothetical protein | 64 | No hits in other organism | NA | 33.63 |
| TTHERM_00462880 | hypothetical protein | 54 | No hits in other organism | NA | 33.62 |
| TTHERM_00418180 | hypothetical protein | 48 | No hits in other organism | NA | 33.61 |
| TTHERM_00695580 | hypothetical protein | 69 | No hits in other organism | NA | 33.6 |
| TTHERM_00285690 | hypothetical protein | 147 | No hits in other organism | NA | 33.56 |
| TTHERM_00301700 | hypothetical protein | 60 | No hits in other organism | NA | 33.55 |
| TTHERM_00841240 | hypothetical protein | 40 | No hits in other organism | NA | 33.53 |
| TTHERM_00005970 | hypothetical protein | 55 | No hits in other organism | NA | 33.53 |
| TTHERM_01260690 | hypothetical protein | 45 | No hits in other organism | NA | 33.52 |
| TTHERM_01006440 | hypothetical protein | 77 | No hits in other organism | NA | 33.51 |
| TTHERM_00322940 | hypothetical protein | 115 | No hits in other organism | NA | 33.49 |
| TTHERM_00630000 | hypothetical protein | 45 | No hits in other organism | NA | 33.49 |
| TTHERM_01240410 | hypothetical protein | 93 | No hits in other organism | NA | 33.48 |
| TTHERM_00474400 | hypothetical protein | 56 | No hits in other organism | NA | 33.48 |
| TTHERM_01398500 | hypothetical protein | 73 | No hits in other organism | NA | 33.46 |
| TTHERM_00163910 | hypothetical protein | 53 | No hits in other organism | NA | 33.44 |
| TTHERM_01028780 | hypothetical protein | 73 | No hits in other organism | NA | 33.43 |
| TTHERM_00298460 | hypothetical protein | 209 | No hits in other organism | NA | 33.42 |
| TTHERM_00613600 | hypothetical protein | 173 | No hits in other organism | NA | 33.41 |
| TTHERM_00522470 | hypothetical protein | 45 | No hits in other organism | NA | 33.39 |
| TTHERM_00503880 | hypothetical protein | 45 | No hits in other organism | NA | 33.38 |
| TTHERM_00001450 | hypothetical protein | 152 | No hits in other organism | NA | 33.32 |
| TTHERM_00787260 | hypothetical protein | 59 | No hits in other organism | NA | 33.3 |
| TTHERM_00094030 | hypothetical protein | 70 | No hits in other organism | NA | 33.29 |
| TTHERM_00138120 | hypothetical protein | 157 | No hits in other organism | NA | 33.29 |
| TTHERM_00317150 | hypothetical protein | 72 | No hits in other organism | NA | 33.29 |
| TTHERM_00806900 | hypothetical protein | 57 | No hits in other organism | NA | 33.27 |
| TTHERM_00935600 | hypothetical protein | 60 | No hits in other organism | NA | 33.26 |
| TTHERM_01102800 | hypothetical protein | 117 | No hits in other organism | NA | 33.24 |
| TTHERM_00578900 | hypothetical protein | 74 | No hits in other organism | NA | 33.2 |
| TTHERM_00341270 | hypothetical protein | 59 | No hits in other organism | NA | 33.2 |
| TTHERM_00661520 | hypothetical protein | 138 | No hits in other organism | NA | 33.19 |
| TTHERM_01444860 | hypothetical protein | 78 | No hits in other organism | NA | 33.11 |
| TTHERM_00122250 | hypothetical protein | 91 | No hits in other organism | NA | 33.11 |
| TTHERM_00090420 | hypothetical protein | 112 | No hits in other organism | NA | 33.08 |
| TTHERM_01021990 | hypothetical protein | 306 | No hits in other organism | NA | 33.08 |
| TTHERM_00951970 | hypothetical protein | 71 | No hits in other organism | NA | 33.08 |
| TTHERM_01437780 | hypothetical protein | 43 | No hits in other organism | NA | 33.05 |
| TTHERM_00399310 | hypothetical protein | 55 | No hits in other organism | NA | 33.05 |
| TTHERM_00442220 | hypothetical protein | 86 | No hits in other organism | NA | 33.03 |
| TTHERM_01359460 | hypothetical protein | 135 | No hits in other organism | NA | 33 |
| TTHERM_00572200 | hypothetical protein | 89 | No hits in other organism | NA | 32.96 |
| TTHERM_00420240 | hypothetical protein | 55 | No hits in other organism | NA | 32.95 |
| TTHERM_00310700 | hypothetical protein | 173 | No hits in other organism | NA | 32.94 |
| TTHERM_01316360 | hypothetical protein | 66 | No hits in other organism | NA | 32.93 |
| TTHERM_00343700 | hypothetical protein | 44 | No hits in other organism | NA | 32.92 |
| TTHERM_00358160 | hypothetical protein | 56 | No hits in other organism | NA | 32.88 |
| TTHERM_00929410 | hypothetical protein | 53 | No hits in other organism | NA | 32.88 |
| TTHERM_00755850 | hypothetical protein | 67 | No hits in other organism | NA | 32.83 |
| TTHERM_00379090 | hypothetical protein | 55 | No hits in other organism | NA | 32.79 |
| TTHERM_00239170 | hypothetical protein | 56 | No hits in other organism | NA | 32.79 |
| TTHERM_00925860 | hypothetical protein | 72 | No hits in other organism | NA | 32.79 |
| TTHERM_00569220 | hypothetical protein | 44 | No hits in other organism | NA | 32.78 |
| TTHERM_00837790 | hypothetical protein | 67 | No hits in other organism | NA | 32.74 |
| TTHERM_01200160 | hypothetical protein | 62 | No hits in other organism | NA | 32.74 |
| TTHERM_00648780 | hypothetical protein | 52 | No hits in other organism | NA | 32.74 |
| TTHERM_00463310 | hypothetical protein | 78 | No hits in other organism | NA | 32.7 |
| TTHERM_00429640 | hypothetical protein | 104 | No hits in other organism | NA | 32.67 |
| TTHERM_00418330 | hypothetical protein | 72 | No hits in other organism | NA | 32.67 |
| TTHERM_00509150 | hypothetical protein | 123 | No hits in other organism | NA | 32.66 |
| TTHERM_01335290 | hypothetical protein | 44 | No hits in other organism | NA | 32.64 |
| TTHERM_00190980 | hypothetical protein | 212 | No hits in other organism | NA | 32.63 |
| TTHERM_01354240 | hypothetical protein | 157 | No hits in other organism | NA | 32.61 |
| TTHERM_00550680 | hypothetical protein | 60 | No hits in other organism | NA | 32.61 |
| TTHERM_01186280 | hypothetical protein | 56 | No hits in other organism | NA | 32.58 |
| TTHERM_01047040 | hypothetical protein | 118 | No hits in other organism | NA | 32.51 |
| TTHERM_01006500 | hypothetical protein | 43 | No hits in other organism | NA | 32.48 |
| TTHERM_00449310 | hypothetical protein | 40 | No hits in other organism | NA | 32.46 |
| TTHERM_00314950 | hypothetical protein | 50 | No hits in other organism | NA | 32.45 |
| TTHERM_01321620 | hypothetical protein | 89 | No hits in other organism | NA | 32.42 |
| TTHERM_00582360 | hypothetical protein | 652 | No hits in other organism | NA | 32.38 |
| TTHERM_00648620 | hypothetical protein | 46 | No hits in other organism | NA | 32.36 |
| TTHERM_00442820 | hypothetical protein | 71 | No hits in other organism | NA | 32.34 |
| TTHERM_01090210 | hypothetical protein | 66 | No hits in other organism | NA | 32.34 |
| TTHERM_01265010 | hypothetical protein | 43 | No hits in other organism | NA | 32.33 |
| TTHERM_01338450 | hypothetical protein | 40 | No hits in other organism | NA | 32.3 |
| TTHERM_00126960 | hypothetical protein | 58 | No hits in other organism | NA | 32.29 |
| TTHERM_00312540 | hypothetical protein | 81 | No hits in other organism | NA | 32.27 |
| TTHERM_00189210 | hypothetical protein | 52 | No hits in other organism | NA | 32.25 |
| TTHERM_01012080 | hypothetical protein | 111 | No hits in other organism | NA | 32.25 |
| TTHERM_00323190 | hypothetical protein | 65 | No hits in other organism | NA | 32.23 |
| TTHERM_01333200 | hypothetical protein | 45 | No hits in other organism | NA | 32.23 |
| TTHERM_00916440 | hypothetical protein | 78 | No hits in other organism | NA | 32.23 |
| TTHERM_00449530 | hypothetical protein | 48 | No hits in other organism | NA | 32.22 |
| TTHERM_00062640 | hypothetical protein | 86 | No hits in other organism | NA | 32.19 |
| TTHERM_01273310 | hypothetical protein | 95 | No hits in other organism | NA | 32.18 |
| TTHERM_00125630 | hypothetical protein | 47 | No hits in other organism | NA | 32.13 |
| TTHERM_01045790 | hypothetical protein | 42 | No hits in other organism | NA | 32.09 |
| TTHERM_00481200 | hypothetical protein | 111 | No hits in other organism | NA | 32.08 |
| TTHERM_00600290 | hypothetical protein | 44 | No hits in other organism | NA | 32.07 |
| TTHERM_00706370 | hypothetical protein | 44 | No hits in other organism | NA | 31.95 |
| TTHERM_00859390 | hypothetical protein | 86 | No hits in other organism | NA | 31.94 |
| TTHERM_01012120 | hypothetical protein | 41 | No hits in other organism | NA | 31.93 |
| TTHERM_01698330 | hypothetical protein | 45 | No hits in other organism | NA | 31.92 |
| TTHERM_01141640 | hypothetical protein | 45 | No hits in other organism | NA | 31.91 |
| TTHERM_00420520 | hypothetical protein | 70 | No hits in other organism | NA | 31.91 |
| TTHERM_00829500 | hypothetical protein | 59 | No hits in other organism | NA | 31.88 |
| TTHERM_00104980 | hypothetical protein | 49 | No hits in other organism | NA | 31.87 |
| TTHERM_00391550 | hypothetical protein | 85 | No hits in other organism | NA | 31.84 |
| TTHERM_00157840 | hypothetical protein | 52 | No hits in other organism | NA | 31.82 |
| TTHERM_00763070 | hypothetical protein | 69 | No hits in other organism | NA | 31.81 |
| TTHERM_00993020 | hypothetical protein | 52 | No hits in other organism | NA | 31.81 |
| TTHERM_00274410 | hypothetical protein | 42 | No hits in other organism | NA | 31.76 |
| TTHERM_00483460 | hypothetical protein | 44 | No hits in other organism | NA | 31.73 |
| TTHERM_01718410 | hypothetical protein | 66 | No hits in other organism | NA | 31.71 |
| TTHERM_00205220 | hypothetical protein | 113 | No hits in other organism | NA | 31.67 |
| TTHERM_00833630 | hypothetical protein | 80 | No hits in other organism | NA | 31.67 |
| TTHERM_00782130 | hypothetical protein | 62 | No hits in other organism | NA | 31.67 |
| TTHERM_00120810 | hypothetical protein | 99 | No hits in other organism | NA | 31.66 |
| TTHERM_00419700 | hypothetical protein | 53 | No hits in other organism | NA | 31.65 |
| TTHERM_00191980 | hypothetical protein | 56 | No hits in other organism | NA | 31.64 |
| TTHERM_00784610 | hypothetical protein | 47 | No hits in other organism | NA | 31.64 |
| TTHERM_00554410 | hypothetical protein | 46 | No hits in other organism | NA | 31.62 |
| TTHERM_00442040 | hypothetical protein | 42 | No hits in other organism | NA | 31.61 |
| TTHERM_00856710 | hypothetical protein | 113 | No hits in other organism | NA | 31.6 |
| TTHERM_01445930 | hypothetical protein | 103 | No hits in other organism | NA | 31.59 |
| TTHERM_00149270 | hypothetical protein | 43 | No hits in other organism | NA | 31.57 |
| TTHERM_00630210 | hypothetical protein | 64 | No hits in other organism | NA | 31.53 |
| TTHERM_01206510 | hypothetical protein | 224 | No hits in other organism | NA | 31.5 |
| TTHERM_01203220 | hypothetical protein | 58 | No hits in other organism | NA | 31.5 |
| TTHERM_01280580 | hypothetical protein | 48 | No hits in other organism | NA | 31.48 |
| TTHERM_00746840 | hypothetical protein | 60 | No hits in other organism | NA | 31.45 |
| TTHERM_01005180 | hypothetical protein | 53 | No hits in other organism | NA | 31.43 |
| TTHERM_01582410 | hypothetical protein | 43 | No hits in other organism | NA | 31.42 |
| TTHERM_00961890 | hypothetical protein | 45 | No hits in other organism | NA | 31.4 |
| TTHERM_00474390 | hypothetical protein | 64 | No hits in other organism | NA | 31.38 |
| TTHERM_01530560 | hypothetical protein | 41 | No hits in other organism | NA | 31.31 |
| TTHERM_00160940 | hypothetical protein | 71 | No hits in other organism | NA | 31.28 |
| TTHERM_00559820 | hypothetical protein | 52 | No hits in other organism | NA | 31.27 |
| TTHERM_00086680 | hypothetical protein | 40 | No hits in other organism | NA | 31.25 |
| TTHERM_01414140 | hypothetical protein | 56 | No hits in other organism | NA | 31.24 |
| TTHERM_01280650 | hypothetical protein | 45 | No hits in other organism | NA | 31.22 |
| TTHERM_00036900 | hypothetical protein | 50 | No hits in other organism | NA | 31.21 |
| TTHERM_00660370 | hypothetical protein | 58 | No hits in other organism | NA | 31.14 |
| TTHERM_00675600 | hypothetical protein | 42 | No hits in other organism | NA | 31.14 |
| TTHERM_00105500 | hypothetical protein | 41 | No hits in other organism | NA | 31.13 |
| TTHERM_00488410 | hypothetical protein | 60 | No hits in other organism | NA | 31.13 |
| TTHERM_00489520 | hypothetical protein | 130 | No hits in other organism | NA | 31.08 |
| TTHERM_00365400 | hypothetical protein | 45 | No hits in other organism | NA | 31.06 |
| TTHERM_00895930 | hypothetical protein | 83 | No hits in other organism | NA | 31.06 |
| TTHERM_00925330 | hypothetical protein | 78 | No hits in other organism | NA | 31.02 |
| TTHERM_00326760 | hypothetical protein | 58 | No hits in other organism | NA | 30.96 |
| TTHERM_00745830 | hypothetical protein | 68 | No hits in other organism | NA | 30.96 |
| TTHERM_00052230 | hypothetical protein | 77 | No hits in other organism | NA | 30.9 |
| TTHERM_00578770 | hypothetical protein | 67 | No hits in other organism | NA | 30.86 |
| TTHERM_00251200 | hypothetical protein | 57 | No hits in other organism | NA | 30.84 |
| TTHERM_01498870 | hypothetical protein | 68 | No hits in other organism | NA | 30.75 |
| TTHERM_00609510 | hypothetical protein | 82 | No hits in other organism | NA | 30.73 |
| TTHERM_00245600 | hypothetical protein | 66 | No hits in other organism | NA | 30.65 |
| TTHERM_00129310 | hypothetical protein | 66 | No hits in other organism | NA | 30.59 |
| TTHERM_00361340 | hypothetical protein | 150 | No hits in other organism | NA | 30.57 |
| TTHERM_01673200 | hypothetical protein | 70 | No hits in other organism | NA | 30.56 |
| TTHERM_01365660 | hypothetical protein | 76 | No hits in other organism | NA | 30.52 |
| TTHERM_00256820 | hypothetical protein | 50 | No hits in other organism | NA | 30.52 |
| TTHERM_00016210 | hypothetical protein | 117 | No hits in other organism | NA | 30.5 |
| TTHERM_00821900 | hypothetical protein | 102 | No hits in other organism | NA | 30.48 |
| TTHERM_00149140 | hypothetical protein | 133 | No hits in other organism | NA | 30.46 |
| TTHERM_00644620 | hypothetical protein | 49 | No hits in other organism | NA | 30.43 |
| TTHERM_01435700 | hypothetical protein | 53 | No hits in other organism | NA | 30.43 |
| TTHERM_00649200 | hypothetical protein | 56 | No hits in other organism | NA | 30.4 |
| TTHERM_00161080 | hypothetical protein | 62 | No hits in other organism | NA | 30.38 |
| TTHERM_00245860 | hypothetical protein | 167 | No hits in other organism | NA | 30.37 |
| TTHERM_00188430 | hypothetical protein | 52 | No hits in other organism | NA | 30.28 |
| TTHERM_00762970 | hypothetical protein | 55 | No hits in other organism | NA | 30.26 |
| TTHERM_00989360 | hypothetical protein | 53 | No hits in other organism | NA | 30.25 |
| TTHERM_00138270 | hypothetical protein | 41 | No hits in other organism | NA | 30.24 |
| TTHERM_00878230 | hypothetical protein | 84 | No hits in other organism | NA | 30.24 |
| TTHERM_00085410 | hypothetical protein | 71 | No hits in other organism | NA | 30.23 |
| TTHERM_00653750 | hypothetical protein | 76 | No hits in other organism | NA | 30.21 |
| TTHERM_00854220 | hypothetical protein | 97 | No hits in other organism | NA | 30.19 |
| TTHERM_00753680 | hypothetical protein | 54 | No hits in other organism | NA | 30.16 |
| TTHERM_00261780 | hypothetical protein | 67 | No hits in other organism | NA | 30.15 |
| TTHERM_00627040 | hypothetical protein | 245 | No hits in other organism | NA | 30.1 |
| TTHERM_00648600 | hypothetical protein | 131 | No hits in other organism | NA | 30.08 |
| TTHERM_00592730 | hypothetical protein | 81 | No hits in other organism | NA | 30.06 |
| TTHERM_00608310 | hypothetical protein | 148 | No hits in other organism | NA | 30.05 |
| TTHERM_00670880 | hypothetical protein | 52 | No hits in other organism | NA | 30.03 |
| TTHERM_01254470 | hypothetical protein | 105 | No hits in other organism | NA | 30 |
| TTHERM_00833820 | hypothetical protein | 452 | No hits in other organism | NA | 29.94 |
| TTHERM_00639960 | hypothetical protein | 50 | No hits in other organism | NA | 29.93 |
| TTHERM_00310680 | hypothetical protein | 69 | No hits in other organism | NA | 29.92 |
| TTHERM_00439290 | hypothetical protein | 83 | No hits in other organism | NA | 29.92 |
| TTHERM_00879260 | hypothetical protein | 42 | No hits in other organism | NA | 29.92 |
| TTHERM_01187300 | hypothetical protein | 83 | No hits in other organism | NA | 29.88 |
| TTHERM_00818440 | hypothetical protein | 73 | No hits in other organism | NA | 29.83 |
| TTHERM_00218700 | hypothetical protein | 45 | No hits in other organism | NA | 29.82 |
| TTHERM_01273250 | hypothetical protein | 52 | No hits in other organism | NA | 29.79 |
| TTHERM_00185440 | hypothetical protein | 58 | No hits in other organism | NA | 29.78 |
| TTHERM_00129910 | hypothetical protein | 44 | No hits in other organism | NA | 29.68 |
| TTHERM_01521370 | hypothetical protein | 61 | No hits in other organism | NA | 29.66 |
| TTHERM_00635680 | hypothetical protein | 52 | No hits in other organism | NA | 29.61 |
| TTHERM_00743700 | hypothetical protein | 98 | No hits in other organism | NA | 29.6 |
| TTHERM_00037790 | hypothetical protein | 56 | No hits in other organism | NA | 29.6 |
| TTHERM_01341650 | hypothetical protein | 56 | No hits in other organism | NA | 29.49 |
| TTHERM_01343660 | hypothetical protein | 96 | No hits in other organism | NA | 29.49 |
| TTHERM_01135030 | hypothetical protein | 53 | No hits in other organism | NA | 29.41 |
| TTHERM_01087750 | hypothetical protein | 53 | No hits in other organism | NA | 29.37 |
| TTHERM_00418630 | hypothetical protein | 43 | No hits in other organism | NA | 29.34 |
| TTHERM_00954070 | hypothetical protein | 53 | No hits in other organism | NA | 29.32 |
| TTHERM_01050670 | hypothetical protein | 45 | No hits in other organism | NA | 29.31 |
| TTHERM_00070810 | hypothetical protein | 46 | No hits in other organism | NA | 29.23 |
| TTHERM_00641380 | hypothetical protein | 69 | No hits in other organism | NA | 29.22 |
| TTHERM_00378720 | hypothetical protein | 89 | No hits in other organism | NA | 29.15 |
| TTHERM_00377410 | hypothetical protein | 48 | No hits in other organism | NA | 29.09 |
| TTHERM_01432570 | hypothetical protein | 55 | No hits in other organism | NA | 29.07 |
| TTHERM_01227880 | hypothetical protein | 45 | No hits in other organism | NA | 29.04 |
| TTHERM_00907100 | hypothetical protein | 54 | No hits in other organism | NA | 29.03 |
| TTHERM_00957520 | hypothetical protein | 110 | No hits in other organism | NA | 28.93 |
| TTHERM_00340110 | hypothetical protein | 171 | No hits in other organism | NA | 28.86 |
| TTHERM_00201770 | hypothetical protein | 53 | No hits in other organism | NA | 28.81 |
| TTHERM_00540370 | hypothetical protein | 51 | No hits in other organism | NA | 28.79 |
| TTHERM_00600580 | hypothetical protein | 62 | No hits in other organism | NA | 28.7 |
| TTHERM_01266140 | hypothetical protein | 86 | No hits in other organism | NA | 28.63 |
| TTHERM_00703290 | hypothetical protein | 57 | No hits in other organism | NA | 28.52 |
| TTHERM_00833860 | hypothetical protein | 45 | No hits in other organism | NA | 28.51 |
| TTHERM_01651030 | hypothetical protein | 54 | No hits in other organism | NA | 28.49 |
| TTHERM_00486920 | hypothetical protein | 50 | No hits in other organism | NA | 28.47 |
| TTHERM_01127490 | hypothetical protein | 44 | No hits in other organism | NA | 28.43 |
| TTHERM_00609430 | hypothetical protein | 41 | No hits in other organism | NA | 28.41 |
| TTHERM_00263060 | hypothetical protein | 58 | No hits in other organism | NA | 28.39 |
| TTHERM_01081750 | hypothetical protein | 44 | No hits in other organism | NA | 28.37 |
| TTHERM_00343340 | hypothetical protein | 96 | No hits in other organism | NA | 28.33 |
| TTHERM_00756390 | hypothetical protein | 71 | No hits in other organism | NA | 28.3 |
| TTHERM_00638920 | hypothetical protein | 51 | No hits in other organism | NA | 28.26 |
| TTHERM_01677230 | hypothetical protein | 35 | No hits in other organism | NA | 28.2 |
| TTHERM_00942670 | hypothetical protein | 151 | No hits in other organism | NA | 28.19 |
| TTHERM_01018390 | hypothetical protein | 42 | No hits in other organism | NA | 28.19 |
| TTHERM_00300680 | hypothetical protein | 42 | No hits in other organism | NA | 28.13 |
| TTHERM_00013680 | hypothetical protein | 47 | No hits in other organism | NA | 28.07 |
| TTHERM_01101640 | hypothetical protein | 69 | No hits in other organism | NA | 28.01 |
| TTHERM_00948670 | hypothetical protein | 59 | No hits in other organism | NA | 27.95 |
| TTHERM_00075740 | hypothetical protein | 101 | No hits in other organism | NA | 27.94 |
| TTHERM_00128600 | hypothetical protein | 57 | No hits in other organism | NA | 27.94 |
| TTHERM_00742690 | hypothetical protein | 69 | No hits in other organism | NA | 27.91 |
| TTHERM_00489530 | hypothetical protein | 190 | No hits in other organism | NA | 27.84 |
| TTHERM_00616670 | hypothetical protein | 41 | No hits in other organism | NA | 27.62 |
| TTHERM_01315360 | hypothetical protein | 48 | No hits in other organism | NA | 27.51 |
| TTHERM_00415710 | hypothetical protein | 72 | No hits in other organism | NA | 27.42 |
| TTHERM_00390020 | hypothetical protein | 53 | No hits in other organism | NA | 27.09 |
| TTHERM_00047610 | hypothetical protein | 44 | No hits in other organism | NA | 27.05 |
| TTHERM_00561620 | hypothetical protein | 60 | No hits in other organism | NA | 27.01 |
| TTHERM_01195970 | hypothetical protein | 45 | No hits in other organism | NA | 26.98 |
| TTHERM_01088010 | hypothetical protein | 74 | No hits in other organism | NA | 26.93 |
| TTHERM_00131230 | hypothetical protein | 53 | No hits in other organism | NA | 26.67 |
| TTHERM_00035790 | hypothetical protein | 97 | No hits in other organism | NA | 26.36 |
| TTHERM_01308020 | hypothetical protein | 43 | No hits in other organism | NA | 26.14 |
|  |  |  |  |  |  |
| **Class 4. Genes without designed microarray probes** | | |  |  |  |
| TTHERM_00655399 | hypothetical protein | 370 | No hits in other organism | NA | NA |
| TTHERM_00214839 | hypothetical protein | 209 | No hits in other organism | NA | NA |
| TTHERM_00582398 | conserved hypothetical protein | 687 | No hits in other organism | NA | NA |
| TTHERM_00425849 | hypothetical protein | 179 | No hits in other organism | NA | NA |
| TTHERM_00343595 | hypothetical protein | 175 | No hits in other organism | NA | NA |
| TTHERM_00321659 | hypothetical protein | 158 | No hits in other organism | NA | NA |
| TTHERM_00420809 | hypothetical protein | 133 | No hits in other organism | NA | NA |
| TTHERM_00289039 | hypothetical protein | 128 | No hits in other organism | NA | NA |
| TTHERM_00585009 | hypothetical protein | 123 | No hits in other organism | NA | NA |
| TTHERM_00499689 | hypothetical protein | 121 | No hits in other organism | NA | NA |
| TTHERM_00190729 | hypothetical protein | 118 | No hits in other organism | NA | NA |
| TTHERM_01051850 | hypothetical protein | 96 | No hits in other organism | NA | NA |
| TTHERM_01786780 | hypothetical protein | 74 | No hits in other organism | NA | NA |
| TTHERM_01722450 | hypothetical protein | 68 | No hits in other organism | NA | NA |
| TTHERM_01258660 | hypothetical protein | 55 | No hits in other organism | NA | NA |
| TTHERM_01760640 | hypothetical protein | 53 | No hits in other organism | NA | NA |
| TTHERM_00105080 | hypothetical protein | 52 | No hits in other organism | NA | NA |
| TTHERM_01033290 | hypothetical protein | 52 | No hits in other organism | NA | NA |
| TTHERM_00683200 | hypothetical protein | 51 | No hits in other organism | NA | NA |
| TTHERM_01100550 | hypothetical protein | 51 | No hits in other organism | NA | NA |
| TTHERM_00000080 | hypothetical protein | 50 | No hits in other organism | NA | NA |
| TTHERM_00673450 | hypothetical protein | 50 | No hits in other organism | NA | NA |
| TTHERM_01115250 | hypothetical protein | 49 | No hits in other organism | NA | NA |
| TTHERM_00487160 | hypothetical protein | 48 | No hits in other organism | NA | NA |
| TTHERM_01006410 | hypothetical protein | 48 | No hits in other organism | NA | NA |
| TTHERM_01271230 | hypothetical protein | 48 | No hits in other organism | NA | NA |
| TTHERM_00263190 | hypothetical protein | 47 | No hits in other organism | NA | NA |
| TTHERM_00976530 | hypothetical protein | 47 | No hits in other organism | NA | NA |
| TTHERM_00463670 | hypothetical protein | 46 | No hits in other organism | NA | NA |
| TTHERM_00463920 | hypothetical protein | 45 | No hits in other organism | NA | NA |
| TTHERM_01050710 | hypothetical protein | 45 | No hits in other organism | NA | NA |
| TTHERM_01737570 | hypothetical protein | 45 | No hits in other organism | NA | NA |
| TTHERM_00227890 | hypothetical protein | 44 | No hits in other organism | NA | NA |
| TTHERM_00571950 | hypothetical protein | 43 | No hits in other organism | NA | NA |
| TTHERM_01406930 | hypothetical protein | 43 | No hits in other organism | NA | NA |
| TTHERM_00122490 | hypothetical protein | 42 | No hits in other organism | NA | NA |
| TTHERM_00795680 | hypothetical protein | 42 | No hits in other organism | NA | NA |
| TTHERM_00741620 | hypothetical protein | 41 | No hits in other organism | NA | NA |
| TTHERM_00776000 | hypothetical protein | 41 | No hits in other organism | NA | NA |
| TTHERM_01253390 | hypothetical protein | 41 | No hits in other organism | NA | NA |
| TTHERM_00592850 | hypothetical protein | 40 | No hits in other organism | NA | NA |
| TTHERM_00697590 | hypothetical protein | 40 | No hits in other organism | NA | NA |
| TTHERM_01213970 | hypothetical protein | 40 | No hits in other organism | NA | NA |
| TTHERM_01406940 | hypothetical protein | 39 | No hits in other organism | NA | NA |
| TTHERM_01173650 | hypothetical protein | 37 | No hits in other organism | NA | NA |
| TTHERM_01032290 | hypothetical protein | 36 | No hits in other organism | NA | NA |
| TTHERM_01560040 | hypothetical protein | 36 | No hits in other organism | NA | NA |
| TTHERM_01132950 | hypothetical protein | 34 | No hits in other organism | NA | NA |
| TTHERM_01693310 | hypothetical protein | 34 | No hits in other organism | NA | NA |
| TTHERM_00699860 | hypothetical protein | 32 | No hits in other organism | NA | NA |
| TTHERM_01024120 | hypothetical protein | 32 | No hits in other organism | NA | NA |
| TTHERM_00858140 | hypothetical protein | 30 | No hits in other organism | NA | NA |
| TTHERM_01340600 | hypothetical protein | 29 | No hits in other organism | NA | NA |
| TTHERM_00203070 | hypothetical protein | 26 | No hits in other organism | NA | NA |
| TTHERM_01242410 | hypothetical protein | 26 | No hits in other organism | NA | NA |
| TTHERM_00817300 | hypothetical protein | 25 | No hits in other organism | NA | NA |
| TTHERM_00828290 | hypothetical protein | 25 | No hits in other organism | NA | NA |
| TTHERM_01170570 | hypothetical protein | 24 | No hits in other organism | NA | NA |
| TTHERM_00744730 | hypothetical protein | 23 | No hits in other organism | NA | NA |
| TTHERM_01391280 | hypothetical protein | 23 | No hits in other organism | NA | NA |
| TTHERM_01563120 | hypothetical protein | 23 | No hits in other organism | NA | NA |
| TTHERM_01623890 | hypothetical protein | 23 | No hits in other organism | NA | NA |
| TTHERM_00422250 | hypothetical protein | 22 | No hits in other organism | NA | NA |
| TTHERM_01617810 | hypothetical protein | 22 | No hits in other organism | NA | NA |
| TTHERM_01702350 | hypothetical protein | 22 | No hits in other organism | NA | NA |
| TTHERM_00381100 | hypothetical protein | 20 | No hits in other organism | NA | NA |
| TTHERM_00363260 | hypothetical protein | 19 | No hits in other organism | NA | NA |
| TTHERM_00810540 | hypothetical protein | 19 | No hits in other organism | NA | NA |
| TTHERM_01425460 | hypothetical protein | 17 | No hits in other organism | NA | NA |
| TTHERM_00022850 | hypothetical protein | 14 | No hits in other organism | NA | NA |
| TTHERM_01116250 | hypothetical protein | 14 | No hits in other organism | NA | NA |

NA: Not Available
