# Supplementary material for: Transcriptome Analysis of the Model Protozoan, Tetrahymena thermophila, Using Deep RNA Sequencing
Source: PLoS One. 2012 Feb 7;7(2):e30630. doi: 10.1371/journal.pone.0030630 (PMC3274533; doi:10.1371/journal.pone.0030630)
Supplement: Table S3 — Comparisons of intron base compositions. (DOC) [file pone.0030630.s006.doc]

**Table S3. Comparisons of intron base compositions.**

**A. Comparison of base compositions of introns as predicted by RNA-seq and in the latest gene predictions.**

| **Introns** | **Base A** | **Base T** | **Base C** | **Base G** |
| --- | --- | --- | --- | --- |
| All predicted introns | 0.4189 | 0.4177 | 0.0816 | 0.0818 |
| All RNA-Seq identified introns | 0.3955 | 0.3992 | 0.1019 | 0.1034 |

The base composition differences for the two types of introns are significant as determined by chi-square test (P < 0.01)

B. Comparison of base composition of RNA-seq predicted AS introns and all introns.

| **AS type** | **Base A** | **Base T** | **Base C** | **Base G** |
| --- | --- | --- | --- | --- |
| Alternative 3’ splice-site selection | 0.4016 | 0.4045 | 0.0962 | 0.0977 |
| Alternative 5’ splice-site selection | 0.4077 | 0.4007 | 0.0995 | 0.0921 |
| Cassette-exon inclusion or skipping | 0.3972 | 0.4057 | 0.0986 | 0.0985 |
| Intron retention | 0.4009 | 0.4053 | 0.0955 | 0.0983 |
| All RNA-Seq identified introns | 0.3955 | 0.3992 | 0.1019 | 0.1034 |

Statistical significance was determined by chi-square test. The base composition differences among the four types of AS introns are not significant but the difference between all AS introns and all introns is significant (P < 0.01).
