# Supplementary material for: Transcriptome Analysis of the Model Protozoan, Tetrahymena thermophila, Using Deep RNA Sequencing
Source: PLoS One. 2012 Feb 7;7(2):e30630. doi: 10.1371/journal.pone.0030630 (PMC3274533; doi:10.1371/journal.pone.0030630)
Supplement: Table S4 — 217 novel transcribed regions annotated in previous genome annotation but lost in current genome annotation. (DOC) [file pone.0030630.s007.doc]

**Table S4. 217 novel transcribed regions annotated in previous genome annotation but lost in current genome annotation.**

| **Novel region** | **Corresponding gene in previous genome annotation** |
| --- | --- |
| scaffold_1:1995373..2000788 | TTHERM_00022830 |
| scaffold_121:120818..121850 | TTHERM_00745790 |
| scaffold_121:159291..159652 | TTHERM_00746860 |
| scaffold_139:178479..178915 | TTHERM_00820630 |
| scaffold_144:793..2101 | TTHERM_00828280 |
| scaffold_145:173331..173930 | TTHERM_00835450 |
| scaffold_145:693..2903 | TTHERM_00834880 |
| scaffold_151:103459..104655 | TTHERM_00856450 |
| scaffold_151:105426..105862 | TTHERM_00856450 |
| scaffold_151:106650..107079 | TTHERM_00856450 |
| scaffold_151:109429..109771 | TTHERM_00856450 |
| scaffold_151:165402..166839 | TTHERM_00856630 |
| scaffold_151:93369..94928 | TTHERM_00856450 |
| scaffold_161:11008..11479 | TTHERM_00900620 |
| scaffold_161:13112..15587 | TTHERM_00900620 |
| scaffold_161:136528..137171 | TTHERM_00903940 |
| scaffold_172:6230..6917 | TTHERM_00942620 |
| scaffold_177:126655..127789 | TTHERM_00958690 |
| scaffold_177:70320..71824 | TTHERM_00956510 |
| scaffold_179:28808..29145 | TTHERM_00963270 |
| scaffold_179:30333..32846 | TTHERM_00963270 |
| scaffold_181:144124..145107 | TTHERM_00975370 |
| scaffold_181:145368..148812 | TTHERM_00975370 |
| scaffold_182:9192..11055 | TTHERM_00976440 |
| scaffold_199:120741..122202 | TTHERM_01018520 |
| scaffold_201:60712..61032 | TTHERM_01023020 |
| scaffold_214:25798..26696 | TTHERM_01054170 |
| scaffold_243:45054..46221 | TTHERM_01122660 |
| scaffold_257:12251..14182 | TTHERM_01153610 |
| scaffold_257:9041..10531 | TTHERM_01153610 |
| scaffold_263:10796..12573 | TTHERM_01170560 |
| scaffold_263:10796..12573 | TTHERM_01170560 |
| scaffold_263:11831..15344 | TTHERM_01170560 |
| scaffold_263:14089..14542 | TTHERM_01170560 |
| scaffold_263:15457..16003 | TTHERM_01170560 |
| scaffold_279:10328..15388 | TTHERM_01220310 |
| scaffold_279:8105..9898 | TTHERM_01220310 |
| scaffold_281:54644..55539 | TTHERM_01225680 |
| scaffold_283:25803..26444 | TTHERM_01228950 |
| scaffold_283:32052..32812 | TTHERM_01228950 |
| scaffold_285:47151..48207 | TTHERM_01232280 |
| scaffold_285:52238..53593 | TTHERM_01232280 |
| scaffold_302:33320..34369 | TTHERM_01270200 |
| scaffold_317:32968..34626 | TTHERM_01306880 |
| scaffold_336:1363..4032 | TTHERM_01344670 |
| scaffold_336:5506..9636 | TTHERM_01344670 |
| scaffold_336:5506..9636 | TTHERM_01344670 |
| scaffold_351:18276..19476 | TTHERM_01369750 |
| scaffold_351:19746..21213 | TTHERM_01369750 |
| scaffold_360:24373..25134 | TTHERM_01389250 |
| scaffold_3677:150187..150853 | TTHERM_00648960 |
| scaffold_368:6164..6474 | TTHERM_01400680 |
| scaffold_3680:495736..499798 | TTHERM_00487120 |
| scaffold_3689:275772..281727 | TTHERM_00336010 |
| scaffold_3691:204839..205863 | TTHERM_00299710 |
| scaffold_3691:205967..209091 | TTHERM_00299710 |
| scaffold_3696:434234..435101 | TTHERM_00310060 |
| scaffold_3696:70781..71653 | TTHERM_00304280 |
| scaffold_3698:995364..997799 | 3698.m01709 |
| scaffold_3698:995364..997799 | 3698.m01709 |
| scaffold_3713:309320..311534 | TTHERM_00401890 |
| scaffold_3719:383560..384208 | TTHERM_01602640 |
| scaffold_3719:386319..390126 | TTHERM_01601640 |
| scaffold_3719:390428..393014 | TTHERM_01601640 |
| scaffold_372:20646..21585 | TTHERM_01407970 |
| scaffold_3723:48493..51613 | TTHERM_00503900 |
| scaffold_3724:342328..344418 | TTHERM_00659110 |
| scaffold_3727:18147..19548 | TTHERM_00646800 |
| scaffold_3736:309222..310999 | TTHERM_00660240 |
| scaffold_3749:418..3723 | TTHERM_00968570 |
| scaffold_3750:135085..136141 | TTHERM_00849520 |
| scaffold_3750:137038..137494 | TTHERM_00849520 |
| scaffold_3750:138801..146883 | TTHERM_00849520 |
| scaffold_3752:184401..185091 | TTHERM_00928400 |
| scaffold_3753:172345..173218 | TTHERM_00693300 |
| scaffold_3753:173886..174887 | TTHERM_00693300 |
| scaffold_3753:175091..177402 | TTHERM_00693300 |
| scaffold_3755:56918..67480 | TTHERM_00785910 |
| scaffold_3757:662..3729 | TTHERM_01003920 |
| scaffold_376:3670..4341 | TTHERM_01415150 |
| scaffold_38:562967..563697 | TTHERM_00366540 |
| scaffold_3808:114212..120939 | TTHERM_00802530 |
| scaffold_3808:190024..192462 | TTHERM_00803710 |
| scaffold_3808:21253..21956 | TTHERM_00800260 |
| scaffold_3808:22069..22540 | TTHERM_00800260 |
| scaffold_3808:22662..23404 | TTHERM_00800260 |
| scaffold_3810:944460..945663 | TTHERM_00570560 |
| scaffold_3810:956352..958131 | TTHERM_00569540 |
| scaffold_3811:400405..403126 | TTHERM_00449760 |
| scaffold_3812:1072535..1073089 | TTHERM_00118780 |
| scaffold_3812:1074318..1074848 | TTHERM_00118780 |
| scaffold_3812:1079082..1079700 | TTHERM_00119780 |
| scaffold_3812:453299..455074 | TTHERM_00109290 |
| scaffold_3812:455581..456693 | TTHERM_00109290 |
| scaffold_3813:229052..229683 | TTHERM_00393410 |
| scaffold_3813:512227..512675 | TTHERM_01711380 |
| scaffold_3813:525789..526461 | TTHERM_00391450 |
| scaffold_3813:797030..797385 | TTHERM_02643280 |
| scaffold_3815:32920..38740 | TTHERM_01092320 |
| scaffold_3815:32920..38740 | TTHERM_01092320 |
| scaffold_3820:26244..29325 | TTHERM_01000220 |
| scaffold_3822:214632..216249 | TTHERM_00628310 |
| scaffold_3822:360613..361912 | TTHERM_00625980 |
| scaffold_3822:424133..425361 | TTHERM_01212900 |
| scaffold_3823:570288..571556 | TTHERM_01215050 |
| scaffold_3823:672721..674794 | TTHERM_01162050 |
| scaffold_3825:1006225..1006910 | TTHERM_00033910 |
| scaffold_3825:1792612..1795376 | 2.m10686 |
| scaffold_3825:1792612..1795376 | 2.m10686 |
| scaffold_3825:1988256..1990396 | TTHERM_00039310 |
| scaffold_3825:287980..288552 | TTHERM_00652620 |
| scaffold_3825:767..3319 | TTHERM_00654270 |
| scaffold_3826:1539..14326 | TTHERM_01151430 |
| scaffold_3828:1216002..1216853 | 5.m05447 |
| scaffold_3828:336..3099 | TTHERM_00072130 |
| scaffold_3828:46667..47388 | TTHERM_00073240 |
| scaffold_3828:48621..49382 | TTHERM_00073240 |
| scaffold_3828:49727..50424 | TTHERM_00073240 |
| scaffold_3828:53184..53786 | TTHERM_00073240 |
| scaffold_3828:54596..55716 | TTHERM_00073240 |
| scaffold_3828:7388..8148 | TTHERM_00072130 |
| scaffold_3828:791414..796165 | TTHERM_00078980 |
| scaffold_3828:791414..796165 | TTHERM_00078980 |
| scaffold_3829:1010484..1016167 | TTHERM_01418330 |
| scaffold_3829:39539..44250 | TTHERM_00161770 |
| scaffold_3829:39539..44250 | TTHERM_00161770 |
| scaffold_3829:571999..572593 | 13.m04865 |
| scaffold_3829:968547..973159 | TTHERM_02366040 |
| scaffold_3830:400474..402050 | TTHERM_00532890 |
| scaffold_3831:1151739..1154413 | TTHERM_02168690 |
| scaffold_3831:374421..376037 | TTHERM_00629740 |
| scaffold_3831:458708..459061 | TTHERM_01178650 |
| scaffold_3831:458708..459061 | TTHERM_01178650 |
| scaffold_3831:499209..501202 | TTHERM_01089080 |
| scaffold_3831:501330..502176 | TTHERM_01089080 |
| scaffold_3831:502478..503720 | TTHERM_01089080 |
| scaffold_3831:503851..504346 | TTHERM_01089080 |
| scaffold_3831:504711..506239 | TTHERM_01089080 |
| scaffold_3833:319890..321246 | TTHERM_01510190 |
| scaffold_3833:422188..424366 | TTHERM_00414540 |
| scaffold_3833:424983..427146 | TTHERM_00414540 |
| scaffold_3833:808052..810710 | TTHERM_00886990 |
| scaffold_3835:132891..133858 | TTHERM_00593080 |
| scaffold_3835:254303..255386 | TTHERM_00595430 |
| scaffold_3835:380006..381029 | TTHERM_00597660 |
| scaffold_3835:381790..382582 | TTHERM_00597660 |
| scaffold_3835:382853..383802 | TTHERM_00597660 |
| scaffold_3835:384068..384592 | TTHERM_00597660 |
| scaffold_3836:1176268..1177270 | TTHERM_00082280 |
| scaffold_3836:146374..152688 | TTHERM_00092930 |
| scaffold_3836:333572..336256 | TTHERM_00090440 |
| scaffold_3836:335419..337056 | TTHERM_00090440 |
| scaffold_3836:336394..337056 | TTHERM_00090440 |
| scaffold_3836:493237..496081 | TTHERM_00087010 |
| scaffold_3836:505027..506118 | TTHERM_00086980 |
| scaffold_3836:505062..516253 | TTHERM_00086980 |
| scaffold_3836:519924..520712 | TTHERM_00086980 |
| scaffold_3836:523805..524330 | TTHERM_00086980 |
| scaffold_3836:525066..525946 | TTHERM_00086980 |
| scaffold_422:3862..5040 | TTHERM_01496780 |
| scaffold_422:5384..6421 | TTHERM_01496780 |
| scaffold_422:8775..9263 | TTHERM_01496780 |
| scaffold_441:413..5981 | TTHERM_01523400 |
| scaffold_441:413..5981 | TTHERM_01523400 |
| scaffold_472:1399..6168 | TTHERM_01569160 |
| scaffold_472:6739..7744 | TTHERM_01569160 |
| scaffold_480:372..896 | TTHERM_01579300 |
| scaffold_488:4810..5480 | TTHERM_01588500 |
| scaffold_488:4810..5922 | TTHERM_01588500 |
| scaffold_488:6330..6957 | TTHERM_01588500 |
| scaffold_541:2959..3813 | TTHERM_01662080 |
| scaffold_642:326..742 | TTHERM_01769690 |
| scaffold_726:52..410 | TTHERM_01851020 |
| scaffold_84:320823..322871 | TTHERM_00610550 |
| scaffold_3825:2165656..2166859 | TTHERM_01682250 |
| scaffold_368:3016..3374 | TTHERM_01400680 |
| scaffold_306:4398..5174 | TTHERM_01279550 |
| scaffold_3829:739218..742008 | TTHERM_00157860 |
| scaffold_3735:283839..284421 | TTHERM_00558340 |
| scaffold_3829:782921..783591 | TTHERM_00156730 |
| scaffold_99:189314..190014 | TTHERM_00666620 |
| scaffold_19:856401..857364 | TTHERM_00235220 |
| scaffold_99:120229..121770 | 99.m01410 |
| scaffold_3813:38979..39965 | TTHERM_00395960 |
| scaffold_3672:171541..175016 | TTHERM_00760640 |
| scaffold_207:117178..120469 | TTHERM_01042040 |
| scaffold_3823:291588..292339 | TTHERM_00463200 |
| scaffold_1:1762366..1763851 | TTHERM_00016190 |
| scaffold_3687:363371..364785 | TTHERM_00316890 |
| scaffold_3824:1497530..1499580 | TTHERM_00052570 |
| scaffold_3707:44770..46395 | TTHERM_00071040 |
| scaffold_3811:403258..406049 | TTHERM_00449760 |
| scaffold_101:332608..333536 | TTHERM_00675890 |
| scaffold_620:2050..2910 | TTHERM_01747600 |
| scaffold_1069:15..368 | TTHERM_02098570 |
| scaffold_3836:232968..233629 | TTHERM_00091760 |
| scaffold_90:192450..193033 | TTHERM_00633330 |
| scaffold_3696:265368..266033 | TTHERM_00307720 |
| scaffold_3834:202208..207432 | TTHERM_00852950 |
| scaffold_96:158032..158684 | TTHERM_00655690 |
| scaffold_487:4811..5827 | TTHERM_01587470 |
| scaffold_1:1762366..1763851 | TTHERM_00016190 |
| scaffold_3825:574479..575856 | TTHERM_00028910 |
| scaffold_3823:667796..669252 | TTHERM_01161050 |
| scaffold_3687:327124..328136 | TTHERM_00316810 |
| scaffold_187:148949..150054 | TTHERM_00995420 |
| scaffold_3699:387150..388037 | TTHERM_00128910 |
| scaffold_3707:219113..220581 | TTHERM_00069660 |
| scaffold_3697:653270..654114 | TTHERM_00191080 |
| scaffold_3831:946519..947138 | TTHERM_00430190 |
| scaffold_160:131382..132162 | TTHERM_00899440 |
| scaffold_375:5198..6185 | TTHERM_01414110 |
| scaffold_3811:403258..406049 | TTHERM_00449760 |
| scaffold_3696:427711..428364 | TTHERM_00310060 |
| scaffold_3825:2162855..2163657 | TTHERM_01682250 |
| scaffold_306:3288..3927 | TTHERM_01279550 |
| scaffold_3811:399055..399621 | TTHERM_00449760 |
