# Supplementary material for: Transcriptome Analysis of the Model Protozoan, Tetrahymena thermophila, Using Deep RNA Sequencing
Source: PLoS One. 2012 Feb 7;7(2):e30630. doi: 10.1371/journal.pone.0030630 (PMC3274533; doi:10.1371/journal.pone.0030630)
Supplement: Table S5 — The 745 novel transcribed regions giving blast hits with expected values less than 1e-3. (DOC) [file pone.0030630.s008.doc]

**Table S5. The 745 novel transcribed regions giving blast hits with expected values less than 1e-3.**

| **Novel region** | **Transcript length** | **Best BLAST hit** | **E-value** |
| --- | --- | --- | --- |
| scaffold_99:58942..59784 | 742 | Nop10 family nucleolar RNA-binding protein [Ajellomyces capsulatus H143] | 1.00E-13 |
| scaffold_99:120229..121770 | 1438 | hypothetical protein TTHERM_00666429 [Tetrahymena thermophila] | 4.00E-62 |
| scaffold_96:186004..186557 | 554 | hypothetical protein TTHERM_01595620 [Tetrahymena thermophila] | 3.00E-14 |
| scaffold_945:1187..1535 | 349 | hypothetical protein TTHERM_01658030 [Tetrahymena thermophila] | 5.00E-27 |
| scaffold_941:18..585 | 568 | hypothetical protein TTHERM_01246660 [Tetrahymena thermophila] | 1.00E-19 |
| scaffold_91:272685..273107 | 423 | hypothetical protein TTHERM_00625930 [Tetrahymena thermophila] | 5.00E-30 |
| scaffold_90:340451..340995 | 545 | hypothetical protein TTHERM_01341610 [Tetrahymena thermophila] | 2.00E-11 |
| scaffold_90:235029..235471 | 443 | hypothetical protein TTHERM_01062890 [Tetrahymena thermophila] | 5.00E-07 |
| scaffold_870:1243..1624 | 382 | hypothetical protein TTHERM_01234340 [Tetrahymena thermophila] | 3.00E-23 |
| scaffold_84:79195..80207 | 1013 | transporter, cation channel family [Tetrahymena thermophila] | 5.00E-07 |
| scaffold_84:383729..386205 | 758 | hypothetical protein TTHERM_00181030 [Tetrahymena thermophila] | 2.00E-28 |
| scaffold_84:320823..322871 | 715 | cyclic nucleotide-binding domain containing protein [Tetrahymena thermophila] | 1.00E-22 |
| scaffold_805:1218..1700 | 338 | hypothetical protein TTHERM_01587480 [Tetrahymena thermophila] | 2.00E-05 |
| scaffold_747:1510..2055 | 546 | hypothetical protein TTHERM_00316880 [Tetrahymena thermophila] | 6.00E-68 |
| scaffold_741:1282..1715 | 434 | hypothetical protein TTHERM_00990520 [Tetrahymena thermophila] | 1.00E-27 |
| scaffold_726:52..410 | 359 | hypothetical protein TTHERM_00990520 [Tetrahymena thermophila] | 1.00E-18 |
| scaffold_72:232712..233693 | 928 | hypothetical protein TTHERM_00554240 [Tetrahymena thermophila] | 2.00E-46 |
| scaffold_7:153279..155864 | 1916 | hypothetical protein TTHERM_00096769 [Tetrahymena thermophila] | 1.00E-97 |
| scaffold_689:1899..2555 | 355 | hypothetical protein TTHERM_00757730 [Tetrahymena thermophila] | 1.00E-04 |
| scaffold_642:326..742 | 417 | hypothetical protein TTHERM_02103570 [Tetrahymena thermophila] | 6.00E-13 |
| scaffold_633:469..888 | 420 | hypothetical protein TTHERM_00625930 [Tetrahymena thermophila] | 2.00E-25 |
| scaffold_633:1209..1810 | 602 | hypothetical protein TTHERM_00743700 [Tetrahymena thermophila] | 9.00E-09 |
| scaffold_615:59..439 | 381 | hypothetical protein TTHERM_01246660 [Tetrahymena thermophila] | 2.00E-23 |
| scaffold_606:1946..2676 | 538 | hypothetical protein EAG_01237 [Camponotus floridanus] | 8.00E-04 |
| scaffold_60:329050..330749 | 877 | conserved hypothetical protein [Tetrahymena thermophila] | 4.00E-15 |
| scaffold_60:277444..278835 | 598 | cyclic nucleotide-binding domain containing protein [Tetrahymena thermophila] | 8.00E-10 |
| scaffold_60:275347..277208 | 671 | hypothetical protein TTHERM_00714630 [Tetrahymena thermophila] | 1.00E-27 |
| scaffold_599:2791..3802 | 808 | hypothetical protein TTHERM_01484660 [Tetrahymena thermophila] | 1.00E-39 |
| scaffold_551:739..1104 | 366 | hypothetical protein TTHERM_00625930 [Tetrahymena thermophila] | 2.00E-22 |
| scaffold_541:2959..3813 | 623 | hypothetical protein TTHERM_01481610 [Tetrahymena thermophila] | 3.00E-20 |
| scaffold_536:974..2068 | 980 | hypothetical protein TTHERM_01480590 [Tetrahymena thermophila] | 3.00E-06 |
| scaffold_535:2068..2634 | 567 | hypothetical protein TTHERM_02579220 [Tetrahymena thermophila] | 9.00E-15 |
| scaffold_511:7529..8289 | 394 | hypothetical protein TTHERM_01366660 [Tetrahymena thermophila] | 1.00E-04 |
| scaffold_508:1939..2485 | 547 | hypothetical protein TTHERM_00625930 [Tetrahymena thermophila] | 4.00E-31 |
| scaffold_501:2569..3069 | 501 | hypothetical protein TTHERM_00625930 [Tetrahymena thermophila] | 7.00E-28 |
| scaffold_491:9146..9682 | 537 | hypothetical protein TTHERM_01598630 [Tetrahymena thermophila] | 9.00E-33 |
| scaffold_491:1462..1977 | 516 | hypothetical protein TTHERM_00990520 [Tetrahymena thermophila] | 1.00E-22 |
| scaffold_488:6330..6957 | 514 | hypothetical protein TTHERM_01246660 [Tetrahymena thermophila] | 3.00E-30 |
| scaffold_488:5532..5922 | 391 | hypothetical protein TTHERM_02455140 [Tetrahymena thermophila] | 1.00E-16 |
| scaffold_488:4810..5922 | 915 | hypothetical protein TTHERM_02455140 [Tetrahymena thermophila] | 6.00E-16 |
| scaffold_488:4810..5480 | 489 | hypothetical protein TTHERM_01562080 [Tetrahymena thermophila] | 4.00E-04 |
| scaffold_480:372..896 | 525 | hypothetical protein TTHERM_01017240 [Tetrahymena thermophila] | 2.00E-21 |
| scaffold_472:6739..7744 | 361 | Protein kinase domain containing protein [Tetrahymena thermophila] | 3.00E-35 |
| scaffold_472:1399..6168 | 2345 | Protein kinase domain containing protein [Tetrahymena thermophila] | 0 |
| scaffold_47:491303..492223 | 670 | hypothetical protein TTHERM_01595620 [Tetrahymena thermophila] | 2.00E-05 |
| scaffold_47:488742..489450 | 443 | hypothetical protein TTHERM_01222520 [Tetrahymena thermophila] | 3.00E-07 |
| scaffold_47:487607..488466 | 320 | hypothetical protein TTHERM_01674210 [Tetrahymena thermophila] | 7.00E-11 |
| scaffold_47:257850..258348 | 499 | hypothetical protein TTHERM_01017240 [Tetrahymena thermophila] | 3.00E-23 |
| scaffold_464:8681..12300 | 2357 | hypothetical protein TTHERM_01595620 [Tetrahymena thermophila] | 5.00E-29 |
| scaffold_462:12578..12988 | 411 | hypothetical protein TTHERM_01556000 [Tetrahymena thermophila] | 2.00E-14 |
| scaffold_461:30..596 | 341 | ADP-ribosylation factor 6, putative [Tetrahymena thermophila] | 4.00E-47 |
| scaffold_446:42..755 | 653 | hypothetical protein TTHERM_00975410 [Tetrahymena thermophila] | 3.00E-04 |
| scaffold_441:413..5981 | 2296 | hypothetical protein TTHERM_01551970 [Tetrahymena thermophila] | 8.00E-82 |
| scaffold_441:12513..16235 | 1342 | hypothetical protein TTHERM_01475520 [Tetrahymena thermophila] | 2.00E-45 |
| scaffold_440:15541..16158 | 618 | hypothetical protein TTHERM_01017240 [Tetrahymena thermophila] | 3.00E-30 |
| scaffold_439:9317..10135 | 819 | hypothetical protein TTHERM_01545800 [Tetrahymena thermophila] | 3.00E-54 |
| scaffold_436:8..1617 | 1376 | hypothetical protein TTHERM_00990520 [Tetrahymena thermophila] | 2.00E-36 |
| scaffold_434:4741..6510 | 618 | hypothetical protein TTHERM_01476520 [Tetrahymena thermophila] | 4.00E-14 |
| scaffold_430:12326..13712 | 384 | hypothetical protein TTHERM_01256640 [Tetrahymena thermophila] | 3.00E-17 |
| scaffold_430:10990..12121 | 343 | hypothetical protein TTHERM_00902820 [Tetrahymena thermophila] | 6.00E-15 |
| scaffold_427:384..1559 | 1050 | hypothetical protein TTHERM_00990520 [Tetrahymena thermophila] | 3.00E-32 |
| scaffold_427:11737..12178 | 442 | hypothetical protein TTHERM_00625930 [Tetrahymena thermophila] | 4.00E-39 |
| scaffold_427:10621..11124 | 504 | hypothetical protein TTHERM_01017240 [Tetrahymena thermophila] | 1.00E-25 |
| scaffold_422:8775..9263 | 413 | Protein kinase domain containing protein [Tetrahymena thermophila] | 6.00E-05 |
| scaffold_422:5384..6421 | 611 | hypothetical protein TTHERM_00093930 [Tetrahymena thermophila] | 7.00E-27 |
| scaffold_422:3862..5040 | 691 | hypothetical protein TTHERM_00093930 [Tetrahymena thermophila] | 2.00E-13 |
| scaffold_422:16189..17804 | 862 | hypothetical protein TTHERM_01230160 [Tetrahymena thermophila] | 6.00E-12 |
| scaffold_420:6099..7128 | 974 | hypothetical protein TTHERM_01217180 [Tetrahymena thermophila] | 8.00E-06 |
| scaffold_413:8450..9017 | 568 | hypothetical protein TTHERM_00746910 [Tetrahymena thermophila] | 3.00E-16 |
| scaffold_413:7353..7895 | 543 | hypothetical protein TTHERM_01595620 [Tetrahymena thermophila] | 2.00E-25 |
| scaffold_411:11938..17581 | 2038 | hypothetical protein TTHERM_01303860 [Tetrahymena thermophila] | 2.00E-44 |
| scaffold_402:431..963 | 533 | hypothetical protein TTHERM_00990520 [Tetrahymena thermophila] | 3.00E-28 |
| scaffold_400:18795..19332 | 538 | hypothetical protein TTHERM_01595620 [Tetrahymena thermophila] | 7.00E-13 |
| scaffold_400:17361..18196 | 537 | conserved hypothetical protein [Tetrahymena thermophila] | 6.00E-18 |
| scaffold_392:19166..20431 | 1266 | hypothetical protein TTHERM_00990520 [Tetrahymena thermophila] | 2.00E-24 |
| scaffold_392:11..560 | 550 | Protein kinase domain containing protein [Tetrahymena thermophila] | 6.00E-31 |
| scaffold_391:14416..15242 | 827 | hypothetical protein TTHERM_02579220 [Tetrahymena thermophila] | 2.00E-07 |
| scaffold_391:13344..13660 | 317 | conserved hypothetical protein [Tetrahymena thermophila] | 3.00E-09 |
| scaffold_386:3822..4164 | 343 | hypothetical protein ZyciCp036 [Zygnema circumcarinatum] | 2.00E-06 |
| scaffold_384:16395..17797 | 434 | Leucine Rich Repeat family protein [Tetrahymena thermophila] | 9.00E-06 |
| scaffold_3836:892827..894614 | 999 | conserved hypothetical protein [Tetrahymena thermophila] | 1.00E-19 |
| scaffold_3836:525066..525946 | 881 | hypothetical protein TTHERM_00242400 [Tetrahymena thermophila] | 3.00E-16 |
| scaffold_3836:523805..524330 | 526 | TPR Domain containing protein [Tetrahymena thermophila] | 3.00E-25 |
| scaffold_3836:519924..520712 | 789 | hypothetical protein TTHERM_00242400 [Tetrahymena thermophila] | 6.00E-19 |
| scaffold_3836:505062..516253 | 10967 | hypothetical protein TTHERM_01466260 [Tetrahymena thermophila] | 0 |
| scaffold_3836:505027..506118 | 1092 | Leishmanolysin family protein [Tetrahymena thermophila] | 6.00E-62 |
| scaffold_3836:493237..496081 | 2000 | hypothetical protein [Paramecium tetraurelia strain d4-2] | 1.00E-13 |
| scaffold_3836:366601..367436 | 836 | TPR Domain containing protein [Tetrahymena thermophila] | 1.00E-17 |
| scaffold_3836:364672..366133 | 1402 | hypothetical protein TTHERM_00090360 [Tetrahymena thermophila] | 3.00E-28 |
| scaffold_3836:364672..366133 | 1356 | hypothetical protein TTHERM_00090360 [Tetrahymena thermophila] | 3.00E-28 |
| scaffold_3836:336394..337056 | 663 | TPR Domain containing protein [Tetrahymena thermophila] | 5.00E-06 |
| scaffold_3836:335419..337056 | 1518 | TPR Domain containing protein [Tetrahymena thermophila] | 1.00E-12 |
| scaffold_3836:333572..336256 | 1136 | hypothetical protein TTHERM_00090360 [Tetrahymena thermophila] | 1.00E-27 |
| scaffold_3836:1771671..1772099 | 429 | hypothetical protein TTHERM_00625930 [Tetrahymena thermophila] | 2.00E-13 |
| scaffold_3836:1768503..1768888 | 386 | hypothetical protein TTHERM_00990520 [Tetrahymena thermophila] | 3.00E-26 |
| scaffold_3836:1761906..1762349 | 444 | hypothetical protein TTHERM_00990520 [Tetrahymena thermophila] | 2.00E-19 |
| scaffold_3836:146374..152688 | 3714 | Leucine Rich Repeat family protein [Tetrahymena thermophila] | 1.00E-145 |
| scaffold_3836:1220046..1225085 | 4950 | hypothetical protein TTHERM_00354580 [Tetrahymena thermophila] | 0 |
| scaffold_3836:1212513..1214983 | 2163 | hypothetical protein TTHERM_00470800 [Tetrahymena thermophila] | 2.00E-31 |
| scaffold_3836:1176268..1177270 | 923 | hypothetical protein TTHERM_00083280 [Tetrahymena thermophila] | 3.00E-58 |
| scaffold_3835:85641..85941 | 301 | hypothetical protein TTHERM_00592930 [Tetrahymena thermophila] | 8.00E-04 |
| scaffold_3835:75782..76902 | 1121 | hypothetical protein TTHERM_00443080 [Tetrahymena thermophila] | 1.00E-08 |
| scaffold_3835:468995..469314 | 320 | hypothetical protein TTHERM_01527450 [Tetrahymena thermophila] | 4.00E-40 |
| scaffold_3835:459231..459915 | 428 | hypothetical protein TTHERM_00718050 [Tetrahymena thermophila] | 7.00E-16 |
| scaffold_3835:437356..440995 | 2979 | hypothetical protein TTHERM_00767530 [Tetrahymena thermophila] | 0 |
| scaffold_3835:434616..437141 | 2193 | hypothetical protein TTHERM_00790970 [Tetrahymena thermophila] | 0 |
| scaffold_3835:417172..417495 | 324 | hypothetical protein TTHERM_00598660 [Tetrahymena thermophila] | 3.00E-08 |
| scaffold_3835:411714..413221 | 1359 | hypothetical protein TTHERM_01273230 [Tetrahymena thermophila] | 1.00E-165 |
| scaffold_3835:384068..384592 | 525 | hypothetical protein TTHERM_00598660 [Tetrahymena thermophila] | 1.00E-21 |
| scaffold_3835:382853..383802 | 950 | hypothetical protein TTHERM_00598660 [Tetrahymena thermophila] | 1.00E-114 |
| scaffold_3835:381790..382582 | 687 | hypothetical protein TTHERM_00598660 [Tetrahymena thermophila] | 3.00E-82 |
| scaffold_3835:380006..381029 | 824 | hypothetical protein TTHERM_00598660 [Tetrahymena thermophila] | 3.00E-72 |
| scaffold_3835:298208..301518 | 2817 | hypothetical protein TTHERM_00363140 [Tetrahymena thermophila] | 1.00E-156 |
| scaffold_3835:254303..255386 | 1084 | hypothetical protein TTHERM_00595440 [Tetrahymena thermophila] | 1.00E-111 |
| scaffold_3835:132891..133858 | 641 | hypothetical protein [Paramecium tetraurelia strain d4-2] | 2.00E-53 |
| scaffold_3833:808052..810710 | 1783 | hypothetical protein TTHERM_00802510 [Tetrahymena thermophila] | 4.00E-06 |
| scaffold_3833:759802..760337 | 536 | hypothetical protein TTHERM_00886900 [Tetrahymena thermophila] | 5.00E-61 |
| scaffold_3833:756102..759626 | 3238 | hypothetical protein TTHERM_00886900 [Tetrahymena thermophila] | 0 |
| scaffold_3833:750744..755795 | 4683 | hypothetical protein TTHERM_00885880 [Tetrahymena thermophila] | 0 |
| scaffold_3833:749322..749642 | 321 | Calpain family cysteine protease containing protein [Tetrahymena thermophila] | 6.00E-27 |
| scaffold_3833:602597..603764 | 1168 | hypothetical protein TTHERM_00365470 [Tetrahymena thermophila] | 2.00E-14 |
| scaffold_3833:424983..427146 | 2164 | ABC transporter family protein [Tetrahymena thermophila] | 1.00E-109 |
| scaffold_3833:422188..424366 | 2179 | ABC transporter family protein [Tetrahymena thermophila] | 1.00E-117 |
| scaffold_3833:331385..333485 | 2101 | hypothetical protein TTHERM_00417840 [Tetrahymena thermophila] | 0 |
| scaffold_3833:324444..328041 | 3598 | hypothetical protein TTHERM_00417840 [Tetrahymena thermophila] | 0 |
| scaffold_3833:319890..321246 | 1357 | hypothetical protein TTHERM_00417840 [Tetrahymena thermophila] | 1.00E-173 |
| scaffold_3833:311958..314734 | 2777 | hypothetical protein TTHERM_00417840 [Tetrahymena thermophila] | 0 |
| scaffold_3833:306219..306729 | 511 | hypothetical protein TTHERM_00417840 [Tetrahymena thermophila] | 7.00E-57 |
| scaffold_3833:231090..232028 | 939 | hypothetical protein TTHERM_00417960 [Tetrahymena thermophila] | 1.00E-31 |
| scaffold_3833:230482..230971 | 490 | hypothetical protein TTHERM_00417990 [Tetrahymena thermophila] | 2.00E-38 |
| scaffold_3833:229120..230372 | 1253 | hypothetical protein TTHERM_00418000 [Tetrahymena thermophila] | 3.00E-88 |
| scaffold_3833:227615..228181 | 567 | hypothetical protein TTHERM_00418000 [Tetrahymena thermophila] | 2.00E-40 |
| scaffold_3833:225606..226033 | 428 | hypothetical protein TTHERM_00418000 [Tetrahymena thermophila] | 3.00E-39 |
| scaffold_3831:854148..855155 | 441 | hypothetical protein TTHERM_01256640 [Tetrahymena thermophila] | 1.00E-14 |
| scaffold_3831:851899..852292 | 394 | hypothetical protein TTHERM_01458110 [Tetrahymena thermophila] | 9.00E-04 |
| scaffold_3831:850248..851460 | 463 | hypothetical protein TTHERM_01256620 [Tetrahymena thermophila] | 3.00E-13 |
| scaffold_3831:848356..849300 | 560 | Leucine Rich Repeat family protein [Tetrahymena thermophila] | 8.00E-10 |
| scaffold_3831:542155..542837 | 683 | hypothetical protein TTHERM_01090200 [Tetrahymena thermophila] | 2.00E-70 |
| scaffold_3831:504711..506239 | 1464 | hypothetical protein TTHERM_01089040 [Tetrahymena thermophila] | 1.00E-114 |
| scaffold_3831:503851..504346 | 496 | hypothetical protein TTHERM_01090090 [Tetrahymena thermophila] | 2.00E-71 |
| scaffold_3831:502478..503720 | 1125 | hypothetical protein TTHERM_01090090 [Tetrahymena thermophila] | 3.00E-99 |
| scaffold_3831:501330..502176 | 791 | hypothetical protein TTHERM_01090090 [Tetrahymena thermophila] | 4.00E-63 |
| scaffold_3831:499209..501202 | 1527 | hypothetical protein TTHERM_01090090 [Tetrahymena thermophila] | 1.00E-127 |
| scaffold_3831:487390..488330 | 876 | hypothetical protein TTHERM_01090090 [Tetrahymena thermophila] | 2.00E-86 |
| scaffold_3831:481594..482314 | 721 | Neurohypophysial hormone, N-terminal Domain containing protein [Tetrahymena thermophila] | 2.00E-77 |
| scaffold_3831:467152..467563 | 412 | conserved hypothetical protein [Tetrahymena thermophila] | 2.00E-28 |
| scaffold_3831:458708..459061 | 354 | Neurohypophysial hormone, N-terminal Domain containing protein [Tetrahymena thermophila] | 8.00E-42 |
| scaffold_3831:397222..398582 | 1361 | TPR Domain containing protein [Tetrahymena thermophila] | 1.00E-51 |
| scaffold_3831:374421..376037 | 1617 | TPR Domain containing protein [Tetrahymena thermophila] | 4.00E-63 |
| scaffold_3831:365636..367034 | 1050 | hypothetical protein TTHERM_01423400 [Tetrahymena thermophila] | 3.00E-14 |
| scaffold_3831:355836..356445 | 383 | hypothetical protein TTHERM_01049170 [Tetrahymena thermophila] | 4.00E-07 |
| scaffold_3831:344756..345601 | 787 | hypothetical protein TTHERM_01423400 [Tetrahymena thermophila] | 2.00E-21 |
| scaffold_3831:332029..332740 | 656 | hypothetical protein TTHERM_00629760 [Tetrahymena thermophila] | 4.00E-24 |
| scaffold_3831:232865..233937 | 760 | hypothetical protein TTHERM_00630530 [Tetrahymena thermophila] | 6.00E-05 |
| scaffold_3831:122290..122843 | 554 | hypothetical protein TTHERM_00580369 [Tetrahymena thermophila] | 1.00E-16 |
| scaffold_3831:1172222..1173466 | 1066 | hypothetical protein TTHERM_01527490 [Tetrahymena thermophila] | 1.00E-111 |
| scaffold_3831:1157563..1158368 | 754 | hypothetical protein TTHERM_00767530 [Tetrahymena thermophila] | 1.00E-113 |
| scaffold_3831:1154525..1156698 | 1916 | hypothetical protein TTHERM_00790970 [Tetrahymena thermophila] | 0 |
| scaffold_3831:1151739..1154413 | 2169 | hypothetical protein TTHERM_00790970 [Tetrahymena thermophila] | 0 |
| scaffold_3831:1151215..1151615 | 349 | hypothetical protein TTHERM_00561350 [Tetrahymena thermophila] | 1.00E-05 |
| scaffold_3831:1144254..1144670 | 417 | hypothetical protein TTHERM_01273230 [Tetrahymena thermophila] | 1.00E-54 |
| scaffold_3831:1143334..1144036 | 651 | hypothetical protein TTHERM_01527490 [Tetrahymena thermophila] | 1.00E-103 |
| scaffold_3831:1140349..1141553 | 1205 | hypothetical protein TTHERM_00790970 [Tetrahymena thermophila] | 1.00E-131 |
| scaffold_3831:1138925..1139887 | 882 | hypothetical protein TTHERM_00598670 [Tetrahymena thermophila] | 8.00E-87 |
| scaffold_3830:400474..402050 | 1519 | hypothetical protein TTHERM_00532880 [Tetrahymena thermophila] | 1.00E-73 |
| scaffold_3829:973383..973777 | 395 | hypothetical protein TTHERM_01589520 [Tetrahymena thermophila] | 3.00E-18 |
| scaffold_3829:968547..973159 | 4613 | hypothetical protein TTHERM_01589520 [Tetrahymena thermophila] | 0 |
| scaffold_3829:967034..967401 | 368 | hypothetical protein TTHERM_01589520 [Tetrahymena thermophila] | 6.00E-39 |
| scaffold_3829:948510..949248 | 739 | hypothetical protein TTHERM_00155280 [Tetrahymena thermophila] | 1.00E-94 |
| scaffold_3829:947168..948240 | 1073 | hypothetical protein TTHERM_00155280 [Tetrahymena thermophila] | 3.00E-88 |
| scaffold_3829:946132..946491 | 360 | hypothetical protein TTHERM_00155280 [Tetrahymena thermophila] | 1.00E-15 |
| scaffold_3829:571999..572593 | 371 | SLEI family protein [Tetrahymena thermophila] | 1.00E-05 |
| scaffold_3829:481612..482269 | 658 | hypothetical protein TTHERM_00160540 [Tetrahymena thermophila] | 4.00E-39 |
| scaffold_3829:470257..471258 | 1002 | hypothetical protein TTHERM_00160540 [Tetrahymena thermophila] | 2.00E-24 |
| scaffold_3829:39539..44250 | 3211 | LOC100145576 protein [Xenopus (Silurana) tropicalis] | 1.00E-31 |
| scaffold_3829:1034918..1039132 | 3774 | zinc finger domain, LSD1 subclass family protein [Tetrahymena thermophila] | 0 |
| scaffold_3829:1032296..1033263 | 968 | zinc finger domain, LSD1 subclass family protein [Tetrahymena thermophila] | 1.00E-151 |
| scaffold_3829:1010484..1016167 | 5480 | zinc finger domain, LSD1 subclass family protein [Tetrahymena thermophila] | 0 |
| scaffold_3829:1009546..1009864 | 319 | zinc finger domain, LSD1 subclass family protein [Tetrahymena thermophila] | 3.00E-54 |
| scaffold_3829:1007033..1008352 | 1320 | zinc finger domain, LSD1 subclass family protein [Tetrahymena thermophila] | 0 |
| scaffold_3829:1005886..1006841 | 956 | zinc finger domain, LSD1 subclass family protein [Tetrahymena thermophila] | 1.00E-120 |
| scaffold_3828:791414..796165 | 3596 | hypothetical protein [Paramecium tetraurelia strain d4-2] | 5.00E-12 |
| scaffold_3828:7388..8148 | 475 | hypothetical protein TTHERM_00630640 [Tetrahymena thermophila] | 4.00E-27 |
| scaffold_3828:54596..55716 | 1059 | hypothetical protein TTHERM_00329980 [Tetrahymena thermophila] | 3.00E-15 |
| scaffold_3828:53184..53786 | 603 | hypothetical protein TTHERM_00074250 [Tetrahymena thermophila] | 8.00E-42 |
| scaffold_3828:49727..50424 | 698 | hypothetical protein TTHERM_00346680 [Tetrahymena thermophila] | 1.00E-22 |
| scaffold_3828:48621..49382 | 762 | hypothetical protein TTHERM_00074250 [Tetrahymena thermophila] | 3.00E-14 |
| scaffold_3828:46667..47388 | 722 | hypothetical protein TTHERM_00329980 [Tetrahymena thermophila] | 4.00E-05 |
| scaffold_3828:336..3099 | 1232 | hypothetical protein TTHERM_01434640 [Tetrahymena thermophila] | 1.00E-68 |
| scaffold_3828:1216002..1216853 | 608 | hypothetical protein TTHERM_00990520 [Tetrahymena thermophila] | 6.00E-16 |
| scaffold_3828:114164..114473 | 310 | hypothetical protein TTHERM_00092900 [Tetrahymena thermophila] | 3.00E-13 |
| scaffold_3828:113478..113849 | 372 | hypothetical protein TTHERM_00625930 [Tetrahymena thermophila] | 4.00E-23 |
| scaffold_3827:713449..714006 | 558 | hypothetical protein TTHERM_00598670 [Tetrahymena thermophila] | 4.00E-78 |
| scaffold_3827:709353..710034 | 682 | hypothetical protein TTHERM_01273230 [Tetrahymena thermophila] | 1.00E-79 |
| scaffold_3827:693030..694751 | 1595 | hypothetical protein TTHERM_01273230 [Tetrahymena thermophila] | 1.00E-161 |
| scaffold_3827:414001..414813 | 813 | hypothetical protein TTHERM_00717410 [Tetrahymena thermophila] | 1.00E-134 |
| scaffold_3827:410892..412005 | 1114 | hypothetical protein TTHERM_00717430 [Tetrahymena thermophila] | 1.00E-111 |
| scaffold_3827:40071..40927 | 857 | hypothetical protein TTHERM_00346580 [Tetrahymena thermophila] | 1.00E-04 |
| scaffold_3826:36947..38099 | 810 | hypothetical protein TTHERM_01034450 [Tetrahymena thermophila] | 1.00E-04 |
| scaffold_3826:1539..14326 | 10928 | hypothetical protein [Paramecium tetraurelia strain d4-2] | 0 |
| scaffold_3825:999527..1001065 | 1277 | ABC transporter family protein [Tetrahymena thermophila] | 1.00E-146 |
| scaffold_3825:846664..850133 | 3100 | hypothetical protein TTHERM_00590410 [Tetrahymena thermophila] | 7.00E-23 |
| scaffold_3825:767..3319 | 2254 | hypothetical protein [Paramecium tetraurelia strain d4-2] | 5.00E-07 |
| scaffold_3825:361582..367759 | 4878 | hypothetical protein TTHERM_00683320 [Tetrahymena thermophila] | 0 |
| scaffold_3825:350543..351061 | 519 | hypothetical protein TTHERM_00652500 [Tetrahymena thermophila] | 4.00E-08 |
| scaffold_3825:346402..346996 | 595 | hypothetical protein TTHERM_00683320 [Tetrahymena thermophila] | 5.00E-15 |
| scaffold_3825:336354..337508 | 1099 | hypothetical protein TTHERM_00652500 [Tetrahymena thermophila] | 4.00E-78 |
| scaffold_3825:335321..335978 | 557 | hypothetical protein TTHERM_00652500 [Tetrahymena thermophila] | 4.00E-33 |
| scaffold_3825:333593..335220 | 1463 | hypothetical protein TTHERM_00652510 [Tetrahymena thermophila] | 4.00E-36 |
| scaffold_3825:331171..333376 | 1974 | hypothetical protein TTHERM_00683320 [Tetrahymena thermophila] | 4.00E-32 |
| scaffold_3825:326572..327407 | 446 | hypothetical protein TTHERM_00037700 [Tetrahymena thermophila] | 6.00E-28 |
| scaffold_3825:325322..326219 | 843 | hypothetical protein TTHERM_00011910 [Tetrahymena thermophila] | 7.00E-19 |
| scaffold_3825:324822..325234 | 413 | hypothetical protein TTHERM_00683320 [Tetrahymena thermophila] | 6.00E-16 |
| scaffold_3825:287980..288552 | 573 | hypothetical protein TTHERM_00762990 [Tetrahymena thermophila] | 3.00E-50 |
| scaffold_3825:2122139..2123636 | 683 | hypothetical protein TTHERM_01384020 [Tetrahymena thermophila] | 2.00E-20 |
| scaffold_3825:2120218..2121506 | 495 | conserved hypothetical protein [Tetrahymena thermophila] | 6.00E-15 |
| scaffold_3825:2116231..2117148 | 429 | hypothetical protein TTHERM_01358420 [Tetrahymena thermophila] | 5.00E-15 |
| scaffold_3825:1988256..1990396 | 1479 | PREDICTED: hypothetical protein [Taeniopygia guttata] | 4.00E-12 |
| scaffold_3825:1792612..1795376 | 1255 | hypothetical protein TTHERM_01256640 [Tetrahymena thermophila] | 3.00E-21 |
| scaffold_3825:1367749..1368566 | 818 | hypothetical protein TTHERM_00035720 [Tetrahymena thermophila] | 9.00E-63 |
| scaffold_3825:1137455..1138132 | 447 | YEE9 protein [Oncorhynchus mykiss] | 1.00E-05 |
| scaffold_3825:1006225..1006910 | 625 | ABC transporter family protein [Tetrahymena thermophila] | 3.00E-60 |
| scaffold_3824:609570..610207 | 465 | hypothetical protein [Paramecium tetraurelia strain d4-2] | 6.00E-15 |
| scaffold_3824:378247..381804 | 1045 | hypothetical protein TTHERM_00046620 [Tetrahymena thermophila] | 1.00E-48 |
| scaffold_3824:1573599..1574711 | 1113 | hypothetical protein TTHERM_00194530 [Tetrahymena thermophila] | 6.00E-24 |
| scaffold_3823:672721..674794 | 2074 | TPR Domain containing protein [Tetrahymena thermophila] | 6.00E-14 |
| scaffold_3823:596515..597224 | 710 | hypothetical protein TTHERM_01159890 [Tetrahymena thermophila] | 1.00E-116 |
| scaffold_3823:586591..586942 | 352 | hypothetical protein TTHERM_01215030 [Tetrahymena thermophila] | 4.00E-21 |
| scaffold_3823:584934..585677 | 744 | hypothetical protein TTHERM_01215030 [Tetrahymena thermophila] | 7.00E-90 |
| scaffold_3823:570288..571556 | 381 | hypothetical protein TTHERM_00860490 [Tetrahymena thermophila] | 9.00E-30 |
| scaffold_3823:387105..387532 | 428 | dynein light chain 8-like A [Tetrahymena thermophila] | 2.00E-46 |
| scaffold_3823:138843..139676 | 611 | hypothetical protein TTHERM_00462810 [Tetrahymena thermophila] | 6.00E-26 |
| scaffold_3823:137647..138775 | 744 | hypothetical protein TTHERM_00462820 [Tetrahymena thermophila] | 9.00E-24 |
| scaffold_3823:135565..137078 | 1449 | hypothetical protein TTHERM_00462810 [Tetrahymena thermophila] | 8.00E-31 |
| scaffold_3823:133348..134720 | 1311 | hypothetical protein TTHERM_00462810 [Tetrahymena thermophila] | 2.00E-27 |
| scaffold_3822:452938..454067 | 1130 | cyclase family protein [Lactobacillus brevis subsp. gravesensis ATCC 27305] | 3.00E-06 |
| scaffold_3822:424133..425361 | 461 | hypothetical protein TTHERM_00862730 [Tetrahymena thermophila] | 9.00E-19 |
| scaffold_3822:385789..386410 | 622 | hypothetical protein TTHERM_01484660 [Tetrahymena thermophila] | 1.00E-41 |
| scaffold_3822:374181..375937 | 625 | hypothetical protein TTHERM_00170139 [Tetrahymena thermophila] | 1.00E-33 |
| scaffold_3822:360613..361912 | 998 | hypothetical protein [Paramecium tetraurelia strain d4-2] | 2.00E-24 |
| scaffold_3822:319089..319644 | 556 | hypothetical protein TTHERM_00990520 [Tetrahymena thermophila] | 1.00E-17 |
| scaffold_3822:216549..217714 | 356 | hypothetical protein TTHERM_01674210 [Tetrahymena thermophila] | 3.00E-21 |
| scaffold_3822:214632..216249 | 597 | SLEI family protein [Tetrahymena thermophila] | 2.00E-26 |
| scaffold_3821:221479..221921 | 443 | hypothetical protein TTHERM_00759070 [Tetrahymena thermophila] | 4.00E-58 |
| scaffold_3820:26244..29325 | 2923 | hypothetical protein TTHERM_00334370 [Tetrahymena thermophila] | 2.00E-21 |
| scaffold_3819:92533..97120 | 3897 | GG25361, isoform B [Drosophila erecta] | 2.00E-06 |
| scaffold_3819:92533..95440 | 2908 | GG25361, isoform B [Drosophila erecta] | 2.00E-06 |
| scaffold_3819:139334..140086 | 753 | hypothetical protein TTHERM_00990520 [Tetrahymena thermophila] | 5.00E-22 |
| scaffold_3819:138778..139139 | 362 | hypothetical protein TTHERM_01017240 [Tetrahymena thermophila] | 1.00E-15 |
| scaffold_3819:126850..127158 | 309 | hypothetical protein TTHERM_01035620 [Tetrahymena thermophila] | 8.00E-13 |
| scaffold_3817:88010..89766 | 1757 | cyclic nucleotide-binding domain containing protein [Tetrahymena thermophila] | 2.00E-05 |
| scaffold_3816:64404..66207 | 1150 | hypothetical protein TTHERM_01097910 [Tetrahymena thermophila] | 4.00E-86 |
| scaffold_3816:60566..61354 | 789 | hypothetical protein TTHERM_01097910 [Tetrahymena thermophila] | 4.00E-75 |
| scaffold_3816:105..1642 | 1357 | hypothetical protein [Paramecium tetraurelia strain d4-2] | 8.00E-65 |
| scaffold_3815:32920..38740 | 2976 | zinc finger CCCH type domain containing protein [Tetrahymena thermophila] | 1.00E-77 |
| scaffold_3814:58022..59581 | 1145 | hypothetical protein TTHERM_00622650 [Tetrahymena thermophila] | 2.00E-11 |
| scaffold_3814:56655..57826 | 1172 | hypothetical protein TTHERM_00622650 [Tetrahymena thermophila] | 1.00E-144 |
| scaffold_3814:2334..2792 | 459 | hypothetical protein TTHERM_01264990 [Tetrahymena thermophila] | 1.00E-22 |
| scaffold_3813:797030..797385 | 356 | hypothetical protein TTHERM_01673190 [Tetrahymena thermophila] | 2.00E-25 |
| scaffold_3813:796402..796888 | 487 | hypothetical protein TTHERM_00625930 [Tetrahymena thermophila] | 1.00E-16 |
| scaffold_3813:795634..796205 | 572 | hypothetical protein TTHERM_00625930 [Tetrahymena thermophila] | 6.00E-31 |
| scaffold_3813:795047..795513 | 467 | hypothetical protein TTHERM_00625930 [Tetrahymena thermophila] | 2.00E-38 |
| scaffold_3813:605495..606795 | 1301 | hypothetical protein TTHERM_00391240 [Tetrahymena thermophila] | 1.00E-146 |
| scaffold_3813:604935..605319 | 385 | hypothetical protein TTHERM_00391240 [Tetrahymena thermophila] | 4.00E-36 |
| scaffold_3813:525789..526461 | 673 | OsmC-like protein [Tetrahymena thermophila] | 2.00E-37 |
| scaffold_3813:512227..512675 | 449 | hypothetical protein TTHERM_00391470 [Tetrahymena thermophila] | 8.00E-18 |
| scaffold_3813:404442..405308 | 867 | hypothetical protein TTHERM_00392770 [Tetrahymena thermophila] | 1.00E-45 |
| scaffold_3813:229052..229683 | 632 | cation channel family protein [Tetrahymena thermophila] | 0.001 |
| scaffold_3812:815290..816136 | 711 | unnamed protein product [Vitis vinifera] | 7.00E-04 |
| scaffold_3812:711611..712970 | 1360 | hypothetical protein TTHERM_00112900 [Tetrahymena thermophila] | 3.00E-52 |
| scaffold_3812:703999..706021 | 1864 | hypothetical protein TTHERM_00112900 [Tetrahymena thermophila] | 3.00E-71 |
| scaffold_3812:467901..470351 | 2225 | hypothetical protein TTHERM_00109240 [Tetrahymena thermophila] | 0 |
| scaffold_3812:465178..467426 | 2249 | Protein kinase domain containing protein [Tetrahymena thermophila] | 8.00E-28 |
| scaffold_3812:460009..462263 | 2255 | hypothetical protein TTHERM_00109240 [Tetrahymena thermophila] | 0 |
| scaffold_3812:455581..456693 | 1113 | hypothetical protein TTHERM_00109240 [Tetrahymena thermophila] | 4.00E-45 |
| scaffold_3812:453299..455074 | 1776 | Protein kinase domain containing protein [Tetrahymena thermophila] | 2.00E-15 |
| scaffold_3812:423162..423870 | 709 | hypothetical protein TTHERM_00573210 [Tetrahymena thermophila] | 1.00E-71 |
| scaffold_3812:421833..422412 | 580 | hypothetical protein TTHERM_00573210 [Tetrahymena thermophila] | 2.00E-70 |
| scaffold_3812:407964..412036 | 4073 | hypothetical protein TTHERM_00109250 [Tetrahymena thermophila] | 0 |
| scaffold_3812:406367..407791 | 1425 | hypothetical protein TTHERM_00109250 [Tetrahymena thermophila] | 1.00E-159 |
| scaffold_3812:363543..363897 | 355 | hypothetical protein TTHERM_00574320 [Tetrahymena thermophila] | 2.00E-31 |
| scaffold_3812:260140..263514 | 3174 | hypothetical protein TTHERM_01902130 [Tetrahymena thermophila] | 1.00E-100 |
| scaffold_3812:1079082..1079700 | 619 | Leucine Rich Repeat family protein [Tetrahymena thermophila] | 4.00E-98 |
| scaffold_3812:1074318..1074848 | 418 | Leucine Rich Repeat family protein [Tetrahymena thermophila] | 8.00E-60 |
| scaffold_3812:1072535..1073089 | 555 | Leucine Rich Repeat family protein [Tetrahymena thermophila] | 1.00E-42 |
| scaffold_3811:400405..403126 | 2363 | hypothetical protein [Paramecium tetraurelia strain d4-2] | 1.00E-06 |
| scaffold_3811:17665..19357 | 1639 | Protein kinase domain containing protein [Tetrahymena thermophila] | 7.00E-12 |
| scaffold_3811:122009..123880 | 1587 | hypothetical protein TTHERM_00448820 [Tetrahymena thermophila] | 1.00E-143 |
| scaffold_3811:118587..120405 | 1601 | hypothetical protein TTHERM_00448820 [Tetrahymena thermophila] | 1.00E-135 |
| scaffold_3810:956352..958131 | 1780 | Protein kinase domain containing protein [Tetrahymena thermophila] | 4.00E-35 |
| scaffold_3810:944460..945663 | 1204 | histone H3 [Tetrahymena thermophila] | 4.00E-65 |
| scaffold_3810:416804..417383 | 343 | hypothetical protein TTHERM_01232230 [Tetrahymena thermophila] | 1.00E-04 |
| scaffold_3809:10549..11858 | 549 | hypothetical protein TTHERM_00166070 [Tetrahymena thermophila] | 2.00E-09 |
| scaffold_3808:44138..49448 | 5257 | Protein kinase domain containing protein [Tetrahymena thermophila] | 1.00E-10 |
| scaffold_3808:22662..23404 | 743 | hypothetical protein TTHERM_00801270 [Tetrahymena thermophila] | 5.00E-29 |
| scaffold_3808:22069..22540 | 472 | hypothetical protein TTHERM_00538690 [Tetrahymena thermophila] | 5.00E-07 |
| scaffold_3808:21253..21956 | 646 | hypothetical protein TTHERM_00538640 [Tetrahymena thermophila] | 1.00E-33 |
| scaffold_3808:190024..192462 | 1864 | hypothetical protein [Paramecium tetraurelia strain d4-2] | 3.00E-16 |
| scaffold_3808:114212..120939 | 1398 | hypothetical protein TTHERM_01256640 [Tetrahymena thermophila] | 2.00E-35 |
| scaffold_3806:511..1163 | 653 | hypothetical protein [Paramecium tetraurelia strain d4-2] | 8.00E-12 |
| scaffold_3801:82325..83362 | 924 | CDP-alcohol phosphatidyltransferase family protein [Synechococcus sp. JA-2-3B'a(2-13)] | 6.00E-30 |
| scaffold_38:622264..622771 | 508 | hypothetical protein TTHERM_01673190 [Tetrahymena thermophila] | 5.00E-26 |
| scaffold_38:621684..622159 | 476 | hypothetical protein TTHERM_01673190 [Tetrahymena thermophila] | 3.00E-23 |
| scaffold_38:562967..563697 | 369 | Protein kinase domain containing protein [Tetrahymena thermophila] | 2.00E-09 |
| scaffold_38:548737..549233 | 497 | hypothetical protein TTHERM_00365470 [Tetrahymena thermophila] | 3.00E-55 |
| scaffold_3799:113157..113922 | 390 | hypothetical protein TTHERM_00313440 [Tetrahymena thermophila] | 2.00E-05 |
| scaffold_3798:37591..38711 | 684 | hypothetical protein TTHERM_00253469 [Tetrahymena thermophila] | 9.00E-49 |
| scaffold_3798:35896..36853 | 958 | hypothetical protein TTHERM_00253468 [Tetrahymena thermophila] | 7.00E-34 |
| scaffold_3798:113882..115412 | 1313 | hypothetical protein [Paramecium tetraurelia strain d4-2] | 1.00E-80 |
| scaffold_3797:456..1415 | 960 | hypothetical protein [Paramecium tetraurelia strain d4-2] | 6.00E-17 |
| scaffold_3795:40142..42052 | 1533 | hypothetical protein [Paramecium tetraurelia strain d4-2] | 9.00E-06 |
| scaffold_377:10635..12527 | 880 | hypothetical protein TTHERM_00255719 [Tetrahymena thermophila] | 3.00E-26 |
| scaffold_3761:147762..151103 | 3342 | Response regulator receiver domain containing protein [Tetrahymena thermophila] | 3.00E-76 |
| scaffold_376:3670..4341 | 366 | hypothetical protein TTHERM_00902820 [Tetrahymena thermophila] | 3.00E-09 |
| scaffold_376:2662..3541 | 524 | conserved hypothetical protein [Tetrahymena thermophila] | 1.00E-13 |
| scaffold_376:1037..1880 | 328 | hypothetical protein TTHERM_00170139 [Tetrahymena thermophila] | 2.00E-04 |
| scaffold_3759:232111..232677 | 468 | hypothetical protein [Paramecium tetraurelia strain d4-2] | 6.00E-21 |
| scaffold_3759:127208..127629 | 422 | hypothetical protein TTHERM_01484660 [Tetrahymena thermophila] | 4.00E-31 |
| scaffold_3757:662..3729 | 3068 | hypothetical protein RUMGNA_00661 [Ruminococcus gnavus ATCC 29149] | 3.00E-37 |
| scaffold_3755:56918..67480 | 8927 | cation channel family protein [Tetrahymena thermophila] | 0 |
| scaffold_3753:257693..259166 | 1474 | hypothetical protein TTHERM_01085730 [Tetrahymena thermophila] | 2.00E-67 |
| scaffold_3753:175091..177402 | 2257 | ABC transporter family protein [Tetrahymena thermophila] | 0 |
| scaffold_3753:173886..174887 | 939 | daunorubicin resistance ABC transporter ATP-binding subunit family protein [Tetrahymena thermophila] | 1.00E-145 |
| scaffold_3753:172345..173218 | 874 | ABC transporter family protein [Tetrahymena thermophila] | 1.00E-109 |
| scaffold_3752:184401..185091 | 691 | peptide deformylase [Streptobacillus moniliformis DSM 12112] | 2.00E-16 |
| scaffold_3751:125096..125758 | 663 | hypothetical protein TTHERM_01401760 [Tetrahymena thermophila] | 4.00E-45 |
| scaffold_3750:77272..78084 | 539 | hypothetical protein TTHERM_00335870 [Tetrahymena thermophila] | 2.00E-06 |
| scaffold_3750:212454..213254 | 801 | GINS complex subunit Psf3 [Ajellomyces dermatitidis SLH14081] | 4.00E-13 |
| scaffold_3750:138801..146883 | 7686 | hypothetical protein TTHERM_00850520 [Tetrahymena thermophila] | 0 |
| scaffold_3750:137038..137494 | 457 | hypothetical protein TTHERM_00850520 [Tetrahymena thermophila] | 3.00E-67 |
| scaffold_3750:135085..136141 | 1057 | hypothetical protein TTHERM_00850520 [Tetrahymena thermophila] | 2.00E-73 |
| scaffold_3749:418..3723 | 2929 | hypothetical protein SCHCODRAFT_69938 [Schizophyllum commune H4-8] | 1.00E-19 |
| scaffold_3749:31108..31914 | 357 | hypothetical protein TTHERM_01306860 [Tetrahymena thermophila] | 5.00E-10 |
| scaffold_3745:62121..62893 | 501 | hypothetical protein TTHERM_00278640 [Tetrahymena thermophila] | 2.00E-04 |
| scaffold_3745:59881..61955 | 669 | hypothetical protein TTHERM_00797810 [Tetrahymena thermophila] | 2.00E-31 |
| scaffold_3745:56184..59015 | 859 | hypothetical protein TTHERM_00797810 [Tetrahymena thermophila] | 4.00E-33 |
| scaffold_3745:181541..182537 | 997 | hypothetical protein [Paramecium tetraurelia strain d4-2] | 2.00E-44 |
| scaffold_3745:104360..105067 | 649 | predicted protein [Nematostella vectensis] | 4.00E-06 |
| scaffold_3743:89945..90421 | 477 | hypothetical protein TTHERM_00837800 [Tetrahymena thermophila] | 6.00E-05 |
| scaffold_3743:107804..108182 | 379 | hypothetical protein TTHERM_00837800 [Tetrahymena thermophila] | 5.00E-04 |
| scaffold_3742:234254..234848 | 595 | dynein light chain 8-like C [Tetrahymena thermophila] | 7.00E-43 |
| scaffold_3740:167198..168256 | 1059 | hypothetical protein TTHERM_00777140 [Tetrahymena thermophila] | 9.00E-28 |
| scaffold_3739:120474..124726 | 3979 | Zinc carboxypeptidase family protein [Tetrahymena thermophila] | 5.00E-71 |
| scaffold_3739:119510..124726 | 5164 | Zinc carboxypeptidase family protein [Tetrahymena thermophila] | 1.00E-78 |
| scaffold_3739:118874..120368 | 1269 | Zinc carboxypeptidase family protein [Tetrahymena thermophila] | 7.00E-43 |
| scaffold_3738:246116..250019 | 2555 | hypothetical protein TTHERM_00561180 [Tetrahymena thermophila] | 5.00E-08 |
| scaffold_3737:221816..224619 | 2804 | hypothetical protein [Paramecium tetraurelia strain d4-2] | 9.00E-30 |
| scaffold_3737:219254..230947 | 9865 | PREDICTED: similar to blue cheese CG14001-PA [Acyrthosiphon pisum] | 2.00E-40 |
| scaffold_3736:430..1088 | 659 | ribose-5-phosphate isomerase B [Finegoldia magna BVS033A4] | 2.00E-13 |
| scaffold_3736:309222..310999 | 558 | hypothetical protein TTHERM_01222460 [Tetrahymena thermophila] | 2.00E-34 |
| scaffold_3735:372028..372924 | 897 | hypothetical protein TTHERM_00155310 [Tetrahymena thermophila] | 8.00E-21 |
| scaffold_3734:738..3224 | 1867 | hypothetical protein [Paramecium tetraurelia strain d4-2] | 5.00E-07 |
| scaffold_3734:59212..60167 | 956 | hypothetical protein TTHERM_00275830 [Tetrahymena thermophila] | 2.00E-12 |
| scaffold_3734:328384..330053 | 1338 | hypothetical protein SELMODRAFT_427287 [Selaginella moellendorffii] | 1.00E-23 |
| scaffold_3733:37408..38125 | 718 | ubiquitin [Clavispora lusitaniae ATCC 42720] | 6.00E-06 |
| scaffold_3732:328006..328954 | 554 | snRNP protein Lsm5, putative [Toxoplasma gondii ME49] | 7.00E-22 |
| scaffold_3732:167430..169199 | 1542 | hypothetical protein [Paramecium tetraurelia strain d4-2] | 6.00E-69 |
| scaffold_3730:36986..40282 | 2151 | Protein kinase domain containing protein [Tetrahymena thermophila] | 3.00E-66 |
| scaffold_3730:2466..3321 | 856 | hypothetical protein TTHERM_02315920 [Tetrahymena thermophila] | 7.00E-10 |
| scaffold_3728:94672..95003 | 332 | hypothetical protein TTHERM_00538500 [Tetrahymena thermophila] | 2.00E-37 |
| scaffold_3728:66568..67335 | 768 | hypothetical protein TTHERM_00538500 [Tetrahymena thermophila] | 1.00E-87 |
| scaffold_3728:55372..56520 | 1149 | hypothetical protein TTHERM_00538500 [Tetrahymena thermophila] | 1.00E-125 |
| scaffold_3728:408805..411291 | 1072 | hypothetical protein TTHERM_01256640 [Tetrahymena thermophila] | 1.00E-50 |
| scaffold_3728:281514..283185 | 1672 | hypothetical protein TTHERM_00540040 [Tetrahymena thermophila] | 1.00E-157 |
| scaffold_3728:280784..281304 | 404 | hypothetical protein TTHERM_00539000 [Tetrahymena thermophila] | 2.00E-27 |
| scaffold_3728:274584..277139 | 2440 | hypothetical protein TTHERM_00540040 [Tetrahymena thermophila] | 1.00E-153 |
| scaffold_3728:273336..274011 | 676 | hypothetical protein TTHERM_01017240 [Tetrahymena thermophila] | 4.00E-06 |
| scaffold_3727:63133..63740 | 608 | hypothetical protein TTHERM_00624200 [Tetrahymena thermophila] | 3.00E-10 |
| scaffold_3727:21361..21778 | 418 | hypothetical protein TTHERM_00214860 [Tetrahymena thermophila] | 3.00E-27 |
| scaffold_3727:19785..21072 | 1231 | hypothetical protein TTHERM_00363170 [Tetrahymena thermophila] | 3.00E-37 |
| scaffold_3727:18147..19548 | 1246 | hypothetical protein TTHERM_00363170 [Tetrahymena thermophila] | 1.00E-109 |
| scaffold_3727:175682..176259 | 578 | hypothetical protein TTHERM_00224540 [Tetrahymena thermophila] | 5.00E-10 |
| scaffold_3726:161184..164953 | 3188 | hypothetical protein TTHERM_00490570 [Tetrahymena thermophila] | 2.00E-45 |
| scaffold_3726:14076..14653 | 578 | J immobilization antigen, putative [Tetrahymena thermophila] | 4.00E-82 |
| scaffold_3726:112037..112923 | 795 | hypothetical protein TTHERM_00809260 [Tetrahymena thermophila] | 1.00E-105 |
| scaffold_3726:111141..111861 | 619 | hypothetical protein TTHERM_00809250 [Tetrahymena thermophila] | 2.00E-48 |
| scaffold_3725:67492..67842 | 351 | hypothetical protein TTHERM_00284210 [Tetrahymena thermophila] | 3.00E-28 |
| scaffold_3724:356940..357481 | 542 | hypothetical protein [Paramecium tetraurelia strain d4-2] | 6.00E-07 |
| scaffold_3724:342328..344418 | 1452 | hypothetical protein TTHERM_00348720 [Tetrahymena thermophila] | 1.00E-09 |
| scaffold_3724:23166..23966 | 656 | PREDICTED: hypothetical protein [Vitis vinifera] | 8.00E-07 |
| scaffold_3724:197322..199175 | 1670 | hypothetical protein TTHERM_01132880 [Tetrahymena thermophila] | 1.00E-134 |
| scaffold_3723:48493..51613 | 2598 | Piwi domain containing protein [Tetrahymena thermophila] | 0 |
| scaffold_3723:359516..360166 | 431 | hypothetical protein TTHERM_00518480 [Tetrahymena thermophila] | 4.00E-21 |
| scaffold_3723:335320..335903 | 301 | hypothetical protein TTHERM_02579220 [Tetrahymena thermophila] | 1.00E-12 |
| scaffold_3723:252768..255141 | 1167 | hypothetical protein TTHERM_00204070 [Tetrahymena thermophila] | 8.00E-12 |
| scaffold_3723:233421..234236 | 816 | Ferric reductase like transmembrane component family protein [Tetrahymena thermophila] | 1.00E-100 |
| scaffold_3723:229015..231029 | 1845 | conserved hypothetical protein [Tetrahymena thermophila] | 0 |
| scaffold_3723:225649..226387 | 739 | Ferric reductase like transmembrane component family protein [Tetrahymena thermophila] | 1.00E-112 |
| scaffold_3723:214983..215824 | 842 | Ferric reductase like transmembrane component family protein [Tetrahymena thermophila] | 1.00E-100 |
| scaffold_3723:17895..19185 | 389 | hypothetical protein TTHERM_00805810 [Tetrahymena thermophila] | 8.00E-23 |
| scaffold_3723:127323..127802 | 480 | hypothetical protein TTHERM_00509050 [Tetrahymena thermophila] | 6.00E-26 |
| scaffold_3722:464..1565 | 941 | histidine triad (hit) protein member, putative [Ixodes scapularis] | 5.00E-40 |
| scaffold_3722:352617..353464 | 788 | hypothetical protein [Paramecium tetraurelia strain d4-2] | 9.00E-12 |
| scaffold_3722:350384..352427 | 1624 | Bowman-Birk serine protease inhibitor family protein [Tetrahymena thermophila] | 3.00E-43 |
| scaffold_3722:345082..346773 | 1692 | Bowman-Birk serine protease inhibitor family protein [Tetrahymena thermophila] | 3.00E-22 |
| scaffold_3722:342777..348536 | 5140 | Bowman-Birk serine protease inhibitor family protein [Tetrahymena thermophila] | 1.00E-84 |
| scaffold_3722:342777..344857 | 2025 | hypothetical protein TTHERM_01098980 [Tetrahymena thermophila] | 6.00E-30 |
| scaffold_3722:271453..272271 | 648 | hypothetical protein TTHERM_01017240 [Tetrahymena thermophila] | 2.00E-21 |
| scaffold_3722:268472..270601 | 1440 | hypothetical protein TTHERM_01017240 [Tetrahymena thermophila] | 5.00E-20 |
| scaffold_3722:266971..267594 | 464 | hypothetical protein TTHERM_01535640 [Tetrahymena thermophila] | 5.00E-07 |
| scaffold_3721:417553..418119 | 567 | hypothetical protein TTHERM_01317420 [Tetrahymena thermophila] | 1.00E-16 |
| scaffold_3721:307268..309166 | 1772 | hypothetical protein TTHERM_00992990 [Tetrahymena thermophila] | 7.00E-16 |
| scaffold_3720:22339..23775 | 495 | hypothetical protein TTHERM_01256640 [Tetrahymena thermophila] | 3.00E-09 |
| scaffold_372:20646..21585 | 556 | hypothetical protein TTHERM_01372790 [Tetrahymena thermophila] | 2.00E-32 |
| scaffold_3719:522889..524139 | 1251 | hypothetical protein TTHERM_00079678 [Tetrahymena thermophila] | 7.00E-04 |
| scaffold_3719:390428..393014 | 2039 | hypothetical protein TTHERM_00482390 [Tetrahymena thermophila] | 0 |
| scaffold_3719:386319..390126 | 3808 | hypothetical protein TTHERM_00481371 [Tetrahymena thermophila] | 0 |
| scaffold_3719:383560..384208 | 649 | hypothetical protein TTHERM_00482390 [Tetrahymena thermophila] | 5.00E-53 |
| scaffold_3719:252214..252827 | 614 | hypothetical protein TTHERM_00478070 [Tetrahymena thermophila] | 1.00E-116 |
| scaffold_3719:251222..252099 | 641 | hypothetical protein TTHERM_00478070 [Tetrahymena thermophila] | 1.00E-109 |
| scaffold_3719:250502..251077 | 576 | hypothetical protein TTHERM_00478070 [Tetrahymena thermophila] | 5.00E-95 |
| scaffold_3719:191897..193112 | 1216 | hypothetical protein TTHERM_00476970 [Tetrahymena thermophila] | 1.00E-120 |
| scaffold_3718:304336..305385 | 798 | hypothetical protein TTHERM_00155310 [Tetrahymena thermophila] | 2.00E-23 |
| scaffold_3718:243385..244360 | 976 | hypothetical protein TTHERM_00442870 [Tetrahymena thermophila] | 2.00E-34 |
| scaffold_3717:82909..84647 | 1151 | LOC495678 protein [Xenopus laevis] | 6.00E-04 |
| scaffold_3715:515012..515578 | 567 | NUDIX family hydrolase [Clostridiales genomosp. BVAB3 str. UPII9-5] | 3.00E-07 |
| scaffold_3714:9958..17225 | 6285 | Dynein heavy chain family protein [Tetrahymena thermophila] | 1.00E-108 |
| scaffold_3714:81214..82898 | 1685 | hypothetical protein TTHERM_00346580 [Tetrahymena thermophila] | 7.00E-04 |
| scaffold_3714:704..9856 | 7721 | hypothetical protein TTHERM_01106080 [Tetrahymena thermophila] | 5.00E-78 |
| scaffold_3714:22745..24948 | 1639 | Dynein heavy chain family protein [Tetrahymena thermophila] | 4.00E-25 |
| scaffold_3714:21542..22595 | 946 | Dynein heavy chain family protein [Tetrahymena thermophila] | 3.00E-09 |
| scaffold_3714:19624..20436 | 701 | hypothetical protein TTHERM_01106080 [Tetrahymena thermophila] | 2.00E-28 |
| scaffold_3714:17816..19420 | 1382 | hypothetical protein TTHERM_01106080 [Tetrahymena thermophila] | 7.00E-52 |
| scaffold_3713:544320..545827 | 1443 | tRNA-guanine transglycosylase family protein [Tetrahymena thermophila] | 1.00E-112 |
| scaffold_3713:318931..319323 | 393 | hypothetical protein TTHERM_00401929 [Tetrahymena thermophila] | 3.00E-30 |
| scaffold_3713:315602..317874 | 2150 | hypothetical protein TTHERM_00401920 [Tetrahymena thermophila] | 0 |
| scaffold_3713:315602..317245 | 1517 | hypothetical protein TTHERM_00401920 [Tetrahymena thermophila] | 1.00E-127 |
| scaffold_3713:313989..314770 | 711 | hypothetical protein TTHERM_00401920 [Tetrahymena thermophila] | 5.00E-24 |
| scaffold_3713:313295..313720 | 426 | hypothetical protein TTHERM_00401920 [Tetrahymena thermophila] | 3.00E-24 |
| scaffold_3713:309320..311534 | 2068 | hypothetical protein TTHERM_00401920 [Tetrahymena thermophila] | 1.00E-105 |
| scaffold_3713:273631..274472 | 842 | hypothetical protein TTHERM_00819440 [Tetrahymena thermophila] | 6.00E-76 |
| scaffold_3713:164790..165703 | 789 | hypothetical protein TTHERM_00647490 [Tetrahymena thermophila] | 3.00E-74 |
| scaffold_3711:516849..517435 | 587 | hypothetical protein [Paramecium tetraurelia strain d4-2] | 5.00E-08 |
| scaffold_3710:98102..99005 | 403 | hypothetical protein TTHERM_00278640 [Tetrahymena thermophila] | 1.00E-05 |
| scaffold_3710:96451..97494 | 359 | hypothetical protein TTHERM_00862730 [Tetrahymena thermophila] | 7.00E-27 |
| scaffold_3710:478095..478463 | 369 | hypothetical protein TTHERM_00469260 [Tetrahymena thermophila] | 2.00E-09 |
| scaffold_3710:470079..470649 | 571 | hypothetical protein TTHERM_00469260 [Tetrahymena thermophila] | 3.00E-12 |
| scaffold_3710:103243..104634 | 423 | hypothetical protein TTHERM_00467220 [Tetrahymena thermophila] | 2.00E-22 |
| scaffold_3708:125250..128809 | 3159 | Protein kinase domain containing protein [Tetrahymena thermophila] | 6.00E-08 |
| scaffold_3707:720549..721598 | 1050 | hypothetical protein TTHERM_00059520 [Tetrahymena thermophila] | 8.00E-82 |
| scaffold_3707:718997..719658 | 662 | hypothetical protein TTHERM_00059530 [Tetrahymena thermophila] | 3.00E-52 |
| scaffold_3707:1242925..1243357 | 433 | hypothetical protein TTHERM_00058210 [Tetrahymena thermophila] | 6.00E-31 |
| scaffold_3706:807102..807980 | 441 | hypothetical protein TTHERM_00123729 [Tetrahymena thermophila] | 2.00E-05 |
| scaffold_3706:617775..619575 | 1801 | hypothetical protein TTHERM_00698730 [Tetrahymena thermophila] | 6.00E-21 |
| scaffold_3705:99195..99644 | 450 | C1-like domain containing protein [Tetrahymena thermophila] | 2.00E-14 |
| scaffold_3705:565592..566790 | 1199 | hypothetical protein TTHERM_00365320 [Tetrahymena thermophila] | 3.00E-28 |
| scaffold_3705:103780..106555 | 2776 | C1-like domain containing protein [Tetrahymena thermophila] | 8.00E-14 |
| scaffold_3705:103101..103659 | 559 | hypothetical protein TTHERM_00326870 [Tetrahymena thermophila] | 5.00E-05 |
| scaffold_3703:99516..100849 | 556 | hypothetical protein TTHERM_00113249 [Tetrahymena thermophila] | 7.00E-15 |
| scaffold_3703:98117..99384 | 418 | hypothetical protein TTHERM_01256640 [Tetrahymena thermophila] | 4.00E-23 |
| scaffold_3703:128831..131043 | 2160 | hypothetical protein TTHERM_00354650 [Tetrahymena thermophila] | 1.00E-142 |
| scaffold_3702:485768..486435 | 534 | hypothetical protein BRAFLDRAFT_214423 [Branchiostoma floridae] | 2.00E-05 |
| scaffold_3701:701457..702421 | 373 | hypothetical protein TTHERM_01392280 [Tetrahymena thermophila] | 1.00E-14 |
| scaffold_3701:532..2009 | 1478 | hypothetical protein [Paramecium tetraurelia strain d4-2] | 1.00E-06 |
| scaffold_3700:783773..784598 | 826 | Rab-family small GTPase RabX1F [Tetrahymena thermophila] | 6.00E-11 |
| scaffold_3700:616575..617024 | 450 | hypothetical protein TTHERM_00150050 [Tetrahymena thermophila] | 2.00E-22 |
| scaffold_3700:109462..110591 | 994 | hypothetical protein TTHERM_00148680 [Tetrahymena thermophila] | 3.00E-77 |
| scaffold_3699:91300..92777 | 673 | Neurohypophysial hormone, N-terminal Domain containing protein [Tetrahymena thermophila] | 2.00E-27 |
| scaffold_3698:995364..997799 | 1917 | RING finger related protein [Tetrahymena thermophila] | 1.00E-14 |
| scaffold_3698:965556..966595 | 732 | hypothetical protein TTHERM_01390270 [Tetrahymena thermophila] | 3.00E-05 |
| scaffold_3698:868421..869423 | 816 | hypothetical protein TTHERM_00133610 [Tetrahymena thermophila] | 1.00E-37 |
| scaffold_3698:727706..728504 | 561 | hypothetical protein TTHERM_00283580 [Tetrahymena thermophila] | 8.00E-43 |
| scaffold_3697:638742..639379 | 638 | hypothetical protein TTHERM_00977770 [Tetrahymena thermophila] | 8.00E-14 |
| scaffold_3697:634455..635180 | 726 | hypothetical protein TTHERM_01017240 [Tetrahymena thermophila] | 3.00E-19 |
| scaffold_3697:633432..634170 | 429 | hypothetical protein TTHERM_01659050 [Tetrahymena thermophila] | 1.00E-19 |
| scaffold_3696:70781..71653 | 726 | hypothetical protein TTHERM_00304250 [Tetrahymena thermophila] | 4.00E-70 |
| scaffold_3696:559490..561706 | 519 | hypothetical protein TTHERM_01674210 [Tetrahymena thermophila] | 2.00E-15 |
| scaffold_3696:516594..517694 | 1030 | polyubiquitin [Naegleria gruberi] gb|EFC48335.1| polyubiquitin [Naegleria gruberi] | 8.00E-04 |
| scaffold_3696:434234..435101 | 814 | hypothetical protein TTHERM_01586460 [Tetrahymena thermophila] | 1.00E-88 |
| scaffold_3696:406168..408987 | 2820 | hypothetical protein TTHERM_00310020 [Tetrahymena thermophila] | 2.00E-84 |
| scaffold_3695:526873..528686 | 1814 | hypothetical protein TTHERM_00346580 [Tetrahymena thermophila] | 5.00E-23 |
| scaffold_3695:499280..500153 | 460 | hypothetical protein TTHERM_01349950 [Tetrahymena thermophila] | 4.00E-08 |
| scaffold_3695:18395..19145 | 751 | hypothetical protein TTHERM_02279870 [Tetrahymena thermophila] | 2.00E-07 |
| scaffold_3695:136704..139971 | 1276 | hypothetical protein TTHERM_01535640 [Tetrahymena thermophila] | 9.00E-28 |
| scaffold_3694:78473..79368 | 896 | hypothetical protein TTHERM_00277300 [Tetrahymena thermophila] | 2.00E-80 |
| scaffold_3694:61153..63186 | 787 | hypothetical protein TTHERM_01674210 [Tetrahymena thermophila] | 3.00E-14 |
| scaffold_3694:350468..352095 | 1628 | Platelet-activating factor acetylhydrolase, plasma/intracellular isoform II family protein [Tetrahymena thermophila] | 1.00E-142 |
| scaffold_3694:168807..170979 | 2173 | hypothetical protein TTHERM_01017240 [Tetrahymena thermophila] | 1.00E-32 |
| scaffold_3694:166304..166947 | 644 | hypothetical protein TTHERM_00052070 [Tetrahymena thermophila] | 4.00E-41 |
| scaffold_3693:212910..214060 | 1151 | hypothetical protein TTHERM_00372560 [Tetrahymena thermophila] | 1.00E-57 |
| scaffold_3693:202377..203056 | 680 | hypothetical protein TTHERM_00372560 [Tetrahymena thermophila] | 5.00E-18 |
| scaffold_3693:200903..201444 | 542 | hypothetical protein TTHERM_00372560 [Tetrahymena thermophila] | 8.00E-39 |
| scaffold_3693:199120..199990 | 871 | hypothetical protein TTHERM_00372560 [Tetrahymena thermophila] | 1.00E-47 |
| scaffold_3692:722800..724275 | 1061 | hypothetical protein TTHERM_01046990 [Tetrahymena thermophila] | 3.00E-04 |
| scaffold_3692:679374..682232 | 2804 | macronuclear nucleoporin MacNup98B [Tetrahymena thermophila] | 0 |
| scaffold_3691:757960..759032 | 990 | hypothetical protein TTHERM_00259310 [Tetrahymena thermophila] | 2.00E-26 |
| scaffold_3691:618440..620249 | 1115 | hypothetical protein TTHERM_00895840 [Tetrahymena thermophila] | 5.00E-12 |
| scaffold_3691:205967..209091 | 2819 | Major Facilitator Superfamily protein [Tetrahymena thermophila] | 1.00E-157 |
| scaffold_3691:204839..205863 | 931 | Major Facilitator Superfamily protein [Tetrahymena thermophila] | 1.00E-101 |
| scaffold_3690:678139..678900 | 704 | hypothetical protein TTHERM_00275690 [Tetrahymena thermophila] | 2.00E-65 |
| scaffold_3690:676275..676791 | 517 | hypothetical protein TTHERM_00275690 [Tetrahymena thermophila] | 1.00E-49 |
| scaffold_3690:672305..672720 | 360 | hypothetical protein TTHERM_00275690 [Tetrahymena thermophila] | 3.00E-19 |
| scaffold_3690:661350..661755 | 406 | hypothetical protein TTHERM_00274680 [Tetrahymena thermophila] | 5.00E-15 |
| scaffold_3690:638270..639002 | 664 | hypothetical protein TTHERM_00274630 [Tetrahymena thermophila] | 2.00E-17 |
| scaffold_3690:404783..405562 | 477 | hypothetical protein TTHERM_01465230 [Tetrahymena thermophila] | 1.00E-11 |
| scaffold_3690:209820..210541 | 664 | hypothetical protein [Paramecium tetraurelia strain d4-2] | 5.00E-09 |
| scaffold_3689:275772..281727 | 2311 | hypothetical protein [Paramecium tetraurelia strain d4-2] | 1.00E-22 |
| scaffold_3688:76915..78395 | 1331 | hypothetical protein TTHERM_00297050 [Tetrahymena thermophila] | 1.00E-115 |
| scaffold_3688:74841..76811 | 1710 | hypothetical protein TTHERM_00253469 [Tetrahymena thermophila] | 1.00E-150 |
| scaffold_3688:337998..338428 | 431 | hypothetical protein TTHERM_00289070 [Tetrahymena thermophila] | 9.00E-09 |
| scaffold_3688:278289..279567 | 807 | hypothetical protein [Paramecium tetraurelia strain d4-2] | 1.00E-13 |
| scaffold_3687:45827..48605 | 2500 | hypothetical protein [Paramecium tetraurelia strain d4-2] | 2.00E-22 |
| scaffold_3684:197785..198695 | 911 | Integral membrane protein DUF6 [Tetrahymena thermophila] | 4.00E-46 |
| scaffold_3682:170681..171699 | 1019 | hypothetical protein TTHERM_00190980 [Tetrahymena thermophila] | 6.00E-60 |
| scaffold_3680:495736..499798 | 2963 | hypothetical protein [Paramecium tetraurelia strain d4-2] | 0 |
| scaffold_368:6164..6474 | 311 | hypothetical protein TTHERM_00743700 [Tetrahymena thermophila] | 3.00E-04 |
| scaffold_3677:150187..150853 | 667 | hypothetical protein [Paramecium tetraurelia strain d4-2] | 2.00E-49 |
| scaffold_3672:12296..13136 | 613 | hypothetical protein TTHERM_00225800 [Tetrahymena thermophila] | 8.00E-61 |
| scaffold_366:6137..7001 | 840 | hypothetical protein TTHERM_01411030 [Tetrahymena thermophila] | 3.00E-11 |
| scaffold_366:5167..5546 | 380 | hypothetical protein TTHERM_01546840 [Tetrahymena thermophila] | 9.00E-11 |
| scaffold_366:23760..24189 | 430 | hypothetical protein TTHERM_00990520 [Tetrahymena thermophila] | 1.00E-23 |
| scaffold_366:18718..19393 | 445 | hypothetical protein TTHERM_00990520 [Tetrahymena thermophila] | 1.00E-22 |
| scaffold_364:53..873 | 821 | hypothetical protein TTHERM_01389210 [Tetrahymena thermophila] | 1.00E-117 |
| scaffold_364:2262..2703 | 442 | hypothetical protein TTHERM_01553980 [Tetrahymena thermophila] | 3.00E-06 |
| scaffold_364:1017..1585 | 569 | hypothetical protein TTHERM_00813080 [Tetrahymena thermophila] | 2.00E-43 |
| scaffold_360:24373..25134 | 590 | hypothetical protein TTHERM_00625930 [Tetrahymena thermophila] | 1.00E-13 |
| scaffold_359:7949..8919 | 916 | hypothetical protein TTHERM_00721920 [Tetrahymena thermophila] | 7.00E-11 |
| scaffold_353:10025..10818 | 794 | hypothetical protein TTHERM_01472430 [Tetrahymena thermophila] | 6.00E-62 |
| scaffold_351:19746..21213 | 681 | hypothetical protein TTHERM_01023090 [Tetrahymena thermophila] | 2.00E-04 |
| scaffold_351:18276..19476 | 306 | hypothetical protein TTHERM_00630670 [Tetrahymena thermophila] | 2.00E-12 |
| scaffold_336:5506..9636 | 1514 | Protein kinase domain containing protein [Tetrahymena thermophila] | 1.00E-170 |
| scaffold_336:1363..4032 | 478 | hypothetical protein TTHERM_01453050 [Tetrahymena thermophila] | 5.00E-31 |
| scaffold_331:9402..10225 | 528 | hypothetical protein TTHERM_01598630 [Tetrahymena thermophila] | 1.00E-12 |
| scaffold_329:7960..8852 | 324 | hypothetical protein TTHERM_00166070 [Tetrahymena thermophila] | 1.00E-21 |
| scaffold_329:6195..7677 | 587 | hypothetical protein TTHERM_01476520 [Tetrahymena thermophila] | 4.00E-14 |
| scaffold_326:17679..19112 | 437 | hypothetical protein TTHERM_00166070 [Tetrahymena thermophila] | 6.00E-13 |
| scaffold_326:13980..15544 | 437 | hypothetical protein TTHERM_01079270 [Tetrahymena thermophila] | 1.00E-11 |
| scaffold_321:43106..43988 | 805 | hypothetical protein TTHERM_01537660 [Tetrahymena thermophila] | 1.00E-75 |
| scaffold_32:531600..532660 | 1061 | hypothetical protein TTHERM_00323160 [Tetrahymena thermophila] | 8.00E-53 |
| scaffold_319:48161..48613 | 453 | predicted protein [Naegleria gruberi] gb|EFC44916.1| predicted protein [Naegleria gruberi] | 1.00E-04 |
| scaffold_317:44920..45256 | 337 | hypothetical protein TTHERM_01021910 [Tetrahymena thermophila] | 2.00E-07 |
| scaffold_317:32968..34626 | 1659 | hypothetical protein TTHERM_01341610 [Tetrahymena thermophila] | 6.00E-30 |
| scaffold_316:49502..50105 | 325 | hypothetical protein TTHERM_02334970 [Tetrahymena thermophila] | 3.00E-05 |
| scaffold_315:14098..15016 | 919 | hypothetical protein [Paramecium tetraurelia strain d4-2] | 4.00E-15 |
| scaffold_305:51391..52618 | 713 | hypothetical protein TTHERM_00382449 [Tetrahymena thermophila] | 1.00E-06 |
| scaffold_302:33320..34369 | 1050 | hypothetical protein TTHERM_00948680 [Tetrahymena thermophila] | 9.00E-78 |
| scaffold_301:38209..39004 | 796 | hypothetical protein TTHERM_01266130 [Tetrahymena thermophila] | 1.00E-05 |
| scaffold_297:26081..29826 | 1208 | hypothetical protein TTHERM_01260710 [Tetrahymena thermophila] | 4.00E-43 |
| scaffold_296:24824..25285 | 462 | hypothetical protein TTHERM_01259670 [Tetrahymena thermophila] | 4.00E-22 |
| scaffold_295:56427..56862 | 436 | hypothetical protein TTHERM_00990520 [Tetrahymena thermophila] | 2.00E-16 |
| scaffold_291:60504..61393 | 890 | hypothetical protein TTHERM_00990520 [Tetrahymena thermophila] | 5.00E-31 |
| scaffold_291:16..778 | 763 | hypothetical protein TTHERM_00990520 [Tetrahymena thermophila] | 3.00E-33 |
| scaffold_290:58395..59533 | 584 | hypothetical protein TTHERM_00166070 [Tetrahymena thermophila] | 7.00E-21 |
| scaffold_288:3568..4051 | 484 | hypothetical protein TTHERM_00805800 [Tetrahymena thermophila] | 4.00E-18 |
| scaffold_287:51076..52539 | 595 | hypothetical protein TTHERM_01674210 [Tetrahymena thermophila] | 1.00E-12 |
| scaffold_286:35877..36745 | 315 | hypothetical protein TTHERM_00746910 [Tetrahymena thermophila] | 2.00E-15 |
| scaffold_285:52238..53593 | 453 | hypothetical protein TTHERM_01256620 [Tetrahymena thermophila] | 2.00E-09 |
| scaffold_285:47151..48207 | 649 | hypothetical protein [Paramecium tetraurelia strain d4-2] | 2.00E-48 |
| scaffold_283:32052..32812 | 761 | hypothetical protein TTHERM_00625930 [Tetrahymena thermophila] | 1.00E-34 |
| scaffold_283:25803..26444 | 642 | hypothetical protein TTHERM_01716390 [Tetrahymena thermophila] | 3.00E-19 |
| scaffold_281:57204..57661 | 458 | hypothetical protein TTHERM_01225690 [Tetrahymena thermophila] | 8.00E-34 |
| scaffold_281:54644..55539 | 896 | hypothetical protein TTHERM_00990520 [Tetrahymena thermophila] | 6.00E-32 |
| scaffold_280:64269..64710 | 442 | hypothetical protein TTHERM_00990520 [Tetrahymena thermophila] | 1.00E-26 |
| scaffold_279:8105..9898 | 573 | conserved hypothetical protein [Tetrahymena thermophila] | 2.00E-23 |
| scaffold_279:10328..15388 | 3350 | hypothetical protein [Paramecium tetraurelia strain d4-2] | 3.00E-14 |
| scaffold_278:62190..63155 | 966 | hypothetical protein TTHERM_01598630 [Tetrahymena thermophila] | 1.00E-19 |
| scaffold_274:17..1658 | 1217 | hypothetical protein TTHERM_00248531 [Tetrahymena thermophila] | 2.00E-04 |
| scaffold_267:31899..32276 | 378 | hypothetical protein TTHERM_00990520 [Tetrahymena thermophila] | 2.00E-14 |
| scaffold_266:2017..2456 | 440 | hypothetical protein TTHERM_00625930 [Tetrahymena thermophila] | 5.00E-27 |
| scaffold_263:609..1320 | 487 | Protein kinase domain containing protein [Tetrahymena thermophila] | 1.00E-13 |
| scaffold_263:53148..54326 | 425 | hypothetical protein TTHERM_01476520 [Tetrahymena thermophila] | 3.00E-10 |
| scaffold_263:36635..37662 | 480 | hypothetical protein TTHERM_01288990 [Tetrahymena thermophila] | 6.00E-12 |
| scaffold_263:15457..16003 | 363 | Protein kinase domain containing protein [Tetrahymena thermophila] | 4.00E-53 |
| scaffold_263:14089..14542 | 454 | Protein kinase domain containing protein [Tetrahymena thermophila] | 3.00E-39 |
| scaffold_263:11831..15344 | 1482 | Protein kinase domain containing protein [Tetrahymena thermophila] | 1.00E-179 |
| scaffold_263:10796..12573 | 880 | Protein kinase domain containing protein [Tetrahymena thermophila] | 7.00E-53 |
| scaffold_257:9041..10531 | 606 | Protein kinase domain containing protein [Tetrahymena thermophila] | 1.00E-103 |
| scaffold_257:12251..14182 | 1075 | Protein kinase domain containing protein [Tetrahymena thermophila] | 1.00E-121 |
| scaffold_255:82323..83102 | 780 | hypothetical protein TTHERM_01484660 [Tetrahymena thermophila] | 8.00E-31 |
| scaffold_255:12007..13036 | 1030 | hypothetical protein TTHERM_00365470 [Tetrahymena thermophila] | 4.00E-48 |
| scaffold_245:67277..68082 | 729 | hypothetical protein TTHERM_00989380 [Tetrahymena thermophila] | 5.00E-40 |
| scaffold_243:45054..46221 | 435 | cyclic nucleotide-binding domain containing protein [Tetrahymena thermophila] | 1.00E-14 |
| scaffold_242:7118..7725 | 331 | Protein kinase domain containing protein [Tetrahymena thermophila] | 8.00E-15 |
| scaffold_241:76415..76807 | 393 | hypothetical protein TTHERM_01407980 [Tetrahymena thermophila] | 3.00E-08 |
| scaffold_241:74479..74949 | 471 | hypothetical protein TTHERM_00990520 [Tetrahymena thermophila] | 2.00E-33 |
| scaffold_234:42159..42939 | 366 | hypothetical protein TTHERM_00113249 [Tetrahymena thermophila] | 1.00E-09 |
| scaffold_23:751702..752517 | 816 | hypothetical protein TTHERM_00990520 [Tetrahymena thermophila] | 9.00E-10 |
| scaffold_23:696950..700745 | 3796 | hypothetical protein TTHERM_00346580 [Tetrahymena thermophila] | 3.00E-04 |
| scaffold_23:692504..694267 | 1764 | hypothetical protein TTHERM_00695678 [Tetrahymena thermophila] | 1.00E-07 |
| scaffold_23:60..708 | 649 | hypothetical protein TTHERM_01317390 [Tetrahymena thermophila] | 4.00E-58 |
| scaffold_23:39000..40576 | 1577 | hypothetical protein TTHERM_00554300 [Tetrahymena thermophila] | 1.00E-12 |
| scaffold_23:1721..2117 | 343 | hypothetical protein TTHERM_00895560 [Tetrahymena thermophila] | 7.00E-06 |
| scaffold_23:1211..1575 | 365 | hypothetical protein TTHERM_01405890 [Tetrahymena thermophila] | 9.00E-19 |
| scaffold_227:96033..97341 | 1309 | hypothetical protein TTHERM_00001410 [Tetrahymena thermophila] | 2.00E-29 |
| scaffold_227:8345..9356 | 956 | hypothetical protein TTHERM_02474150 [Tetrahymena thermophila] | 3.00E-16 |
| scaffold_227:7051..8124 | 572 | hypothetical protein TTHERM_02474150 [Tetrahymena thermophila] | 2.00E-06 |
| scaffold_227:7051..7898 | 792 | hypothetical protein TTHERM_02474150 [Tetrahymena thermophila] | 3.00E-16 |
| scaffold_227:64360..64870 | 511 | hypothetical protein TTHERM_00990520 [Tetrahymena thermophila] | 2.00E-26 |
| scaffold_22:730533..731411 | 573 | hypothetical protein TTHERM_00714630 [Tetrahymena thermophila] | 4.00E-21 |
| scaffold_22:611146..612284 | 1139 | hypothetical protein TTHERM_02579220 [Tetrahymena thermophila] | 6.00E-11 |
| scaffold_216:26865..27451 | 363 | hypothetical protein TTHERM_00166070 [Tetrahymena thermophila] | 6.00E-05 |
| scaffold_216:18536..20129 | 802 | CARD15-like protein, putative [Tetrahymena thermophila] | 1.00E-04 |
| scaffold_214:76627..77149 | 523 | hypothetical protein TTHERM_02554200 [Tetrahymena thermophila] | 2.00E-14 |
| scaffold_214:31401..32016 | 403 | hypothetical protein TTHERM_00749020 [Tetrahymena thermophila] | 5.00E-17 |
| scaffold_214:30794..31224 | 431 | hypothetical protein TTHERM_00625930 [Tetrahymena thermophila] | 3.00E-23 |
| scaffold_214:25798..26696 | 899 | hypothetical protein TTHERM_00625930 [Tetrahymena thermophila] | 8.00E-34 |
| scaffold_214:121876..122285 | 410 | hypothetical protein TTHERM_01673190 [Tetrahymena thermophila] | 3.00E-25 |
| scaffold_213:40781..41155 | 375 | hypothetical protein TTHERM_00343480 [Tetrahymena thermophila] | 6.00E-13 |
| scaffold_210:84381..85287 | 907 | hypothetical protein TTHERM_01198170 [Tetrahymena thermophila] | 5.00E-63 |
| scaffold_210:105569..105901 | 333 | hypothetical protein TTHERM_01198170 [Tetrahymena thermophila] | 9.00E-09 |
| scaffold_21:737078..740394 | 3150 | hypothetical protein TTHERM_00248290 [Tetrahymena thermophila] | 0 |
| scaffold_21:644304..645654 | 1192 | hypothetical protein TTHERM_00247130 [Tetrahymena thermophila] | 4.00E-48 |
| scaffold_21:53460..53898 | 439 | hypothetical protein TTHERM_00973020 [Tetrahymena thermophila] | 3.00E-26 |
| scaffold_205:93151..93797 | 570 | hypothetical protein [Paramecium tetraurelia strain d4-2] | 1.00E-08 |
| scaffold_205:90132..91055 | 924 | hypothetical protein TTHERM_00943060 [Tetrahymena thermophila] | 8.00E-19 |
| scaffold_205:681..1875 | 1118 | hypothetical protein [Paramecium tetraurelia strain d4-2] | 1.00E-15 |
| scaffold_205:50538..51997 | 648 | hypothetical protein TTHERM_01296400 [Tetrahymena thermophila] | 3.00E-16 |
| scaffold_204:111566..113402 | 571 | hypothetical protein TTHERM_01476520 [Tetrahymena thermophila] | 5.00E-19 |
| scaffold_204:109400..110029 | 630 | hypothetical protein TTHERM_01595620 [Tetrahymena thermophila] | 1.00E-14 |
| scaffold_202:16104..16963 | 434 | conserved hypothetical protein [Tetrahymena thermophila] | 3.00E-13 |
| scaffold_201:82793..83562 | 770 | hypothetical protein TTHERM_01484660 [Tetrahymena thermophila] | 4.00E-29 |
| scaffold_201:7972..8401 | 430 | hypothetical protein TTHERM_00990520 [Tetrahymena thermophila] | 4.00E-29 |
| scaffold_201:60712..61032 | 321 | hypothetical protein TTHERM_01023000 [Tetrahymena thermophila] | 1.00E-28 |
| scaffold_201:46717..48084 | 475 | hypothetical protein TTHERM_00625930 [Tetrahymena thermophila] | 6.00E-39 |
| scaffold_201:45905..46391 | 487 | hypothetical protein TTHERM_01673190 [Tetrahymena thermophila] | 4.00E-27 |
| scaffold_201:43471..43982 | 512 | hypothetical protein TTHERM_00625930 [Tetrahymena thermophila] | 3.00E-35 |
| scaffold_201:130594..131260 | 458 | hypothetical protein TTHERM_00990520 [Tetrahymena thermophila] | 3.00E-24 |
| scaffold_201:110342..111573 | 315 | hypothetical protein TTHERM_01422360 [Tetrahymena thermophila] | 8.00E-10 |
| scaffold_199:130367..130848 | 482 | hypothetical protein TTHERM_00625930 [Tetrahymena thermophila] | 1.00E-19 |
| scaffold_199:120741..122202 | 1173 | hypothetical protein TTHERM_01018540 [Tetrahymena thermophila] | 1.00E-105 |
| scaffold_1968:2..980 | 979 | hypothetical protein TTHERM_01405890 [Tetrahymena thermophila] | 1.00E-124 |
| scaffold_1957:78..974 | 527 | Protein kinase domain containing protein [Tetrahymena thermophila] | 2.00E-76 |
| scaffold_195:81434..82065 | 632 | hypothetical protein TTHERM_01075650 [Tetrahymena thermophila] | 2.00E-20 |
| scaffold_193:40466..41235 | 770 | hypothetical protein TTHERM_01102720 [Tetrahymena thermophila] | 2.00E-11 |
| scaffold_193:37503..38306 | 804 | major facilitator superfamily protein [Tetrahymena thermophila] | 1.00E-15 |
| scaffold_193:292..1425 | 631 | conserved hypothetical protein [Tetrahymena thermophila] | 2.00E-35 |
| scaffold_19:584195..584695 | 501 | hypothetical protein TTHERM_01407980 [Tetrahymena thermophila] | 2.00E-22 |
| scaffold_19:284002..284314 | 313 | hypothetical protein TTHERM_00225850 [Tetrahymena thermophila] | 1.00E-11 |
| scaffold_187:34126..34707 | 438 | Ras family protein [Tetrahymena thermophila] | 5.00E-35 |
| scaffold_185:48352..49655 | 596 | hypothetical protein TTHERM_01716390 [Tetrahymena thermophila] | 4.00E-16 |
| scaffold_185:107132..107668 | 537 | hypothetical protein TTHERM_01673190 [Tetrahymena thermophila] | 1.00E-28 |
| scaffold_184:140425..141030 | 549 | hypothetical protein TTHERM_00986290 [Tetrahymena thermophila] | 9.00E-17 |
| scaffold_182:9192..11055 | 934 | hypothetical protein TTHERM_00698600 [Tetrahymena thermophila] | 2.00E-16 |
| scaffold_182:54139..54778 | 387 | hypothetical protein TTHERM_01017240 [Tetrahymena thermophila] | 4.00E-09 |
| scaffold_182:157140..157631 | 492 | hypothetical protein TTHERM_00625930 [Tetrahymena thermophila] | 4.00E-30 |
| scaffold_1813:7..779 | 715 | hypothetical protein TTHERM_01479590 [Tetrahymena thermophila] | 7.00E-07 |
| scaffold_181:171..937 | 767 | hypothetical protein TTHERM_00974070 [Tetrahymena thermophila] | 3.00E-61 |
| scaffold_181:145368..148812 | 2941 | hypothetical protein [Paramecium tetraurelia strain d4-2] | 2.00E-73 |
| scaffold_181:144124..145107 | 874 | hypothetical protein [Paramecium tetraurelia strain d4-2] | 7.00E-09 |
| scaffold_1803:239..911 | 673 | hypothetical protein TTHERM_00990520 [Tetrahymena thermophila] | 2.00E-29 |
| scaffold_179:30333..32846 | 1237 | Protein kinase domain containing protein [Tetrahymena thermophila] | 1.00E-139 |
| scaffold_179:28808..29145 | 338 | Protein kinase domain containing protein [Tetrahymena thermophila] | 4.00E-32 |
| scaffold_179:18754..19212 | 459 | hypothetical protein TTHERM_00734060 [Tetrahymena thermophila] | 7.00E-29 |
| scaffold_179:116961..118611 | 555 | hypothetical protein TTHERM_01256640 [Tetrahymena thermophila] | 4.00E-26 |
| scaffold_1789:364..845 | 482 | hypothetical protein TTHERM_00990520 [Tetrahymena thermophila] | 2.00E-41 |
| scaffold_177:70320..71824 | 361 | conserved hypothetical protein [Tetrahymena thermophila] | 5.00E-27 |
| scaffold_177:126655..127789 | 459 | hypothetical protein TTHERM_00954130 [Tetrahymena thermophila] | 4.00E-15 |
| scaffold_175:94773..96406 | 1634 | hypothetical protein TTHERM_00951860 [Tetrahymena thermophila] | 1.00E-111 |
| scaffold_173:163053..164175 | 626 | hypothetical protein TTHERM_02579220 [Tetrahymena thermophila] | 1.00E-10 |
| scaffold_173:158039..159188 | 333 | hypothetical protein TTHERM_00805810 [Tetrahymena thermophila] | 1.00E-28 |
| scaffold_1722:13..479 | 348 | PREDICTED: hypothetical protein LOC100503213, partial [Mus musculus] | 6.00E-05 |
| scaffold_172:6230..6917 | 688 | hypothetical protein TTHERM_01017240 [Tetrahymena thermophila] | 5.00E-25 |
| scaffold_172:559..1154 | 596 | hypothetical protein TTHERM_01484660 [Tetrahymena thermophila] | 2.00E-34 |
| scaffold_172:4380..4911 | 532 | hypothetical protein TTHERM_01017240 [Tetrahymena thermophila] | 2.00E-30 |
| scaffold_172:170347..170851 | 505 | hypothetical protein TTHERM_00990520 [Tetrahymena thermophila] | 3.00E-26 |
| scaffold_172:1364..1942 | 579 | hypothetical protein TTHERM_00990520 [Tetrahymena thermophila] | 4.00E-36 |
| scaffold_1718:185..687 | 503 | hypothetical protein TTHERM_02554200 [Tetrahymena thermophila] | 3.00E-26 |
| scaffold_170:143229..145580 | 1362 | hypothetical protein TTHERM_00209280 [Tetrahymena thermophila] | 2.00E-08 |
| scaffold_170:114747..115412 | 603 | hypothetical protein TTHERM_00197685 [Tetrahymena thermophila] | 5.00E-19 |
| scaffold_17:831444..832386 | 943 | hypothetical protein TTHERM_01017240 [Tetrahymena thermophila] | 5.00E-20 |
| scaffold_17:812454..814434 | 770 | conserved hypothetical protein [Tetrahymena thermophila] | 1.00E-26 |
| scaffold_17:269997..270544 | 315 | hypothetical protein TTHERM_00200530 [Tetrahymena thermophila] | 1.00E-11 |
| scaffold_169:77772..78971 | 908 | hypothetical protein TTHERM_02579220 [Tetrahymena thermophila] | 2.00E-12 |
| scaffold_1657:93..545 | 453 | hypothetical protein TTHERM_01017240 [Tetrahymena thermophila] | 2.00E-27 |
| scaffold_1647:296..825 | 530 | hypothetical protein TTHERM_01246660 [Tetrahymena thermophila] | 2.00E-28 |
| scaffold_162:65600..65958 | 359 | hypothetical protein TTHERM_00911120 [Tetrahymena thermophila] | 4.00E-13 |
| scaffold_162:166021..167906 | 1886 | hypothetical protein TTHERM_00913410 [Tetrahymena thermophila] | 1.00E-25 |
| scaffold_162:164819..165293 | 475 | hypothetical protein TTHERM_00913410 [Tetrahymena thermophila] | 2.00E-64 |
| scaffold_162:163706..164069 | 364 | hypothetical protein TTHERM_00913410 [Tetrahymena thermophila] | 9.00E-35 |
| scaffold_162:122523..122941 | 419 | Mago nashi protein [Tetrahymena thermophila] | 1.00E-06 |
| scaffold_161:136528..137171 | 451 | SLEI family protein [Tetrahymena thermophila] | 2.00E-04 |
| scaffold_161:13112..15587 | 1061 | Protein kinase domain containing protein [Tetrahymena thermophila] | 4.00E-24 |
| scaffold_161:11008..11479 | 472 | Protein kinase domain containing protein [Tetrahymena thermophila] | 1.00E-37 |
| scaffold_160:44855..45496 | 372 | hypothetical protein TTHERM_01222460 [Tetrahymena thermophila] | 2.00E-22 |
| scaffold_160:152210..152539 | 330 | hypothetical protein TTHERM_00364290 [Tetrahymena thermophila] | 1.00E-07 |
| scaffold_160:149359..150231 | 805 | hypothetical protein TTHERM_00899510 [Tetrahymena thermophila] | 2.00E-23 |
| scaffold_160:12464..12838 | 375 | hypothetical protein TTHERM_00625930 [Tetrahymena thermophila] | 4.00E-23 |
| scaffold_1573:248..891 | 644 | hypothetical protein TTHERM_01297400 [Tetrahymena thermophila] | 8.00E-59 |
| scaffold_1571:541..1041 | 501 | hypothetical protein TTHERM_01524430 [Tetrahymena thermophila] | 2.00E-10 |
| scaffold_1565:447..813 | 367 | hypothetical protein TTHERM_00625930 [Tetrahymena thermophila] | 3.00E-21 |
| scaffold_156:67090..67476 | 387 | hypothetical protein TTHERM_00343430 [Tetrahymena thermophila] | 4.00E-10 |
| scaffold_155:79893..80251 | 359 | hypothetical protein TTHERM_01599640 [Tetrahymena thermophila] | 2.00E-10 |
| scaffold_1536:825..1128 | 304 | hypothetical protein TTHERM_00790970 [Tetrahymena thermophila] | 6.00E-31 |
| scaffold_153:177157..177904 | 491 | hypothetical protein TTHERM_02103570 [Tetrahymena thermophila] | 8.00E-07 |
| scaffold_1526:399..1126 | 410 | hypothetical protein TTHERM_01697320 [Tetrahymena thermophila] | 6.00E-05 |
| scaffold_1510:385..1108 | 461 | hypothetical protein TTHERM_02247830 [Tetrahymena thermophila] | 3.00E-28 |
| scaffold_151:93369..94928 | 1067 | hypothetical protein TTHERM_00862730 [Tetrahymena thermophila] | 4.00E-11 |
| scaffold_151:70191..71614 | 1328 | hypothetical protein TTHERM_00854410 [Tetrahymena thermophila] | 1.00E-28 |
| scaffold_151:41728..42812 | 948 | hypothetical protein TTHERM_00112900 [Tetrahymena thermophila] | 1.00E-23 |
| scaffold_151:203488..204199 | 378 | SLEI family protein [Tetrahymena thermophila] | 3.00E-13 |
| scaffold_151:165402..166839 | 465 | hypothetical protein TTHERM_00611560 [Tetrahymena thermophila] | 1.00E-20 |
| scaffold_151:162259..163533 | 1275 | hypothetical protein TTHERM_00990520 [Tetrahymena thermophila] | 4.00E-18 |
| scaffold_151:109429..109771 | 343 | hypothetical protein TTHERM_01595620 [Tetrahymena thermophila] | 9.00E-12 |
| scaffold_151:106650..107079 | 430 | hypothetical protein TTHERM_01234330 [Tetrahymena thermophila] | 2.00E-05 |
| scaffold_151:105426..105862 | 437 | hypothetical protein TTHERM_01535640 [Tetrahymena thermophila] | 8.00E-15 |
| scaffold_151:103459..104655 | 450 | hypothetical protein TTHERM_00805810 [Tetrahymena thermophila] | 4.00E-25 |
| scaffold_148:6038..6841 | 587 | hypothetical protein TTHERM_00085640 [Tetrahymena thermophila] | 7.00E-11 |
| scaffold_145:693..2903 | 1286 | hypothetical protein TTHERM_00094090 [Tetrahymena thermophila] | 1.00E-06 |
| scaffold_145:173331..173930 | 600 | hypothetical protein TTHERM_01484660 [Tetrahymena thermophila] | 3.00E-07 |
| scaffold_145:166358..166874 | 517 | hypothetical protein TTHERM_00122389 [Tetrahymena thermophila] | 3.00E-04 |
| scaffold_1442:32..407 | 320 | hypothetical protein TTHERM_01504080 [Tetrahymena thermophila] | 6.00E-07 |
| scaffold_144:793..2101 | 1098 | hypothetical protein TTHERM_01587480 [Tetrahymena thermophila] | 3.00E-25 |
| scaffold_144:224364..224834 | 471 | hypothetical protein TTHERM_00625930 [Tetrahymena thermophila] | 4.00E-33 |
| scaffold_14:902411..902808 | 398 | hypothetical protein TTHERM_01598630 [Tetrahymena thermophila] | 8.00E-16 |
| scaffold_139:178479..178915 | 437 | conserved hypothetical protein [Tetrahymena thermophila] | 4.00E-07 |
| scaffold_138:60508..61478 | 971 | hypothetical protein TTHERM_01198170 [Tetrahymena thermophila] | 1.00E-48 |
| scaffold_138:37297..38507 | 1211 | hypothetical protein TTHERM_01198170 [Tetrahymena thermophila] | 1.00E-85 |
| scaffold_138:36092..36506 | 415 | hypothetical protein TTHERM_00076980 [Tetrahymena thermophila] | 7.00E-08 |
| scaffold_137:177444..177875 | 432 | hypothetical protein TTHERM_00625930 [Tetrahymena thermophila] | 1.00E-19 |
| scaffold_137:176142..177025 | 884 | hypothetical protein TTHERM_00990520 [Tetrahymena thermophila] | 3.00E-28 |
| scaffold_135:80032..81917 | 1886 | hypothetical protein TTHERM_00554300 [Tetrahymena thermophila] | 3.00E-04 |
| scaffold_135:45338..46106 | 497 | hypothetical protein TTHERM_01674210 [Tetrahymena thermophila] | 3.00E-08 |
| scaffold_135:28529..29643 | 859 | hypothetical protein TTHERM_01484660 [Tetrahymena thermophila] | 2.00E-36 |
| scaffold_1334:763..1201 | 439 | hypothetical protein TTHERM_01484660 [Tetrahymena thermophila] | 6.00E-27 |
| scaffold_133:127104..127657 | 361 | hypothetical protein TTHERM_00209280 [Tetrahymena thermophila] | 2.00E-04 |
| scaffold_1323:430..1132 | 703 | hypothetical protein TTHERM_00990520 [Tetrahymena thermophila] | 2.00E-45 |
| scaffold_1301:163..713 | 551 | hypothetical protein TTHERM_02379080 [Tetrahymena thermophila] | 3.00E-07 |
| scaffold_129:203766..204077 | 312 | hypothetical protein TTHERM_01062890 [Tetrahymena thermophila] | 1.00E-04 |
| scaffold_127:97758..99916 | 1125 | hypothetical protein TTHERM_01256640 [Tetrahymena thermophila] | 1.00E-32 |
| scaffold_1245:632..968 | 337 | hypothetical protein TTHERM_01673190 [Tetrahymena thermophila] | 1.00E-24 |
| scaffold_122:259820..260989 | 658 | hypothetical protein TTHERM_00698600 [Tetrahymena thermophila] | 2.00E-11 |
| scaffold_122:246671..247074 | 404 | hypothetical protein TTHERM_00753670 [Tetrahymena thermophila] | 3.00E-21 |
| scaffold_122:212917..213477 | 561 | hypothetical protein TTHERM_00835430 [Tetrahymena thermophila] | 1.00E-06 |
| scaffold_122:210309..210839 | 531 | hypothetical protein TTHERM_00695690 [Tetrahymena thermophila] | 2.00E-05 |
| scaffold_122:117627..118240 | 325 | hypothetical protein TTHERM_01535640 [Tetrahymena thermophila] | 2.00E-15 |
| scaffold_121:6532..7021 | 490 | hypothetical protein TTHERM_00740570 [Tetrahymena thermophila] | 2.00E-34 |
| scaffold_121:44362..44791 | 430 | hypothetical protein TTHERM_00625930 [Tetrahymena thermophila] | 1.00E-20 |
| scaffold_121:42436..43053 | 347 | hypothetical protein TTHERM_01535640 [Tetrahymena thermophila] | 4.00E-22 |
| scaffold_121:211846..212810 | 965 | hypothetical protein TTHERM_00749020 [Tetrahymena thermophila] | 2.00E-93 |
| scaffold_121:197324..197641 | 318 | hypothetical protein TTHERM_01546840 [Tetrahymena thermophila] | 3.00E-13 |
| scaffold_121:16881..17726 | 776 | hypothetical protein TTHERM_01058760 [Tetrahymena thermophila] | 5.00E-65 |
| scaffold_121:159291..159652 | 362 | hypothetical protein TTHERM_00734010 [Tetrahymena thermophila] | 2.00E-26 |
| scaffold_121:120818..121850 | 717 | hypothetical protein TTHERM_01190460 [Tetrahymena thermophila] | 3.00E-11 |
| scaffold_120:99362..99728 | 367 | hypothetical protein TTHERM_01673190 [Tetrahymena thermophila] | 1.00E-15 |
| scaffold_119:67922..68670 | 749 | Protein kinase domain containing protein [Tetrahymena thermophila] | 1.00E-09 |
| scaffold_112:77153..78092 | 673 | hypothetical protein TTHERM_00644670 [Tetrahymena thermophila] | 3.00E-07 |
| scaffold_110:155811..156288 | 418 | hypothetical protein TTHERM_00085550 [Tetrahymena thermophila] | 3.00E-08 |
| scaffold_109:64374..64937 | 564 | hypothetical protein TTHERM_01237400 [Tetrahymena thermophila] | 2.00E-72 |
| scaffold_1004:74..841 | 381 | hypothetical protein TTHERM_00735290 [Tetrahymena thermophila] | 3.00E-24 |
| scaffold_1:971515..971874 | 360 | hypothetical protein TTHERM_00011250 [Tetrahymena thermophila] | 2.00E-12 |
| scaffold_1:1995373..2000788 | 1805 | hypothetical protein TTHERM_00277420 [Tetrahymena thermophila] | 7.00E-06 |
| scaffold_1:1766098..1766915 | 818 | hypothetical protein TTHERM_00016209 [Tetrahymena thermophila] | 2.00E-39 |
| scaffold_1:1548975..1549758 | 541 | Protein kinase domain containing protein [Tetrahymena thermophila] | 4.00E-05 |
| scaffold_1:1547960..1548780 | 468 | hypothetical protein TTHERM_00574290 [Tetrahymena thermophila] | 3.00E-05 |
| scaffold_1:1354873..1355609 | 678 | hypothetical protein TTHERM_00155310 [Tetrahymena thermophila] | 4.00E-24 |
