# Supplementary material for: Transcriptome Analysis of the Model Protozoan, Tetrahymena thermophila, Using Deep RNA Sequencing
Source: PLoS One. 2012 Feb 7;7(2):e30630. doi: 10.1371/journal.pone.0030630 (PMC3274533; doi:10.1371/journal.pone.0030630)
Supplement: Table S6 — Genes with state-specific alternative splicing. (DOC) [file pone.0030630.s009.doc]

**Table S6. Genes with state-specific alternative splicing**

| **Gene_ID** | **Annotation** | **AS type*** |
| --- | --- | --- |
| **A. Conjugation specific** |  |  |
| TTHERM_00365340 | AAA family ATPase, CDC48 subfamily protein | IR |
| TTHERM_00497820 | Adenylate and Guanylate cyclase catalytic domain protein | IR |
| TTHERM_01129690 | Cache domain containing protein | IR |
| TTHERM_00252390 | cation channel family protein | IR |
| TTHERM_00621480 | conserved hypothetical protein | Alt 5' SSS |
| TTHERM_00261940 | cyclic nucleotide-binding domain containing protein | Alt 3' SSS |
| TTHERM_00348590 | cyclic nucleotide-binding domain containing protein | IR |
| TTHERM_00693080 | Cyclin, N-terminal domain containing protein | IR |
| TTHERM_00420420 | DEAD/DEAH box helicase family protein | Alt 3' SSS |
| TTHERM_00470930 | DOMON domain containing protein | IR |
| TTHERM_00558310 | Dynein heavy chain family protein | Alt 5' SSS, IR |
| TTHERM_00191210 | Eukaryotic aspartyl protease family protein | IR |
| TTHERM_00565550 | FHA domain containing protein | Alt 3' SSS |
| TTHERM_00530208 | Glucose transporter family protein | IR |
| TTHERM_00412020 | Glycosyl hydrolases family 16 protein | IR |
| TTHERM_00564250 | GTP-ase activating protein for Arf containing protein | IR |
| TTHERM_01337400 | heterochromatin protein 1 | IR |
| TTHERM_00827140 | hormone sensitive lipase | Alt 5' SSS |
| TTHERM_01044400 | Integral membrane protein DUF6 containing protein | IR |
| TTHERM_01142780 | IQ calmodulin-binding motif family protein | Alt 3' SSS |
| TTHERM_00128850 | Kinesin motor domain containing protein | Alt 3' SSS |
| TTHERM_00300430 | Leucine Rich Repeat family protein | IR |
| TTHERM_00320130 | Lipase family protein | IR |
| TTHERM_00117580 | MraW methylase family protein | IR |
| TTHERM_00250940 | myb-like DNA-binding domain, SHAQKYF class family protein | IR |
| TTHERM_01166310 | N2,N2-dimethylguanosine tRNA methyltransferase | IR |
| TTHERM_00338200 | oxidoreductase, aldo/keto reductase family protein | IR |
| TTHERM_00637210 | PAS domain S-box family protein | IR |
| TTHERM_00526270 | Peptidase family C54 containing protein | IR |
| TTHERM_00561420 | PH domain containing protein | Alt 5' SSS |
| TTHERM_00684600 | phosphatidate cytidylyltransferase family protein | IR |
| TTHERM_00050510 | Protein kinase domain containing protein | Alt 3' SSS |
| TTHERM_00426130 | Protein kinase domain containing protein | Alt 5' SSS |
| TTHERM_00112560 | Protein kinase domain containing protein | IR |
| TTHERM_00585000 | Protein kinase domain containing protein | IR |
| TTHERM_01086730 | Protein kinase domain containing protein | IR |
| TTHERM_00195930 | Protein kinase domain containing protein | Alt 5' SSS |
| TTHERM_00209380 | Protein kinase domain containing protein | CEI/S |
| TTHERM_00994420 | Protein kinase domain containing protein | CEI/S |
| TTHERM_01080590 | Protein kinase domain containing protein | IR |
| TTHERM_00660130 | Protein kinase domain containing protein | IR |
| TTHERM_00085660 | Sec1 family protein | IR |
| TTHERM_00691550 | serpin, serine protease inhibitor | IR |
| TTHERM_00527160 | TBC domain containing protein | Alt 5' SSS |
| TTHERM_00766460 | Tesmin/TSO1-like CXC domain containing protein | IR |
| TTHERM_00494660 | tetrahydrofolate dehydrogenase/cyclohydrolase, NAD(P)-binding domain | Alt 5' SSS |
| TTHERM_00683400 | TPR Domain containing protein | Alt 5' SSS |
| TTHERM_00295240 | Transmembrane amino acid transporter protein | IR |
| TTHERM_00852920 | Tubulin-tyrosine ligase family protein | CEI/S |
| TTHERM_00408760 | Ubiquitin carboxyl-terminal hydrolase family protein | IR |
| TTHERM_00077170 | Ulp1 protease family, C-terminal catalytic domain containing protein | IR |
| TTHERM_00527330 | Zinc carboxypeptidase family protein | IR |
| TTHERM_00637350 | zinc finger protein | IR |
| TTHERM_00439030 | hypothetical protein | IR |
| TTHERM_00459230 | hypothetical protein | IR |
| TTHERM_00411599 | hypothetical protein | Alt 3' SSS |
| TTHERM_00112530 | hypothetical protein | Alt 3' SSS |
| TTHERM_00433480 | hypothetical protein | Alt 3' SSS |
| TTHERM_01344740 | hypothetical protein | Alt 3' SSS |
| TTHERM_00994120 | hypothetical protein | Alt 3' SSS |
| TTHERM_00046750 | hypothetical protein | Alt 3' SSS |
| TTHERM_00193790 | hypothetical protein | Alt 3' SSS |
| TTHERM_00332120 | hypothetical protein | Alt 3' SSS |
| TTHERM_00636890 | hypothetical protein | Alt 3' SSS |
| TTHERM_00420750 | hypothetical protein | Alt 5' SSS |
| TTHERM_00190658 | hypothetical protein | Alt 5' SSS |
| TTHERM_00079310 | hypothetical protein | Alt 5' SSS |
| TTHERM_00399200 | hypothetical protein | Alt 5' SSS |
| TTHERM_00285530 | hypothetical protein | Alt 5' SSS |
| TTHERM_00424560 | hypothetical protein | Alt 5' SSS |
| TTHERM_00683210 | hypothetical protein | Alt 5' SSS |
| TTHERM_00697380 | hypothetical protein | Alt 5' SSS |
| TTHERM_00299770 | hypothetical protein | Alt 5' SSS |
| TTHERM_00522860 | hypothetical protein | Alt 5' SSS |
| TTHERM_00307690 | hypothetical protein | CEI/S |
| TTHERM_00013430 | hypothetical protein | IR |
| TTHERM_00049220 | hypothetical protein | IR |
| TTHERM_00082190 | hypothetical protein | IR |
| TTHERM_00083360 | hypothetical protein | IR |
| TTHERM_00093930 | hypothetical protein | IR |
| TTHERM_00128390 | hypothetical protein | IR |
| TTHERM_00138500 | hypothetical protein | IR |
| TTHERM_00155640 | hypothetical protein | IR |
| TTHERM_00158510 | hypothetical protein | IR |
| TTHERM_00190700 | hypothetical protein | IR |
| TTHERM_00193420 | hypothetical protein | IR |
| TTHERM_00213610 | hypothetical protein | IR |
| TTHERM_00237460 | hypothetical protein | IR |
| TTHERM_00245650 | hypothetical protein | IR |
| TTHERM_00295910 | hypothetical protein | IR |
| TTHERM_00312500 | hypothetical protein | IR |
| TTHERM_00378670 | hypothetical protein | IR |
| TTHERM_00419790 | hypothetical protein | IR |
| TTHERM_00420400 | hypothetical protein | IR |
| TTHERM_00420850 | hypothetical protein | IR |
| TTHERM_00470740 | hypothetical protein | IR |
| TTHERM_00494200 | hypothetical protein | IR |
| TTHERM_00497670 | hypothetical protein | IR |
| TTHERM_00520940 | hypothetical protein | IR |
| TTHERM_00526630 | hypothetical protein | IR |
| TTHERM_00571850 | hypothetical protein | IR |
| TTHERM_00616290 | hypothetical protein | IR |
| TTHERM_00717520 | hypothetical protein | IR |
| TTHERM_00841280 | hypothetical protein | IR |
| TTHERM_00849260 | hypothetical protein | IR |
| TTHERM_00926960 | hypothetical protein | IR |
| TTHERM_00992810 | hypothetical protein | IR |
| TTHERM_01013140 | hypothetical protein | IR |
| TTHERM_01015930 | hypothetical protein | IR |
| TTHERM_00128810 | hypothetical protein | IR |
| TTHERM_00136030 | hypothetical protein | IR |
| TTHERM_00138390 | hypothetical protein | IR |
| TTHERM_00138410 | hypothetical protein | IR |
| TTHERM_00355610 | hypothetical protein | IR |
| TTHERM_00442020 | hypothetical protein | IR |
| TTHERM_00449670 | hypothetical protein | IR |
| TTHERM_00499370 | hypothetical protein | IR |
| TTHERM_00530400 | hypothetical protein | IR |
| TTHERM_00590340 | hypothetical protein | IR |
| TTHERM_00616630 | hypothetical protein | IR |
| TTHERM_00637640 | hypothetical protein | IR |
| TTHERM_00872600 | hypothetical protein | IR |
| TTHERM_01277470 | hypothetical protein | IR |
| TTHERM_00145610 | hypothetical protein | IR |
| TTHERM_00245590 | hypothetical protein | IR |
| TTHERM_00300660 | hypothetical protein | IR |
| TTHERM_00313180 | hypothetical protein | IR |
| TTHERM_00370840 | hypothetical protein | IR |
| TTHERM_00522820 | hypothetical protein | IR |
| TTHERM_01276320 | hypothetical protein | IR |
|  |  |  |
| **B. Starvation specific** |  |  |
| TTHERM_00721710 | Kinesin motor domain containing protein | Alt 3' SSS |
| TTHERM_00238790 | Kinesin motor domain containing protein | IR |
| TTHERM_00354820 | GAT domain containing protein | Alt 3' SSS |
| TTHERM_00666430 | Inosine-uridine preferring nucleoside hydrolase family protein | Alt 5' SSS |
| TTHERM_00717780 | Protein kinase domain containing protein | Alt 5' SSS |
| TTHERM_01142660 | hypothetical protein | Alt 3' SSS |
| TTHERM_00653650 | 3'-5' exonuclease family protein | Alt 3' SSS |
| TTHERM_00886960 | C2 domain containing protein | Alt 5' SSS |
| TTHERM_00622830 | DHHC zinc finger domain containing protein | Alt 5' SSS |
| TTHERM_00686190 | EF hand family protein | Alt 5' SSS |
| TTHERM_00647250 | merozoite surface protein 4/5, putative | CEI/S |
| TTHERM_00715900 | MYND finger family protein | Alt 3' SSS |
| TTHERM_00128900 | Protein kinase domain containing protein | Alt 3' SSS |
| TTHERM_00547930 | Protein kinase domain containing protein | Alt 5' SSS |
| TTHERM_00370880 | SET domain containing protein | Alt 5' SSS |
| TTHERM_00125810 | TPR Domain containing protein | Alt 5' SSS |
| TTHERM_01106200 | TPR Domain containing protein | IR |
| TTHERM_00599920 | zinc finger domain, LSD1 subclass family protein | IR |
| TTHERM_00227780 | hypothetical protein | Alt 3' SSS |
| TTHERM_00086850 | hypothetical protein | Alt 3' SSS |
| TTHERM_00277350 | hypothetical protein | Alt 3' SSS |
| TTHERM_00425820 | hypothetical protein | Alt 3' SSS |
| TTHERM_00602910 | hypothetical protein | Alt 3' SSS |
| TTHERM_00037130 | hypothetical protein | Alt 3' SSS |
| TTHERM_00442420 | hypothetical protein | Alt 3' SSS |
| TTHERM_00812640 | hypothetical protein | Alt 3' SSS, IR |
| TTHERM_00313020 | hypothetical protein | Alt 5' SSS |
| TTHERM_00348010 | hypothetical protein | Alt 5' SSS |
| TTHERM_00657630 | hypothetical protein | IR |
| TTHERM_00077280 | hypothetical protein | IR |
| TTHERM_00101180 | hypothetical protein | IR |
| TTHERM_00219230 | hypothetical protein | IR |
| TTHERM_00251050 | hypothetical protein | IR |
| TTHERM_00577010 | hypothetical protein | IR |
| TTHERM_00600360 | hypothetical protein | IR |
| TTHERM_00780680 | hypothetical protein | IR |
|  |  |  |
| **C. Growth specific** |  |  |
| TTHERM_00485980 | Helicase conserved C-terminal domain containing protein | Alt 5' SSS |
| TTHERM_01640980 | Protein kinase domain containing protein | IR |
| TTHERM_00069290 | Ubiquitin carboxyl-terminal hydrolase family protein | IR |
| TTHERM_00502290 | Viral A-type inclusion protein repeat containing protein | IR |
| TTHERM_00569320 | hypothetical protein | Alt 5' SSS |
| TTHERM_00305580 | hypothetical protein | Alt 3' SSS |

* Key to abbreviations

IR = Intron retention

Alt 5' SSS = Alternative 5’ splice-site selection

Alt 3' SSS = Alternative 3’ splice-site selection

CEI/S = Cassette-exon inclusion or skipping
