# Supplementary material for: Transcriptome Analysis of the Model Protozoan, Tetrahymena thermophila, Using Deep RNA Sequencing
Source: PLoS One. 2012 Feb 7;7(2):e30630. doi: 10.1371/journal.pone.0030630 (PMC3274533; doi:10.1371/journal.pone.0030630)
Supplement: Text S1 — Additional materials for novel and missing genes in the RNA-seq data. Section 1: 955 genes in the 2008 prediction that RNA-seq failed to detect; Section 2: 1237 novel transcribed regions identified by RNA-seq. (DOC) [file pone.0030630.s010.doc]

**Additional Materials**

**Section 1. 955 genes in the 2008 prediction that RNA-seq failed to detect**

Of the 955 gene models we failed to detect (Table S1 and Table S2), 934 were originally annotated as “hypothetical proteins”, although 40 of them now give non-Tetrahymena blast matches with expected values ranging from 1E-05 to 9E-21. 680 of the undetected gene models had a length less than 100 codons and BLASTP searches showed that all but two of them had no significant hits (E-value >1e-5) in the NBCI non-redundant protein database. Only 34 of the undetected genes had an evolutionarily conserved domain when searched against to the Pfam database. When, the expression levels of the 955 undetected genes were examined in the *Tetrahymena* Gene Expression Database (TGED, <http://tged.ihb.ac.cn/>) [1], only 140 had a maximum microarray expression level that exceeded the background level in at least one of the 20 time points distributed among the three stages of the *Tetrahymena* life cycle (Table S1 and Table S2). Among the 140 genes undetected by RNA-seq whose expression exceeded background levels in at least one microarray test, 80 recorded gene expression levels between 4 and 400 times greater than background levels. At least three reasons could be responsible for this observation. First, the microarray covered 20 states of *Tetrahymena* life cycle but only 6 of those states were covered by RNA-Seq; Second, an artifact of microarray cross-hybridization could be another reason. For example, the gene TTHERM_01853030, which showed 400 times greater than background levels in the microarray data (Table S2), has high sequence similarity (96% identity for the BLASTN high score fragment) to another gene, TTHERM_02579220, which also showed high expression level in *Tetrahymena* life cycle; Third, there may be biases for these two techniques, as some genes with high expression level in microarray data but very low expression level in RNA-Seq data were found in another study [2]. So, most of these undetected gene models are likely to be mis-predicted genes, pseudogenes, or genes transcribed only in response to untested physiological conditions

**Section 2. 1237 novel transcribed regions identified by RNA-seq**

When the 1237 novel transcribed regions were compared against the 2006 gene models (previous genome annotation), we found 217 novel transcribed regions that were present in that genome annotation but had disappeared in the 2008 version (current genome annotation) (Table S4). Among these we found some important genes, such as a histone H3 protein homolog (TTHERM_00570560) and the piwi domain-containing protein (TTHERM_00503900) (Figure 3B). The two longest novel transcribed regions are over 10 kb; one encodes a dynein heavy chain family protein (E-value: 9e-169) with 32 introns, and the other encodes a leishmanolysin family protein (E-value: 0) with 5 introns (Figure 3C). These two genes were predicted in the 2006 genome annotation as TTHERM_01151430 and TTHERM_00086980, respectively. Gene expression microarray studies using the 2006 genome annotation [3] supported the transcription of both genes and their differential expression profiles (data not shown, available at TGED).

Among the 1,237 novel transcribed regions identified by this work, 874 (about 70%) likely code for proteins: 745 of them have BLASTX expected values <1e-3 in the NCBI non-redundant database and 129 have long ORFs, covering more than 80% of the length of the assembled transcript. Some of them potentially encode important genes, such as a Sm-like protein LSm5 homolog (Figure 3D) which has been reported to associate with the U6 snRNA in spliceosome [4,5]. The remaining 363 novel transcribed regions lack recognizable ORFs, and thus potentially represent non-coding transcripts. Among the 745 novel transcribed regions with matches to the NCBI non-redundant database, more than 90% (680) have homologues in the *T. thermophila* genome, indicating that they are members of multi-gene families (Table S5).

**Reference:**

1. Xiong J, Lu X, Lu Y, Zeng H, Yuan D, et al. (2011) Tetrahymena Gene Expression Database (TGED): A resource of microarray data and co-expression analyses for Tetrahymena. SCIENCE CHINA Life Sciences 54: 65-67.

2. Marioni JC, Mason CE, Mane SM, Stephens M, Gilad Y (2008) RNA-seq: An assessment of technical reproducibility and comparison with gene expression arrays. Genome Research 18: 1509-1517.

3. Miao W, Xiong J, Bowen J, Wang W, Liu Y, et al. (2009) Microarray analyses of gene expression during the Tetrahymena thermophila life cycle. PLoS One 4: e4429.

4. Mayes AE, Verdone L, Legrain P, Beggs JD (1999) Characterization of Sm-like proteins in yeast and their association with U6 snRNA. Embo Journal 18: 4321-4331.

5. Vidal VP, Verdone L, Mayes AE, Beggs JD (1999) Characterization of U6 snRNA-protein interactions. RNA 5: 1470-1481.
